# Supplementary material for: Characterizing physiological and symptomatic variation in menstrual cycles using self-tracked mobile-health data
Source: NPJ Digit Med. 2020 May 26;3:79. doi: 10.1038/s41746-020-0269-8 (PMC7250828; doi:10.1038/s41746-020-0269-8)
Supplement: Supplementary file 1 — Supplementary Information [file 41746_2020_269_MOESM1_ESM.pdf]

Supplementary Information: Cohort and dataset

Study dataset

Supplementary Table 1: Summary statistics of this study's cohort dataset, compared with state of the art references on menstrual health studies through mobile apps.

Variable	This cohort	Cohort in (61)	Cohort in (62)
Number of users	378,694 (100.00%)	124,646 (32.92%)	212,967 (56.24%)
Number of observations	117,014,597 (100.00%)	NA	7,496,316 (6.41%)
Number of days of observation	34,056,343 (100.00%)	NA	33,675,453 (98.88%)
Number of cycles	4,881,697 (100.00%)	612,613 (12.55%)	2,732,424 (55.97%)

Supplementary Table 2: High-level characteristics for this study's cohort dataset, compared with state of the art references on menstrual health studies through mobile apps.

	Full cohort		Cohort in (61)		Cohort in (62)	
Variable	Mean ± sd	Median	Mean ± sd	Median	Mean ± sd	Median
Age	25.49 ± 3.66	25	30.3	NA	30 ± 6	NA
Number of cycles	12.89 ± 9.11	11.00	8.6	NA	12.83 (NA)	NA
Cycle length	29.73 ± 5.73	29.00	29.3 ± 5.2	NA	NA	28
Period length	4.08 ± 1.76	4.00	4.0 ± 1.5	NA	NA	NA

User demographics

Supplementary Table 3: Per-age number of users and cycles for the full cohort, as well as for the consistently not highly variable and consistently highly variable user groups.

	Full cohort		Consistently not highly variable		Consistently highly variable	
Age	Number users	Number cycles	Number users	Number cycles	Number users	Number cycles
21	71,511	557,083	65,520	526,413	5,991	30,670
22	36,723	500,736	33,338	478,394	3,385	22,342
23	33,943	466,999	30,984	447,498	2,959	19,501
24	32,225	442,053	29,529	424,706	2,696	17,347
25	30,651	422,465	28,191	406,519	2,460	15,946
26	29,377	402,905	27,066	388,306	2,311	14,599
27	27,757	380,662	25,802	368,043	1,955	12,619
28	25,257	353,535	23,518	342,245	1,739	11,290
29	22,991	325,875	21,535	316,637	1,456	9,238
30	20,744	297,814	19,462	289,725	1,282	8,089
31	18,424	269,125	17,358	262,045	1,066	7,080
32	16,444	244,483	15,521	238,957	923	5,526
33	12,647	217,962	11,782	212,206	865	5,756

Supplementary Table 4: Per-country user count in the full cohort, as well as for the consistently not highly variable and consistently highly variable user groups.

Country	Full cohort	Consistently not highly variable	Consistently highly variable
United States	97955	6911	91044
United Kingdom	32676	2486	30190

Country	Full cohort	Consistently not highly variable	Consistently highly variable
Mexico	32155	3102	29053
Brazil	27275	2535	24740
Germany	21538	1360	20178
France	19106	1371	17735
China	16529	1435	15094
Canada	15507	963	14544
Australia	14211	1103	13108
Spain	13574	804	12770
Italy	12775	685	12090
Japan	8716	692	8024
Denmark	7520	580	6940
Russia	7203	396	6807
Taiwan	5192	538	4654
Colombia	5024	475	4549
India	3976	424	3552
Switzerland	3380	216	3164
Sweden	3190	167	3023
Philippines	2876	346	2530
Argentina	2783	211	2572
Hong Kong	2706	266	2440
Singapore	2635	220	2415
South Korea	1910	205	1705
New Zealand	1902	171	1731
Peru	1897	205	1692
Netherlands	1832	135	1697
Austria	1512	117	1395
Portugal	1257	110	1147
Indonesia	1187	96	1091
Malaysia	1127	104	1023
Ireland	1115	84	1031
Chile	1080	100	980
Ecuador	1041	105	936
Turkey	835	78	757
Poland	710	43	667
Venezuela	690	51	639
Finland	482	44	438
Belgium	389	38	351
Saudi Arabia	387	27	360
Ukraine	382	29	353
Vietnam	299	42	257
Guatemala	82	12	70
South Africa	76	6	70

Cycle-statistics per user age

Supplementary Table 5: Per-age average number of cycles per user for the full cohort, as well as for the consistently not highly variable and consistently highly variable user groups.

	Full cohort		Consistently not highly variable		Consistently highly variable	
Age	Mean ± sd (95% CI)	Median	Mean ± sd (95% CI)	Median	Mean ± sd (95% CI)	Median
21	7.79 ± 3.88 (1.00,14.00)	8.00	8.03 ± 3.88 (1.00,14.00)	8.00	5.12 ± 2.63 (1.00,11.00)	5.00
22	7.74 ± 3.92 (1.00,14.00)	8.00	7.97 ± 3.92 (1.00,14.00)	8.00	4.77 ± 2.52 (1.00,11.00)	4.00
23	7.77 ± 3.94 (1.00,14.00)	8.00	8.00 ± 3.93 (1.00,14.00)	8.00	4.73 ± 2.48 (1.00,10.00)	4.00
24	7.78 ± 3.96 (1.00,14.00)	8.00	7.99 ± 3.96 (1.00,14.00)	8.00	4.74 ± 2.46 (1.00,10.00)	4.00
25	7.82 ± 3.97 (1.00,14.00)	8.00	8.03 ± 3.96 (1.00,14.00)	8.00	4.71 ± 2.47 (1.00,10.00)	4.00
26	7.85 ± 3.99 (1.00,14.00)	8.00	8.05 ± 3.98 (1.00,14.00)	8.00	4.68 ± 2.40 (1.00,10.00)	4.00
27	7.86 ± 4.02 (1.00,14.00)	8.00	8.05 ± 4.02 (1.00,14.00)	8.00	4.68 ± 2.48 (1.00,10.00)	4.00
28	7.93 ± 4.03 (1.00,14.00)	8.00	8.11 ± 4.03 (1.00,14.00)	8.00	4.70 ± 2.43 (1.00,10.00)	4.00
29	8.00 ± 4.06 (1.00,14.00)	8.00	8.18 ± 4.05 (1.00,14.00)	8.00	4.61 ± 2.41 (1.00,10.00)	4.00
30	8.08 ± 4.09 (1.00,15.00)	8.00	8.26 ± 4.08 (1.00,15.00)	9.00	4.60 ± 2.36 (1.00,10.00)	4.00
31	8.13 ± 4.11 (1.00,15.00)	8.00	8.28 ± 4.11 (1.00,15.00)	9.00	4.81 ± 2.42 (1.00,10.00)	4.00
32	8.23 ± 4.15 (1.00,15.00)	8.00	8.39 ± 4.13 (1.00,15.00)	9.00	4.56 ± 2.46 (1.00,10.00)	4.00
33	8.85 ± 3.85 (3.00,15.00)	9.00	9.05 ± 3.80 (3.00,15.00)	9.00	4.88 ± 2.25 (2.00,10.00)	4.00

Supplementary Table 6: Per-age average cycle length per user for the full cohort, as well as for the consistently not highly variable and consistently highly variable user groups.

	Full cohort		Consistently not highly variable		Consistently highly variable	
Age	Mean ± sd (95% CI)	Median	Mean ± sd (95% CI)	Median	Mean ± sd (95% CI)	Median
21	30.24 ± 6.23 (20.00,45.00)	29.00	29.86 ± 5.25 (21.00,42.00)	29.00	36.83 ± 13.66 (13.00,69.00)	34.00
22	30.16 ± 6.02 (20.00,44.00)	29.00	29.85 ± 5.20 (21.00,42.00)	29.00	36.82 ± 13.67 (13.00,69.00)	34.00
23	30.10 ± 5.95 (21.00,44.00)	29.00	29.81 ± 5.17 (21.00,42.00)	29.00	36.88 ± 13.67 (13.00,69.00)	34.00
24	30.03 ± 5.84 (21.00,44.00)	29.00	29.74 ± 5.09 (21.00,42.00)	29.00	36.96 ± 13.62 (13.00,68.00)	34.00
25	29.95 ± 5.81 (21.00,44.00)	29.00	29.66 ± 5.06 (21.00,42.00)	29.00	37.14 ± 13.76 (13.00,69.00)	34.00
26	29.85 ± 5.74 (21.00,44.00)	29.00	29.58 ± 5.00 (22.00,41.00)	29.00	37.25 ± 13.76 (13.00,69.00)	35.00
27	29.71 ± 5.65 (21.00,43.00)	29.00	29.44 ± 4.92 (22.00,41.00)	29.00	37.38 ± 13.92 (13.00,71.00)	35.00
28	29.57 ± 5.56 (22.00,43.00)	29.00	29.32 ± 4.88 (22.00,41.00)	29.00	37.27 ± 13.60 (13.00,69.00)	35.00
29	29.42 ± 5.45 (22.00,42.00)	29.00	29.18 ± 4.80 (22.00,41.00)	28.00	37.34 ± 13.99 (13.00,71.00)	34.00
30	29.24 ± 5.35 (22.00,42.00)	28.00	29.01 ± 4.71 (22.00,40.00)	28.00	37.37 ± 13.81 (14.00,70.00)	35.00
31	29.06 ± 5.23 (22.00,42.00)	28.00	28.84 ± 4.62 (22.00,40.00)	28.00	37.21 ± 13.45 (13.00,67.00)	35.00
32	28.85 ± 5.08 (22.00,41.00)	28.00	28.66 ± 4.53 (22.00,39.00)	28.00	37.10 ± 13.71 (14.00,68.00)	34.00
33	28.66 ± 5.05 (22.00,40.00)	28.00	28.45 ± 4.39 (22.00,39.00)	28.00	36.57 ± 13.74 (13.00,70.00)	33.00

Supplementary Table 7: Per-age average period length per user for the full cohort, as well as for the consistently not highly variable and consistently highly variable user groups.

	Full cohort		Consistently not highly variable		Consistently highly variable	
Age	Mean ± sd (95% CI)	Median	Mean ± sd (95% CI)	Median	Mean ± sd (95% CI)	Median
21	4.18 ± 1.74 (1.00,7.00)	4.00	4.18 ± 1.70 (1.00,7.00)	4.00	4.23 ± 2.33 (1.00,8.00)	4.00
22	4.17 ± 1.76 (1.00,7.00)	4.00	4.16 ± 1.71 (1.00,7.00)	4.00	4.36 ± 2.59 (1.00,9.00)	4.00
23	4.14 ± 1.76 (1.00,7.00)	4.00	4.13 ± 1.72 (1.00,7.00)	4.00	4.29 ± 2.55 (1.00,9.00)	4.00
24	4.12 ± 1.75 (1.00,7.00)	4.00	4.12 ± 1.71 (1.00,7.00)	4.00	4.32 ± 2.55 (1.00,9.00)	4.00
25	4.11 ± 1.75 (1.00,7.00)	4.00	4.10 ± 1.71 (1.00,7.00)	4.00	4.32 ± 2.53 (1.00,9.00)	4.00
26	4.09 ± 1.77 (1.00,7.00)	4.00	4.08 ± 1.73 (1.00,7.00)	4.00	4.34 ± 2.62 (1.00,9.00)	4.00
27	4.06 ± 1.75 (1.00,7.00)	4.00	4.05 ± 1.73 (1.00,7.00)	4.00	4.34 ± 2.39 (1.00,9.00)	4.00
28	4.04 ± 1.75 (1.00,7.00)	4.00	4.03 ± 1.72 (1.00,7.00)	4.00	4.28 ± 2.57 (1.00,9.00)	4.00
29	4.01 ± 1.76 (1.00,7.00)	4.00	4.00 ± 1.73 (1.00,7.00)	4.00	4.22 ± 2.61 (1.00,9.00)	4.00
30	3.99 ± 1.77 (1.00,7.00)	4.00	3.98 ± 1.72 (1.00,7.00)	4.00	4.28 ± 2.88 (1.00,10.00)	4.00
31	3.97 ± 1.77 (1.00,7.00)	4.00	3.97 ± 1.74 (1.00,7.00)	4.00	4.19 ± 2.73 (1.00,9.02)	4.00
32	3.95 ± 1.78 (1.00,7.00)	4.00	3.95 ± 1.76 (1.00,7.00)	4.00	4.14 ± 2.47 (1.00,9.00)	4.00
33	3.91 ± 1.78 (1.00,7.00)	4.00	3.91 ± 1.76 (1.00,7.00)	4.00	4.01 ± 2.52 (1.00,9.00)	4.00

Supplementary Table 8: Per-age average median CLD per user for the full cohort, as well as for the consistently not highly variable and consistently highly variable user groups.

	Full cohort		Consistently not highly variable		Consistently highly variable	
Age	Mean ± sd (95% CI)	Median	Mean ± sd (95% CI)	Median	Mean ± sd (95% CI)	Median
21	4.49 ± 5.07 (1.00,19.00)	3.00	3.38 ± 2.39 (1.00,9.00)	3.00	16.82 ± 9.00 (5.19,40.00)	14.00
22	4.32 ± 4.83 (1.00,17.00)	3.00	3.40 ± 2.56 (1.00,9.50)	3.00	16.32 ± 9.36 (4.00,42.00)	13.50
23	4.23 ± 4.72 (1.00,17.00)	3.00	3.36 ± 2.52 (1.00,9.00)	3.00	16.42 ± 9.23 (4.00,41.00)	14.00
24	4.10 ± 4.53 (1.00,16.00)	3.00	3.30 ± 2.51 (1.00,9.00)	2.50	16.03 ± 8.98 (3.00,39.35)	13.50
25	4.07 ± 4.57 (1.00,16.00)	3.00	3.26 ± 2.44 (1.00,9.00)	2.50	16.50 ± 9.30 (4.00,41.29)	13.50
26	3.99 ± 4.61 (1.00,16.00)	3.00	3.19 ± 2.48 (1.00,9.00)	2.50	16.59 ± 9.67 (3.00,43.00)	13.50
27	3.86 ± 4.43 (0.50,15.50)	2.50	3.13 ± 2.37 (0.50,9.00)	2.50	16.59 ± 9.54 (3.34,42.66)	14.00
28	3.81 ± 4.38 (1.00,15.00)	2.50	3.10 ± 2.39 (0.50,9.00)	2.50	16.60 ± 9.49 (4.00,43.00)	13.50
29	3.70 ± 4.25 (1.00,14.50)	2.50	3.05 ± 2.38 (0.50,9.00)	2.50	16.60 ± 9.45 (4.00,42.00)	13.50
30	3.59 ± 4.16 (1.00,14.00)	2.50	2.95 ± 2.18 (0.50,8.50)	2.00	16.73 ± 9.65 (3.00,41.00)	13.50
31	3.52 ± 4.04 (0.50,14.00)	2.50	2.92 ± 2.28 (0.50,8.50)	2.00	16.42 ± 9.00 (4.00,37.95)	14.00
32	3.42 ± 4.01 (0.50,13.00)	2.00	2.87 ± 2.22 (0.50,8.50)	2.00	16.87 ± 10.05 (3.00,43.00)	13.50
33	3.44 ± 4.25 (1.00,14.00)	2.00	2.73 ± 1.99 (1.00,8.00)	2.00	17.58 ± 9.49 (7.00,45.00)	14.00

Supplementary Table 9: Per-age average maximum CLD per user for the full cohort, as well as for the consistently not highly variable and consistently highly variable user groups.

	Full cohort		Consistently not highly variable		Consistently highly variable	
Age	Mean ± sd (95% CI)	Median	Mean ± sd (95% CI)	Median	Mean ± sd (95% CI)	Median
21	9.48 ± 7.29 (1.00,30.00)	8.00	8.18 ± 5.34 (1.00,21.00)	7.00	23.81 ± 10.10 (9.00,51.00)	22.00
22	9.14 ± 6.91 (1.00,28.00)	7.00	8.08 ± 5.26 (1.00,21.00)	7.00	23.05 ± 10.14 (7.00,50.00)	21.00
23	8.97 ± 6.84 (1.00,28.00)	7.00	7.96 ± 5.23 (1.00,21.00)	7.00	23.10 ± 10.19 (7.00,50.00)	21.00
24	8.70 ± 6.65 (1.00,27.00)	7.00	7.76 ± 5.11 (1.00,20.00)	7.00	22.78 ± 10.16 (5.00,49.00)	21.00
25	8.67 ± 6.72 (1.00,28.00)	7.00	7.72 ± 5.12 (1.00,20.00)	7.00	23.39 ± 10.26 (7.00,51.00)	22.00
26	8.51 ± 6.64 (1.00,27.00)	7.00	7.59 ± 5.09 (1.00,20.00)	6.00	23.15 ± 10.29 (6.00,50.00)	21.00
27	8.26 ± 6.47 (1.00,26.00)	7.00	7.40 ± 4.93 (1.00,19.00)	6.00	23.22 ± 10.48 (6.00,50.32)	21.00
28	8.17 ± 6.40 (1.00,26.00)	6.00	7.35 ± 4.94 (1.00,19.00)	6.00	23.09 ± 10.17 (7.00,49.15)	21.00
29	8.01 ± 6.35 (1.00,26.00)	6.00	7.24 ± 4.94 (1.00,19.00)	6.00	23.42 ± 10.31 (6.00,50.35)	22.00
30	7.82 ± 6.13 (1.00,25.00)	6.00	7.07 ± 4.71 (1.00,18.00)	6.00	23.16 ± 10.29 (7.00,50.00)	21.00
31	7.71 ± 6.04 (1.00,25.00)	6.00	7.00 ± 4.74 (1.00,18.00)	6.00	23.00 ± 9.65 (8.00,48.00)	22.00
32	7.54 ± 5.88 (1.00,24.00)	6.00	6.91 ± 4.64 (1.00,18.00)	6.00	22.94 ± 10.35 (5.00,52.00)	21.00
33	7.72 ± 6.21 (2.00,26.00)	6.00	6.90 ± 4.63 (1.00,18.00)	6.00	24.01 ± 10.10 (11.00,51.92)	22.00

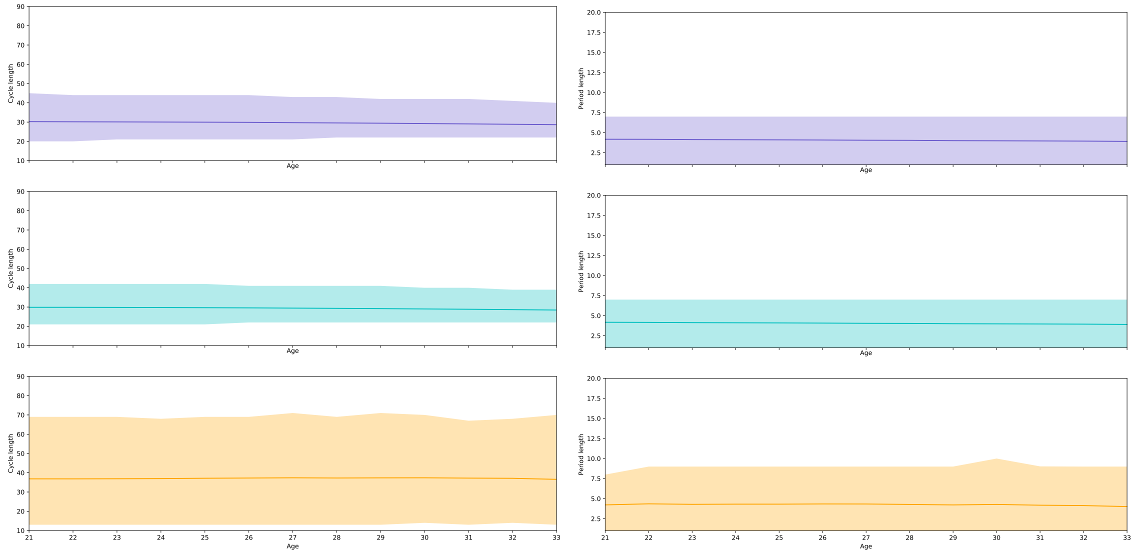

Supplementary Figure 1: For users with cycles at an specific age, we average cycle (left) and period length (right) across three different groups: the entire user cohort (top, purple), the consistently not highly variable user cohort (middle, teal), and the consistently highly variable user cohort (bottom, orange). This allows us to visualize how cycle and period length vary with age for each group, on average and in terms of standard deviation. We observe that cycle and period length statistics are stationary over the studied age range within each plot. We note that the the top and middle plots look similar in each figure (i.e., the consistently not highly variable group looks similar to the overall population in terms of both cycle and period length), but the wider shaded orange spread of the bottom plot demonstrates the higher degree of variability in the consistently highly variable group. In addition, this spread is consistently wider for all ages in the orange plot. This showcases that the consistently highly variable group represents a large degree of the variability that we see in the data overall.

Supplementary Information: Results

Assessing differences in reported symptoms across user groups

The following table provides the Kolmogorov-Smirnov statistic for the empirical cumulative distributions of the proportion of cycles with symptom out of cycles with category (λ_s) for the different user groups.

Supplementary Table 10: Kolmogorov-Smirnov test results for symptoms per-group

Category	Symptom	KS statistic (95% CI)	p-value
Period flow	heavy	0.181 (0.178,0.183)	< 0.000000
Stool health	normal	0.135 (0.130,0.140)	< 0.000000
Period flow	medium	0.134 (0.132,0.137)	< 0.000000
Social behavior	sociable	0.127 (0.121,0.132)	< 0.000000
Mental state	distracted	0.123 (0.118,0.127)	< 0.000000
Period flow	light	0.121 (0.118,0.124)	< 0.000000
Food cravings	sweet craving	0.120 (0.115,0.125)	< 0.000000
Energy level	low energy	0.118 (0.114,0.121)	< 0.000000
Motivation level	unproductive	0.117 (0.112,0.122)	< 0.000000
Digestive health	bloated	0.116 (0.111,0.122)	< 0.000000
Emotional state	sensitive	0.115 (0.112,0.118)	< 0.000000
Digestive health	gassy	0.114 (0.109,0.119)	< 0.000000
Emotional state	happy	0.108 (0.105,0.111)	< 0.000000
Mental state	calm	0.104 (0.099,0.108)	< 0.000000
Type of pain experienced	cramps	0.101 (0.097,0.104)	< 0.000000
Hours of sleep	3-6	0.100 (0.097,0.103)	< 0.000000
Food cravings	carbs craving	0.098 (0.094,0.103)	< 0.000000
Motivation level	motivated	0.098 (0.094,0.103)	< 0.000000
Motivation level	unmotivated	0.098 (0.092,0.103)	< 0.000000
Type of pain experienced	ovulation pain	0.096 (0.093,0.099)	< 0.000000
Skin health	acne skin	0.093 (0.088,0.098)	< 0.000000
Social behavior	withdrawn	0.093 (0.087,0.098)	< 0.000000
Skin health	oily skin	0.093 (0.089,0.096)	< 0.000000
Hair health	bad hair	0.092 (0.087,0.097)	< 0.000000
Vaginal discharge type	creamy	0.091 (0.086,0.095)	< 0.000000
Type of pain experienced	headache	0.089 (0.087,0.092)	< 0.000000
Hair health	good hair	0.089 (0.083,0.095)	< 0.000000
Period flow	spotting	0.089 (0.087,0.092)	< 0.000000
Emotional state	pms	0.086 (0.083,0.089)	< 0.000000
Digestive health	great digestion	0.085 (0.081,0.089)	< 0.000000
Skin health	good skin	0.085 (0.081,0.088)	< 0.000000
Food cravings	salty cravings	0.084 (0.080,0.089)	< 0.000000
Method for period collection	pad	0.083 (0.077,0.090)	< 0.000000
Type of pain experienced	tender breasts	0.082 (0.080,0.084)	< 0.000000
Hours of sleep	6-9	0.079 (0.076,0.083)	< 0.000000
Mental state	stressed	0.079 (0.074,0.083)	< 0.000000
Stool health	constipated	0.078 (0.074,0.083)	< 0.000000
Sexual health	unprotected sex	0.078 (0.074,0.081)	< 0.000000
Physical maladies	cold/flu	0.077 (0.067,0.087)	< 0.000000
Method for period collection	tampon	0.076 (0.070,0.083)	< 0.000000
Type of medication taken	cold/flu	0.076 (0.067,0.085)	< 0.000000
Emotional state	sad	0.076 (0.073,0.079)	< 0.000000
Social behavior	supportive	0.075 (0.071,0.079)	< 0.000000

Category	Symptom	KS statistic (95% CI)	p-value
Physical exercise	running	0.074 (0.067,0.081)	< 0.000000
Party-related experiences	cigarettes	0.074 (0.067,0.081)	< 0.000000
Stool health	diarrhea	0.071 (0.066,0.076)	< 0.000000
Motivation level	productive	0.071 (0.067,0.075)	< 0.000000
Food cravings	chocolate cravings	0.071 (0.066,0.075)	< 0.000000
Mental state	focused	0.069 (0.066,0.073)	< 0.000000
Vaginal discharge type	atypical	0.069 (0.065,0.074)	< 0.000000
Sexual health	protected sex	0.069 (0.065,0.073)	< 0.000000
Method for period collection	menstrual cup	0.067 (0.063,0.072)	< 0.000000
Skin health	dry skin	0.067 (0.063,0.072)	< 0.000000
Hair health	dry hair	0.067 (0.061,0.073)	< 0.000000
Hair health	oily hair	0.067 (0.062,0.072)	< 0.000000
Vaginal discharge type	sticky	0.066 (0.062,0.070)	< 0.000000
Energy level	exhausted	0.066 (0.063,0.069)	< 0.000000
Stool health	great	0.065 (0.060,0.071)	< 0.000000
Digestive health	nauseated	0.064 (0.059,0.069)	< 0.000000
Energy level	high energy	0.063 (0.061,0.066)	< 0.000000
Party-related experiences	big night party	0.063 (0.057,0.071)	< 0.000000
Social behavior	conflict	0.062 (0.059,0.068)	< 0.000000
Vaginal discharge type	egg white	0.062 (0.058,0.067)	< 0.000000
Physical exercise	yoga	0.062 (0.055,0.068)	< 0.000000
Physical maladies	allergy	0.061 (0.053,0.069)	0.000001
Hours of sleep	>9	0.061 (0.057,0.064)	< 0.000000
Method for period collection	panty liner	0.057 (0.053,0.061)	< 0.000000
Physical exercise	biking	0.056 (0.049,0.062)	< 0.000000
Party-related experiences	hangover	0.055 (0.051,0.063)	< 0.000000
Energy level	energized	0.052 (0.049,0.055)	< 0.000000
Sexual health	high sex drive	0.052 (0.051,0.055)	< 0.000000
Type of medication taken	pain	0.046 (0.041,0.054)	0.000548
Sexual health	withdrawal sex	0.045 (0.044,0.048)	< 0.000000
Physical maladies	fever	0.044 (0.037,0.054)	0.001015
Type of medication taken	antibiotic	0.044 (0.036,0.053)	0.001040
Party-related experiences	drinks party	0.042 (0.037,0.050)	0.000028
Hours of sleep	0-3	0.041 (0.039,0.044)	< 0.000000
Physical maladies	injury	0.040 (0.034,0.049)	0.003686
Physical exercise	swimming	0.040 (0.034,0.045)	0.000003
Type of medication taken	antihistamine	0.032 (0.029,0.041)	0.032955

The following table provides the odds ratio of how likely users in the consistently highly variable group to the consistently not highly variable group are not to track a symptom throughout their cycle history (i.e., in very few of their cycles).

Supplementary Table 11: Likelihood of low proportion ($\lambda_s < 0.05$) of cycles with symptom out of cycles with category per group, with the associated odds ratio. 95% confidence intervals attained via bootstrapping with 100,000 samples are shown in parentheses.

Category	Symptom	High variability group	Low variability group	Odds ratio
Period flow	medium	0.009 (0.009,0.009)	0.003 (0.003,0.003)	3.140 (2.826,3.522)
Period flow	light	0.036 (0.036,0.036)	0.014 (0.013,0.015)	2.568 (2.445,2.700)
Period flow	heavy	0.170 (0.169,0.170)	0.098 (0.096,0.100)	1.734 (1.703,1.766)
Type of pain experienced	cramps	0.105 (0.104,0.105)	0.073 (0.071,0.074)	1.436 (1.404,1.470)
Skin health	acne skin	0.174 (0.173,0.176)	0.132 (0.129,0.135)	1.319 (1.286,1.353)
Period flow	spotting	0.314 (0.313,0.315)	0.239 (0.237,0.241)	1.314 (1.300,1.328)
Mental state	stressed	0.243 (0.242,0.245)	0.186 (0.182,0.189)	1.312 (1.286,1.340)
Type of medication taken	pain	0.212 (0.209,0.215)	0.167 (0.160,0.174)	1.274 (1.220,1.334)
Emotional state	sad	0.348 (0.346,0.349)	0.273 (0.270,0.276)	1.273 (1.260,1.287)
Emotional state	pms	0.395 (0.394,0.396)	0.310 (0.307,0.313)	1.273 (1.261,1.286)
Motivation level	unmotivated	0.168 (0.167,0.170)	0.133 (0.129,0.136)	1.271 (1.237,1.307)
Party-related experiences	drinks party	0.166 (0.164,0.168)	0.131 (0.126,0.136)	1.270 (1.219,1.325)
Emotional state	sensitive	0.176 (0.175,0.177)	0.143 (0.140,0.145)	1.234 (1.214,1.254)
Stool health	diarrhea	0.369 (0.367,0.371)	0.299 (0.295,0.304)	1.234 (1.213,1.255)
Social behavior	withdrawn	0.215 (0.213,0.216)	0.176 (0.172,0.180)	1.218 (1.188,1.249)
Hours of sleep	6-9	0.161 (0.160,0.162)	0.133 (0.130,0.135)	1.218 (1.196,1.240)
Type of pain experienced	headache	0.326 (0.325,0.327)	0.269 (0.266,0.272)	1.212 (1.199,1.225)
Energy level	exhausted	0.312 (0.311,0.313)	0.258 (0.255,0.261)	1.208 (1.194,1.223)
Vaginal discharge type	egg white	0.359 (0.357,0.361)	0.298 (0.293,0.303)	1.206 (1.186,1.226)
Physical maladies	cold/flu	0.234 (0.231,0.238)	0.195 (0.187,0.202)	1.204 (1.158,1.254)
Social behavior	conflict	0.379 (0.377,0.381)	0.318 (0.313,0.323)	1.194 (1.174,1.215)
Digestive health	gassy	0.219 (0.217,0.221)	0.184 (0.180,0.188)	1.189 (1.162,1.217)
Motivation level	unproductive	0.207 (0.205,0.208)	0.175 (0.171,0.179)	1.179 (1.152,1.207)
Energy level	low energy	0.129 (0.128,0.130)	0.110 (0.108,0.112)	1.174 (1.151,1.198)
Digestive health	nauseated	0.427 (0.425,0.429)	0.365 (0.360,0.370)	1.170 (1.153,1.187)
Digestive health	bloated	0.151 (0.150,0.153)	0.130 (0.126,0.133)	1.165 (1.133,1.199)
Stool health	constipated	0.358 (0.356,0.360)	0.309 (0.304,0.314)	1.160 (1.141,1.180)
Food cravings	chocolate craving	0.350 (0.348,0.351)	0.302 (0.297,0.306)	1.159 (1.142,1.178)
Motivation level level	productive	0.354 (0.352,0.356)	0.308 (0.304,0.313)	1.148 (1.130,1.167)
Food cravings	salty craving	0.295 (0.293,0.296)	0.257 (0.253,0.261)	1.147 (1.127,1.168)
Food cravings	sweet craving	0.144 (0.143,0.146)	0.126 (0.123,0.129)	1.146 (1.116,1.178)
Type of pain experienced	tender breasts	0.366 (0.365,0.367)	0.320 (0.317,0.322)	1.145 (1.134,1.156)
Food cravings	carbs craving	0.310 (0.309,0.312)	0.271 (0.267,0.276)	1.144 (1.125,1.164)
Physical exercise	running	0.250 (0.248,0.253)	0.219 (0.214,0.224)	1.144 (1.116,1.174)
Sexual health	protected sex	0.533 (0.531,0.534)	0.466 (0.462,0.469)	1.143 (1.134,1.152)

Category	Symptom	High variability group	Low variability group	Odds ratio
Type of pain experienced	ovulation pain	0.721 (0.720,0.722)	0.633 (0.630,0.636)	1.139 (1.133,1.144)
Party-related experiences	big night party	0.522 (0.519,0.525)	0.460 (0.452,0.468)	1.136 (1.116,1.156)
Hair health	oily hair	0.363 (0.361,0.365)	0.320 (0.314,0.325)	1.135 (1.114,1.157)
Method for period collection	tampon	0.630 (0.628,0.633)	0.557 (0.551,0.563)	1.131 (1.119,1.144)
Physical exercise	yoga	0.551 (0.548,0.553)	0.489 (0.483,0.496)	1.125 (1.110,1.141)
Hair health	good hair	0.217 (0.215,0.219)	0.194 (0.189,0.199)	1.120 (1.091,1.150)
Party-related experiences	hangover	0.512 (0.509,0.515)	0.458 (0.450,0.465)	1.119 (1.100,1.139)
Stool health	great	0.595 (0.593,0.597)	0.533 (0.527,0.538)	1.118 (1.106,1.130)
Hours of sleep	3-6	0.259 (0.258,0.260)	0.232 (0.229,0.235)	1.117 (1.102,1.131)
Sexual health	high sex drive	0.469 (0.467,0.470)	0.420 (0.417,0.424)	1.115 (1.105,1.124)
Hours of sleep	>9	0.587 (0.586,0.588)	0.530 (0.526,0.533)	1.108 (1.101,1.115)
Vaginal discharge type	sticky	0.439 (0.437,0.441)	0.399 (0.394,0.404)	1.101 (1.086,1.115)
Hair health	bad hair	0.324 (0.322,0.326)	0.295 (0.289,0.300)	1.099 (1.078,1.121)
Mental state	distracted	0.204 (0.202,0.205)	0.187 (0.183,0.190)	1.091 (1.069,1.115)
Skin health	good skin	0.384 (0.382,0.386)	0.352 (0.348,0.357)	1.091 (1.076,1.105)
Vaginal discharge type	creamy	0.342 (0.340,0.344)	0.315 (0.310,0.319)	1.087 (1.071,1.105)
Sexual health	unprotected sex	0.378 (0.376,0.379)	0.348 (0.344,0.351)	1.086 (1.075,1.097)
Energy level	high energy	0.394 (0.393,0.395)	0.363 (0.360,0.367)	1.085 (1.075,1.095)
Physical exercise	biking	0.715 (0.712,0.717)	0.660 (0.654,0.666)	1.083 (1.072,1.093)
Method for period collection	menstrual cup	0.880 (0.879,0.882)	0.814 (0.809,0.818)	1.082 (1.075,1.088)
Mental state	focused	0.407 (0.405,0.409)	0.377 (0.372,0.381)	1.081 (1.067,1.095)
Type of medication taken	cold/flu	0.569 (0.565,0.573)	0.527 (0.517,0.536)	1.080 (1.060,1.101)
Motivation level	motivated	0.299 (0.297,0.301)	0.278 (0.273,0.282)	1.075 (1.057,1.094)
Sexual health	withdrawal sex	0.596 (0.595,0.598)	0.556 (0.552,0.559)	1.073 (1.065,1.080)
Social behavior	supportive	0.412 (0.410,0.414)	0.386 (0.380,0.391)	1.069 (1.054,1.085)
Physical maladies	fever	0.704 (0.701,0.708)	0.661 (0.653,0.670)	1.065 (1.050,1.080)
Hair health	dry hair	0.441 (0.439,0.443)	0.415 (0.409,0.421)	1.063 (1.047,1.079)
Type of medication taken	antibiotic	0.712 (0.709,0.716)	0.671 (0.662,0.680)	1.061 (1.047,1.076)
Skin health	dry skin	0.493 (0.491,0.494)	0.464 (0.460,0.469)	1.060 (1.049,1.072)
Physical maladies	injury	0.732 (0.728,0.735)	0.692 (0.684,0.701)	1.057 (1.044,1.071)
Energy level	energized	0.625 (0.624,0.626)	0.593 (0.590,0.596)	1.054 (1.047,1.060)
Method for period collection	panty liner	0.553 (0.551,0.555)	0.525 (0.519,0.531)	1.053 (1.040,1.066)
Skin health	oily skin	0.372 (0.371,0.374)	0.355 (0.351,0.360)	1.048 (1.034,1.062)
Physical exercise	swimming	0.841 (0.840,0.843)	0.803 (0.798,0.808)	1.047 (1.040,1.054)
Hours of sleep	0-3	0.762 (0.761,0.763)	0.731 (0.728,0.734)	1.043 (1.038,1.047)
Type of medication taken	antihistamine	0.767 (0.763,0.770)	0.736 (0.727,0.744)	1.042 (1.030,1.055)
Social behavior	sociable	0.218 (0.217,0.220)	0.210 (0.206,0.215)	1.038 (1.015,1.062)

Category	Symptom	High variability group	Low variability group	Odds ratio
Physical maladies	allergy	0.581 (0.578,0.585)	0.560 (0.551,0.569)	1.037 (1.019,1.056)
Emotional state	happy	0.281 (0.280,0.282)	0.275 (0.272,0.278)	1.024 (1.013,1.035)
Mental state	calm	0.293 (0.292,0.295)	0.290 (0.286,0.295)	1.010 (0.995,1.027)
Digestive health	great digestion	0.388 (0.386,0.390)	0.388 (0.383,0.393)	1.002 (0.988,1.016)
Stool health	normal	0.181 (0.179,0.182)	0.181 (0.177,0.185)	0.998 (0.975,1.022)
Vaginal discharge type	atypical	0.664 (0.662,0.666)	0.673 (0.668,0.678)	0.986 (0.978,0.993)
Party-related experiences	cigarettes	0.581 (0.578,0.585)	0.608 (0.601,0.616)	0.956 (0.943,0.969)
Method for period collection	pad	0.214 (0.212,0.216)	0.236 (0.231,0.241)	0.907 (0.886,0.929)

The following table provides the odds ratio of how likely users in the consistently highly variable group to the consistently not highly variable group are to consistently track a symptom throughout their cycle history (i.e., in almost every cycle where they track the category).

Supplementary Table 12: Likelihood of high proportion ($\lambda_s > 0.95$) of cycles with symptom out of cycles with category per group, with the associated odds ratio. 95% confidence intervals attained via bootstrapping with 100,000 samples are shown in parentheses.

Category	Symptom	High variability group	Low variability group	Odds ratio
Hours of sleep	0-3	0.035 (0.034,0.035)	0.020 (0.019,0.021)	1.750 (1.667,1.839)
Period flow	spotting	0.067 (0.066,0.067)	0.039 (0.037,0.040)	1.729 (1.679,1.782)
Type of pain experienced	tender breasts	0.193 (0.192,0.194)	0.113 (0.111,0.115)	1.715 (1.684,1.746)
Vaginal discharge type	atypical	0.100 (0.099,0.101)	0.059 (0.056,0.061)	1.706 (1.636,1.780)
Energy level	energized	0.075 (0.074,0.075)	0.044 (0.043,0.046)	1.686 (1.633,1.741)
Type of pain experienced	headache	0.218 (0.217,0.219)	0.131 (0.129,0.133)	1.663 (1.636,1.691)
Skin health	dry skin	0.155 (0.154,0.157)	0.096 (0.093,0.098)	1.626 (1.579,1.676)
Type of medication taken	cold/flu	0.179 (0.176,0.182)	0.112 (0.107,0.118)	1.590 (1.506,1.681)
Skin health	oily skin	0.250 (0.248,0.251)	0.159 (0.155,0.162)	1.575 (1.540,1.611)
Hair health	dry hair	0.170 (0.169,0.172)	0.109 (0.105,0.113)	1.565 (1.510,1.624)
Digestive health	great digestion	0.241 (0.239,0.243)	0.158 (0.154,0.162)	1.528 (1.490,1.567)
Social behavior	supportive	0.215 (0.213,0.216)	0.141 (0.138,0.145)	1.519 (1.477,1.562)
Emotional state	happy	0.307 (0.306,0.308)	0.202 (0.200,0.205)	1.518 (1.498,1.538)
Skin health	good skin	0.242 (0.241,0.244)	0.160 (0.156,0.163)	1.518 (1.485,1.552)
Hair health	bad hair	0.266 (0.264,0.268)	0.175 (0.171,0.180)	1.514 (1.474,1.557)
Digestive health	nauseated	0.170 (0.168,0.171)	0.112 (0.109,0.116)	1.511 (1.466,1.558)
Stool health	great	0.101 (0.100,0.102)	0.068 (0.065,0.071)	1.487 (1.428,1.549)
Emotional state	sad	0.171 (0.170,0.172)	0.115 (0.113,0.117)	1.486 (1.459,1.513)
Method for period collection	panty liner	0.174 (0.172,0.175)	0.118 (0.114,0.122)	1.471 (1.422,1.523)
Stool health	constipated	0.246 (0.244,0.248)	0.169 (0.165,0.173)	1.454 (1.420,1.491)
Mental state	focused	0.218 (0.216,0.219)	0.150 (0.147,0.153)	1.451 (1.417,1.486)
Mental state	calm	0.327 (0.325,0.328)	0.225 (0.221,0.229)	1.450 (1.424,1.477)
Vaginal discharge type	sticky	0.214 (0.212,0.216)	0.148 (0.145,0.152)	1.442 (1.406,1.479)
Type of medication taken	antihistamine	0.099 (0.096,0.101)	0.069 (0.064,0.074)	1.437 (1.337,1.548)
Hours of sleep	3-6	0.322 (0.321,0.324)	0.225 (0.222,0.228)	1.431 (1.413,1.450)
Motivation level	motivated	0.321 (0.319,0.322)	0.225 (0.220,0.229)	1.428 (1.401,1.457)
Hours of sleep	>9	0.093 (0.092,0.094)	0.065 (0.064,0.067)	1.425 (1.388,1.464)
Physical exercise	swimming	0.061 (0.060,0.062)	0.043 (0.040,0.045)	1.423 (1.339,1.516)
Motivation level	unproductive	0.387 (0.386,0.389)	0.272 (0.268,0.277)	1.422 (1.398,1.447)
Mental state	distracted	0.407 (0.405,0.409)	0.286 (0.282,0.290)	1.422 (1.400,1.444)
Type of pain experienced	ovulation pain	0.044 (0.043,0.044)	0.031 (0.030,0.032)	1.419 (1.369,1.473)
Emotional state	sensitive	0.380 (0.378,0.381)	0.269 (0.266,0.272)	1.411 (1.395,1.426)
Food cravings	carbs craving	0.334 (0.332,0.336)	0.238 (0.234,0.242)	1.403 (1.378,1.429)
Energy level	high energy	0.214 (0.213,0.215)	0.153 (0.150,0.155)	1.400 (1.377,1.423)
Social behavior	conflict	0.208 (0.206,0.210)	0.149 (0.145,0.153)	1.399 (1.362,1.438)

Category	Symptom	High variability group	Low variability group	Odds ratio
Vaginal discharge type	creamy	0.314 (0.312,0.316)	0.224 (0.220,0.228)	1.399 (1.372,1.427)
Social behavior	sociable	0.444 (0.442,0.446)	0.320 (0.315,0.325)	1.388 (1.365,1.411)
Sexual health	withdrawal sex	0.159 (0.158,0.160)	0.115 (0.112,0.117)	1.386 (1.358,1.415)
Energy level	exhausted	0.235 (0.234,0.236)	0.170 (0.167,0.172)	1.382 (1.361,1.403)
Stool health	normal	0.475 (0.473,0.477)	0.344 (0.339,0.349)	1.381 (1.361,1.402)
Digestive health	gassy	0.400 (0.398,0.402)	0.290 (0.285,0.294)	1.381 (1.358,1.405)
Hair health	oily hair	0.244 (0.242,0.246)	0.178 (0.173,0.183)	1.368 (1.332,1.407)
Physical maladies	fever	0.119 (0.116,0.121)	0.087 (0.082,0.092)	1.368 (1.285,1.458)
Emotional state	pms	0.160 (0.159,0.161)	0.117 (0.115,0.119)	1.367 (1.342,1.393)
Food cravings	chocolate craving	0.263 (0.261,0.264)	0.194 (0.190,0.198)	1.357 (1.329,1.386)
Motivation level	productive	0.266 (0.264,0.267)	0.197 (0.193,0.201)	1.347 (1.318,1.376)
Physical maladies	injury	0.105 (0.102,0.107)	0.078 (0.073,0.083)	1.346 (1.260,1.442)
Type of medication taken	antibiotic	0.123 (0.120,0.126)	0.092 (0.086,0.097)	1.345 (1.264,1.433)
Party-related experiences	hangover	0.200 (0.198,0.203)	0.149 (0.144,0.155)	1.343 (1.293,1.397)
Physical maladies	allergy	0.236 (0.233,0.239)	0.176 (0.169,0.183)	1.343 (1.289,1.402)
Party-related experiences	big night party	0.215 (0.212,0.217)	0.160 (0.154,0.166)	1.342 (1.293,1.393)
Party-related experiences	cigarettes	0.290 (0.287,0.293)	0.217 (0.211,0.223)	1.337 (1.297,1.379)
Stool health	diarrhea	0.225 (0.223,0.226)	0.169 (0.165,0.173)	1.330 (1.298,1.363)
Food cravings	salty craving	0.331 (0.330,0.333)	0.249 (0.245,0.253)	1.330 (1.307,1.353)
Energy level	low energy	0.489 (0.488,0.491)	0.376 (0.373,0.379)	1.302 (1.290,1.314)
Social behavior	withdrawn	0.397 (0.395,0.399)	0.307 (0.302,0.312)	1.294 (1.272,1.317)
Sexual health	high sex drive	0.224 (0.223,0.226)	0.174 (0.171,0.176)	1.292 (1.271,1.313)
Digestive health	bloated	0.502 (0.500,0.504)	0.390 (0.385,0.395)	1.287 (1.270,1.305)
Food cravings	sweet craving	0.527 (0.526,0.529)	0.411 (0.406,0.416)	1.283 (1.268,1.299)
Mental state	stressed	0.353 (0.351,0.354)	0.276 (0.272,0.280)	1.277 (1.257,1.298)
Sexual health	unprotected sex	0.354 (0.353,0.356)	0.279 (0.276,0.282)	1.271 (1.256,1.286)
Motivation level	unmotivated	0.446 (0.444,0.448)	0.352 (0.347,0.356)	1.270 (1.251,1.288)
Hair health	good hair	0.421 (0.419,0.424)	0.336 (0.331,0.342)	1.253 (1.231,1.276)
Period flow	light	0.250 (0.249,0.251)	0.203 (0.200,0.205)	1.233 (1.219,1.248)
Skin health	acne skin	0.489 (0.487,0.491)	0.400 (0.395,0.405)	1.222 (1.207,1.237)
Vaginal discharge type	egg white	0.298 (0.297,0.300)	0.244 (0.240,0.249)	1.222 (1.199,1.245)
Type of pain experienced	cramps	0.529 (0.528,0.530)	0.442 (0.439,0.445)	1.198 (1.189,1.206)
Sexual health	protected sex	0.219 (0.218,0.220)	0.183 (0.181,0.186)	1.196 (1.178,1.215)
Physical exercise	biking	0.129 (0.128,0.131)	0.109 (0.105,0.113)	1.188 (1.144,1.235)
Hours of sleep	6-9	0.474 (0.473,0.476)	0.400 (0.396,0.403)	1.188 (1.177,1.198)
Physical exercise	yoga	0.262 (0.260,0.265)	0.223 (0.217,0.228)	1.179 (1.151,1.209)
Physical maladies	cold/flu	0.529 (0.525,0.533)	0.453 (0.444,0.462)	1.169 (1.144,1.194)
Method for period collection	pad	0.583 (0.581,0.585)	0.505 (0.499,0.511)	1.155 (1.141,1.170)
Physical exercise	running	0.563 (0.560,0.566)	0.490 (0.484,0.496)	1.149 (1.133,1.164)
Period flow	medium	0.388 (0.387,0.389)	0.345 (0.342,0.347)	1.126 (1.117,1.136)

Category	Symptom	High variability group	Low variability group	Odds ratio
Party-related experiences	drinks party	0.635 (0.632,0.638)	0.594 (0.587,0.602)	1.069 (1.055,1.084)
Type of medication taken	pain	0.597 (0.593,0.601)	0.561 (0.552,0.571)	1.063 (1.044,1.082)
Method for period collection	tampon	0.210 (0.209,0.212)	0.218 (0.213,0.223)	0.967 (0.943,0.991)
Period flow	heavy	0.078 (0.077,0.079)	0.096 (0.094,0.097)	0.817 (0.802,0.833)
Method for period collection	menstrual cup	0.075 (0.074,0.076)	0.100 (0.096,0.103)	0.755 (0.726,0.785)

The following figures showcase the empirical cumulative distributions of the proportion of cycles with symptom out of cycles with category between different user groups — the consistently highly variable group is indicated in orange, and the consistently not highly variable group is indicated in teal. Figures are organized based on their Kolmogorov-Smirnov test value, in descending order. The mean (dotted line) and %95 confidence interval (shaded region) of the bootstrapped CDF with 100,000 samples is also shown.

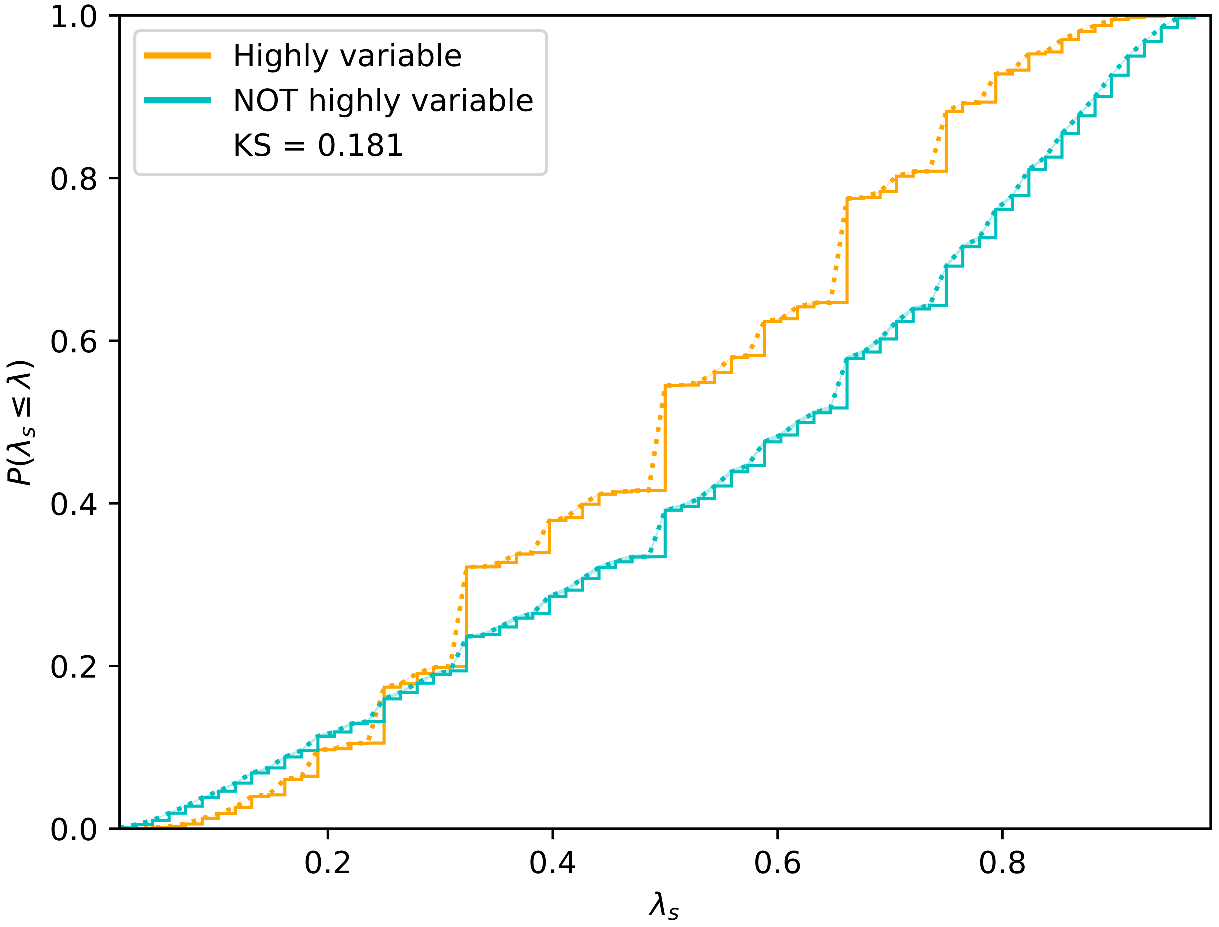

Supplementary Figure 2: Heavy period flow.

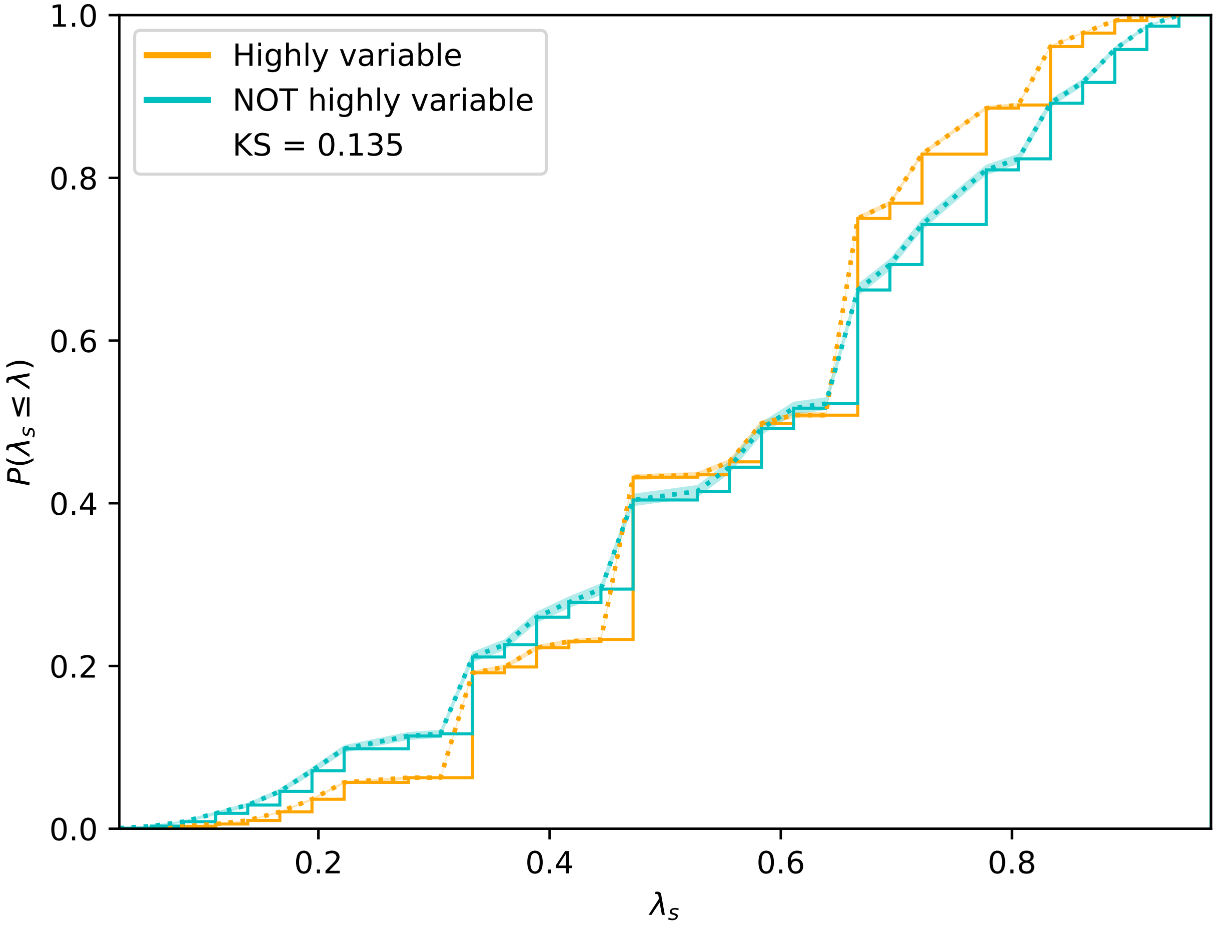

Supplementary Figure 3: Normal stool health.

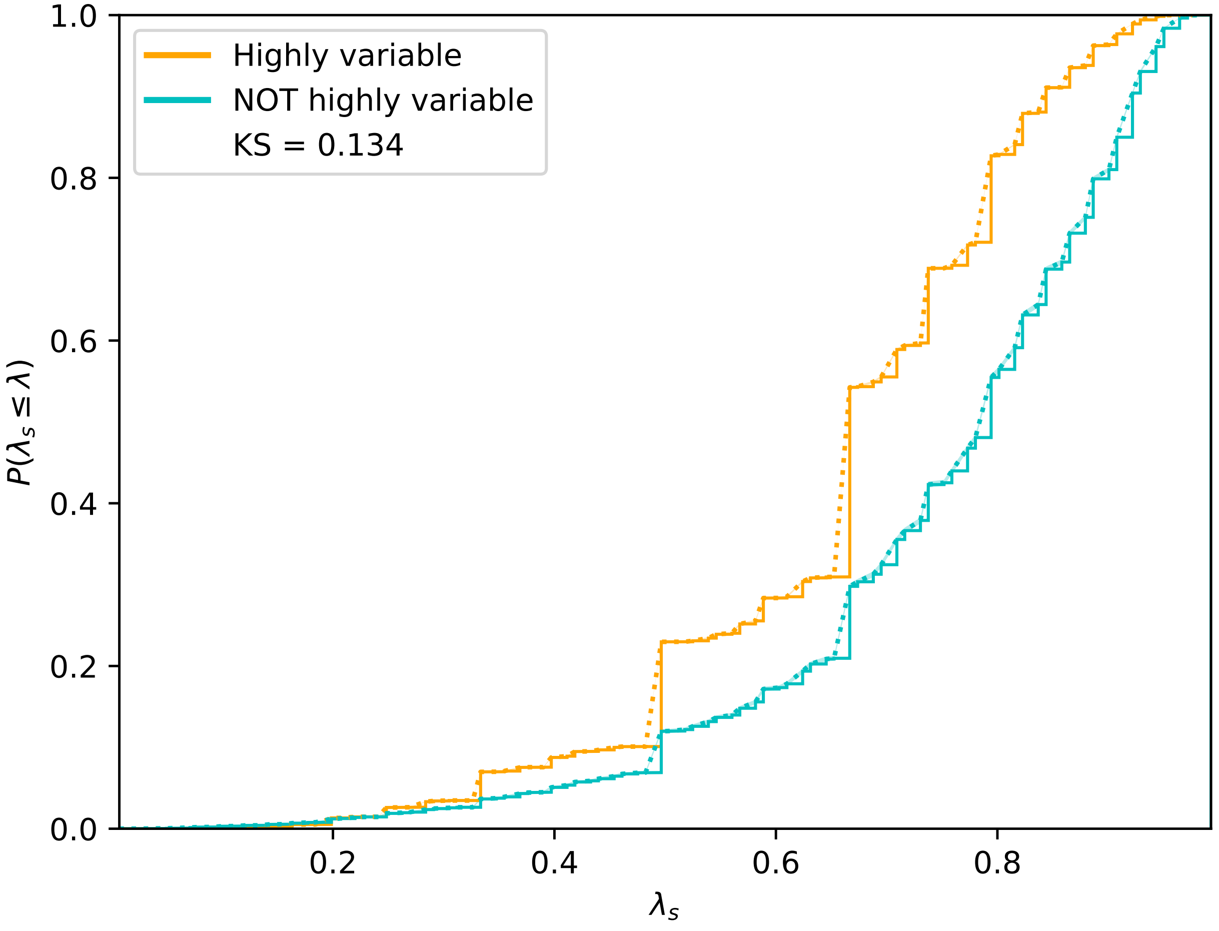

Supplementary Figure 4: Medium period flow.

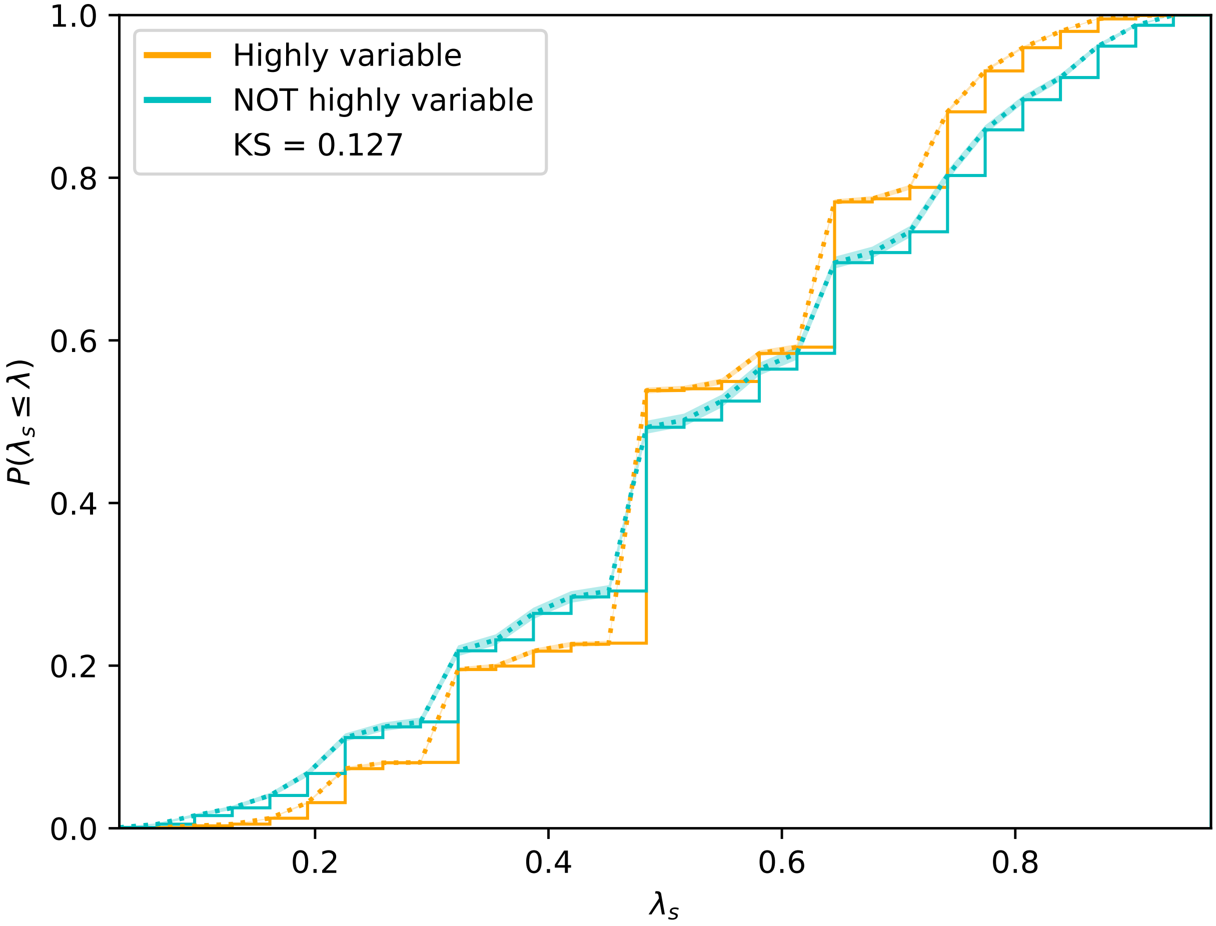

Supplementary Figure 5: Sociable social behavior.

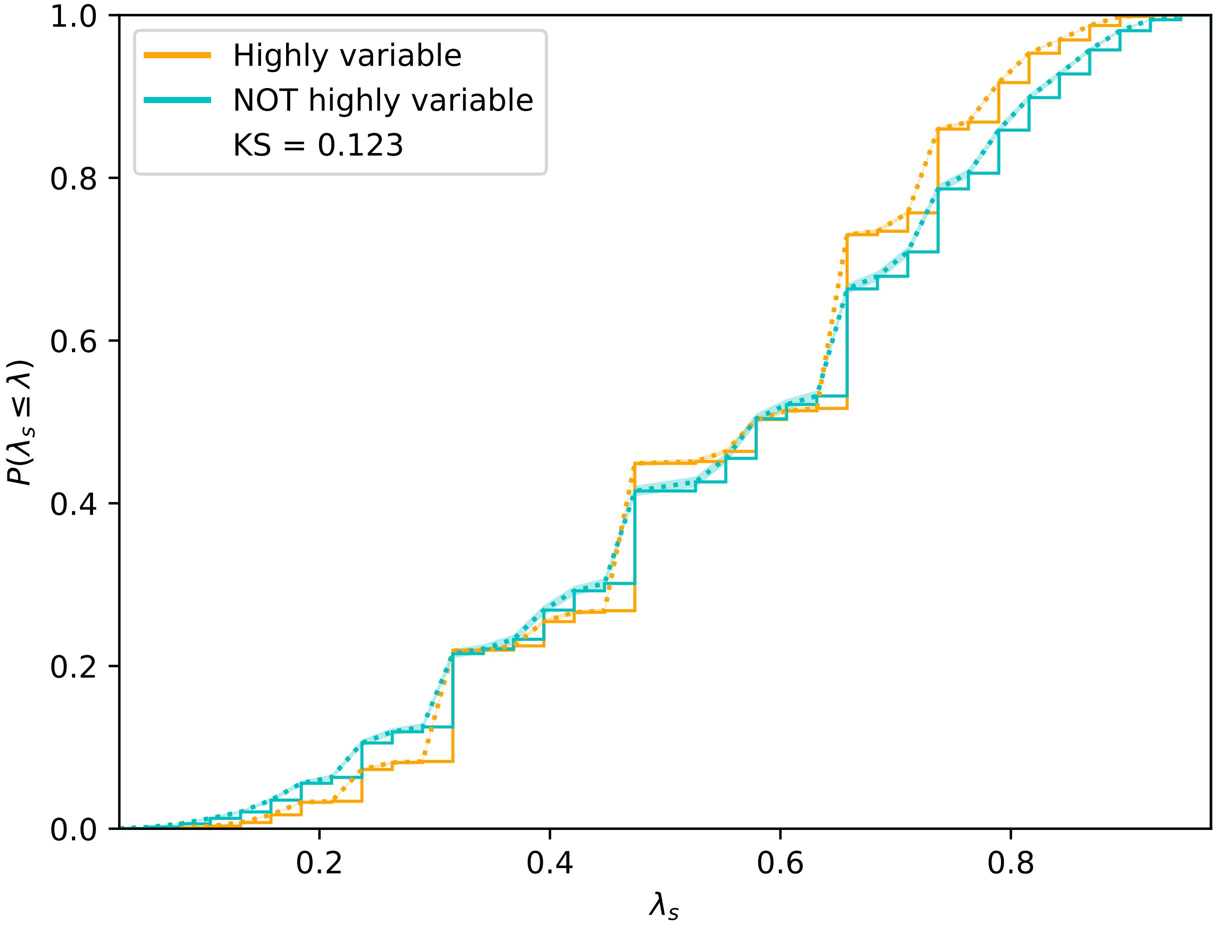

Supplementary Figure 6: Distracted mental state.

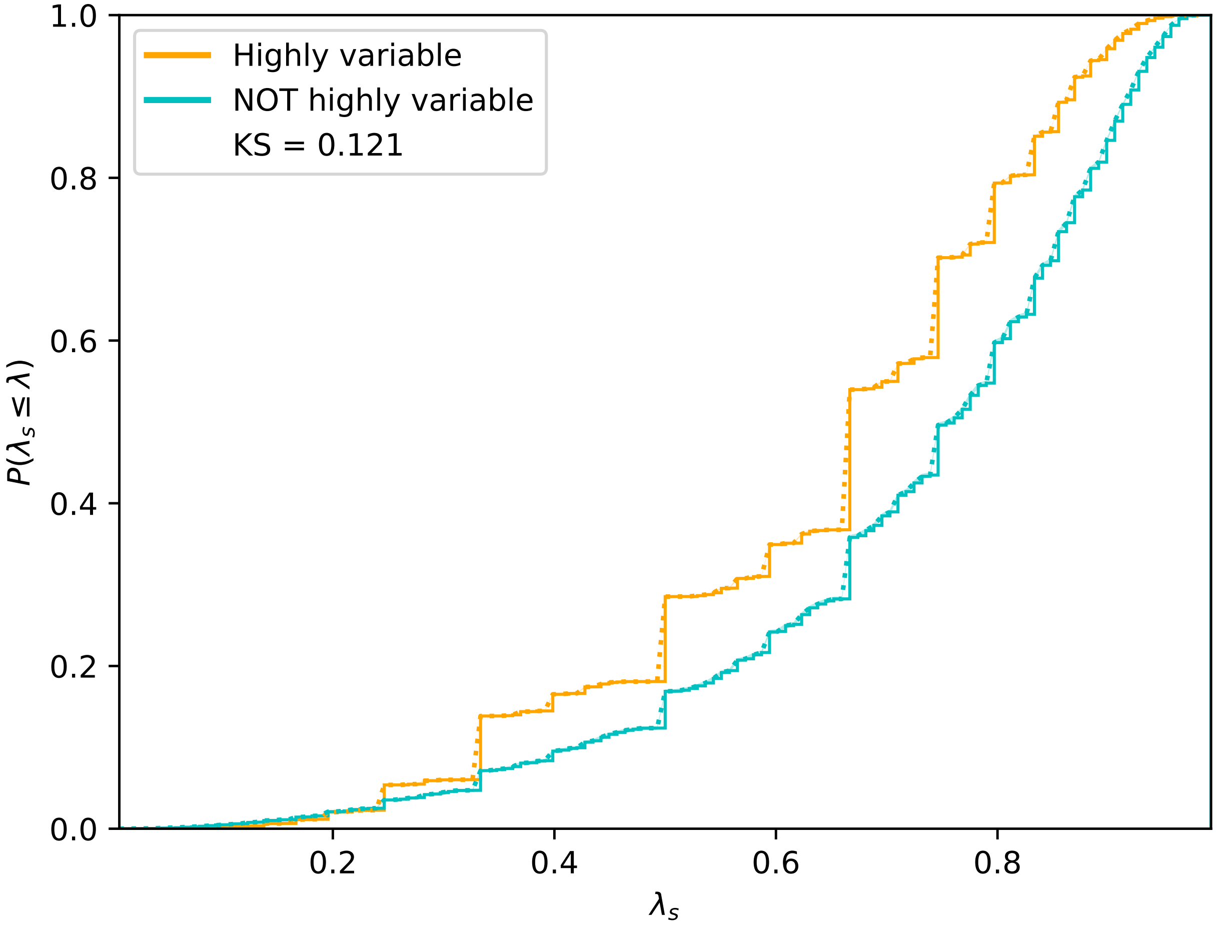

Supplementary Figure 7: Light period flow.

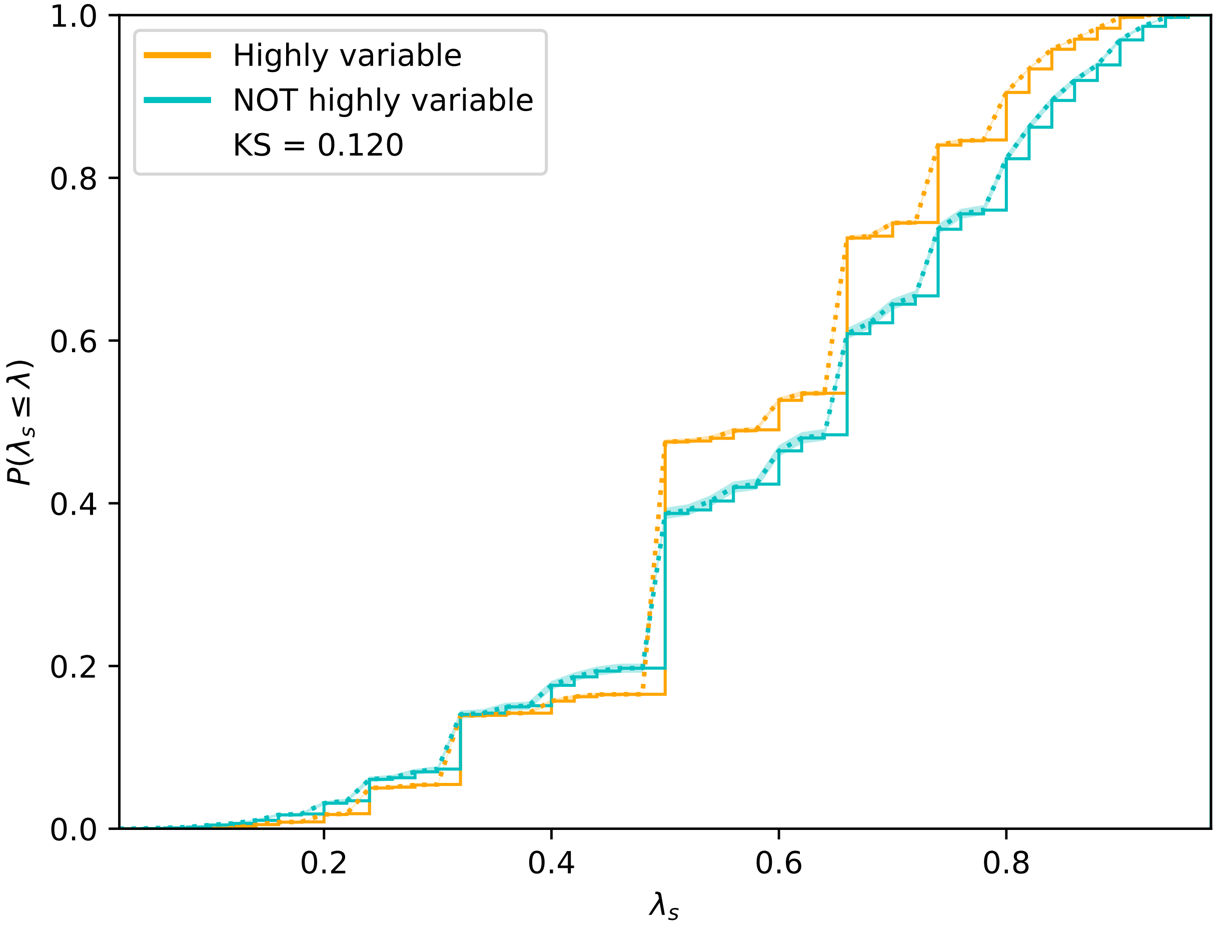

Supplementary Figure 8: Sweet food craving experienced.

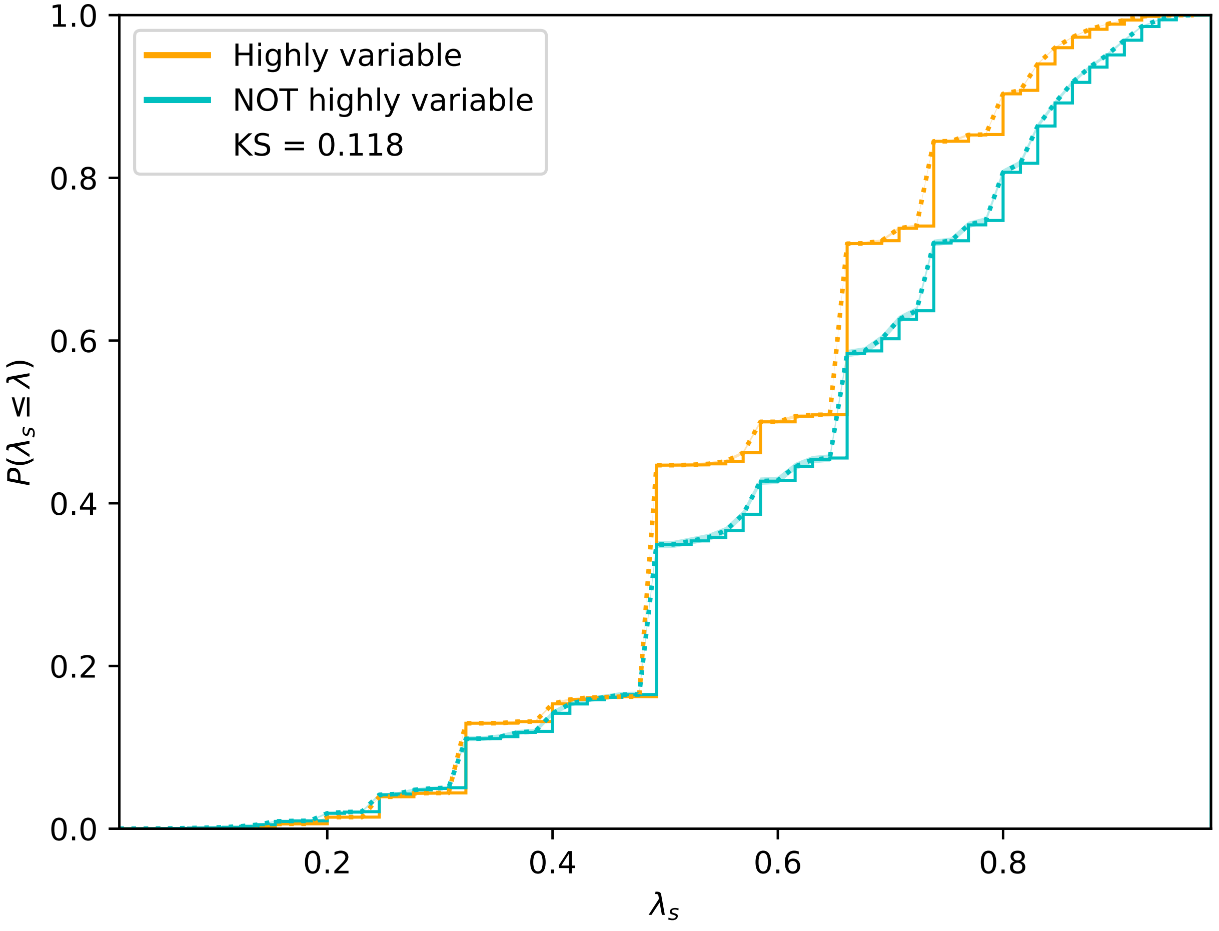

Supplementary Figure 9: Low energy level.

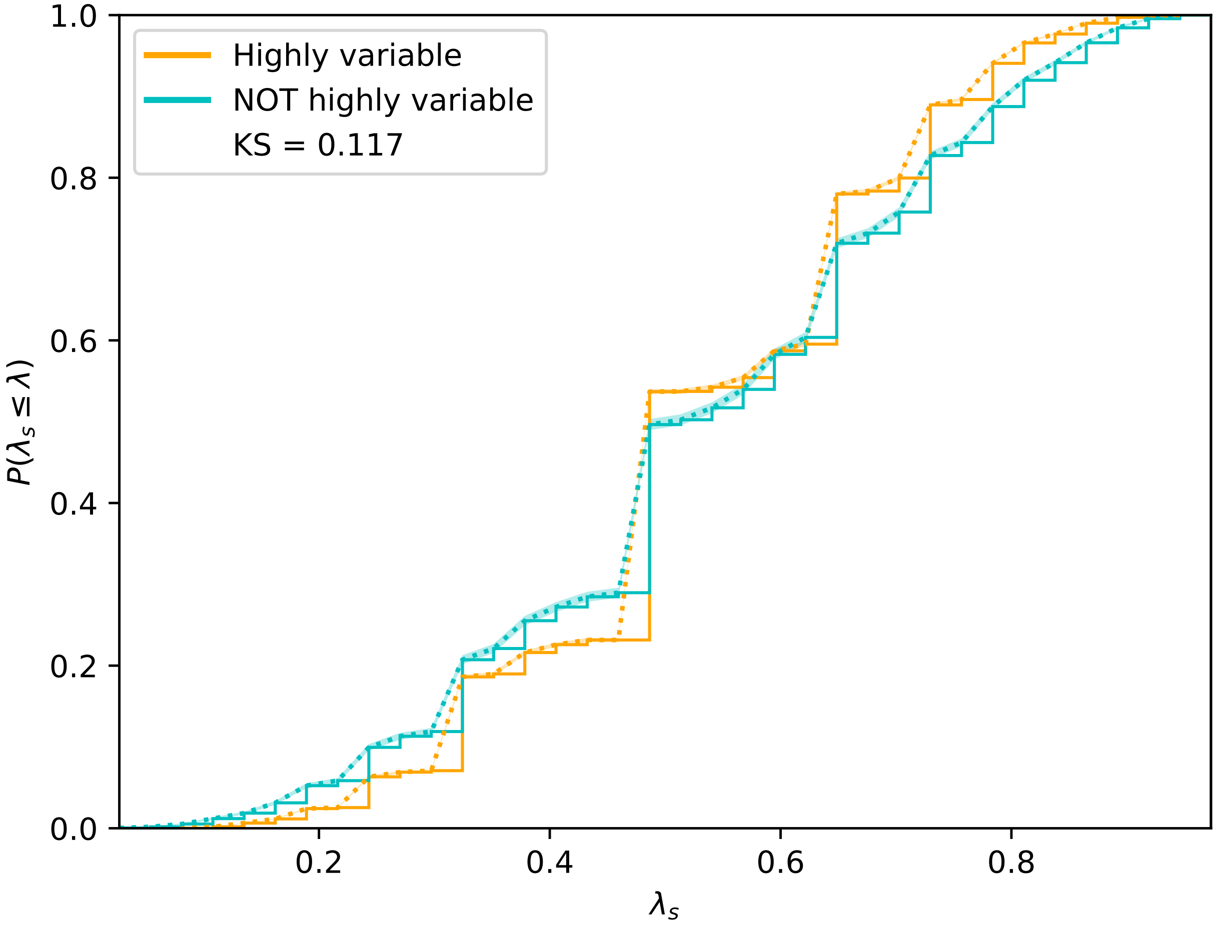

Supplementary Figure 10: Unproductive motivation level.

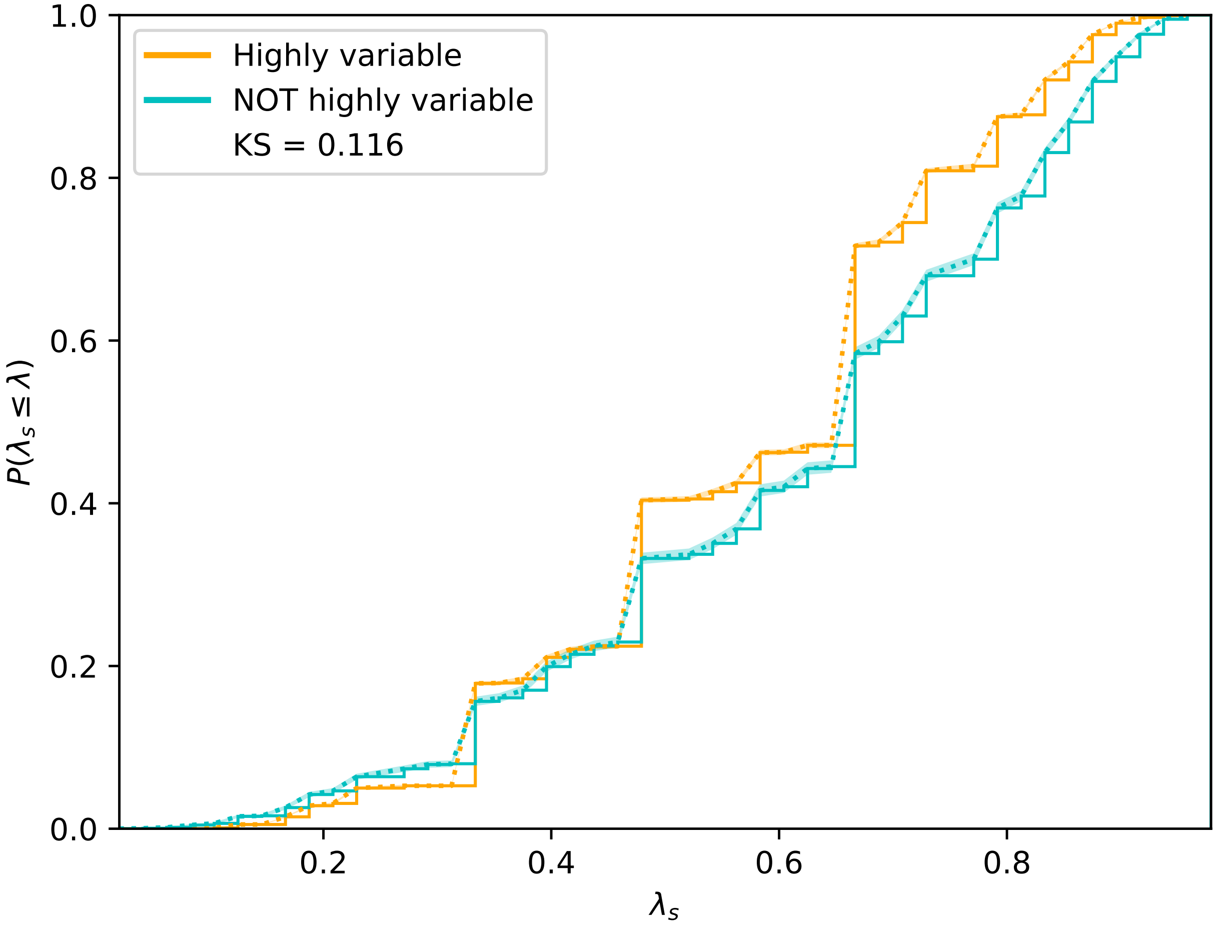

Supplementary Figure 11: Bloated digestive health.

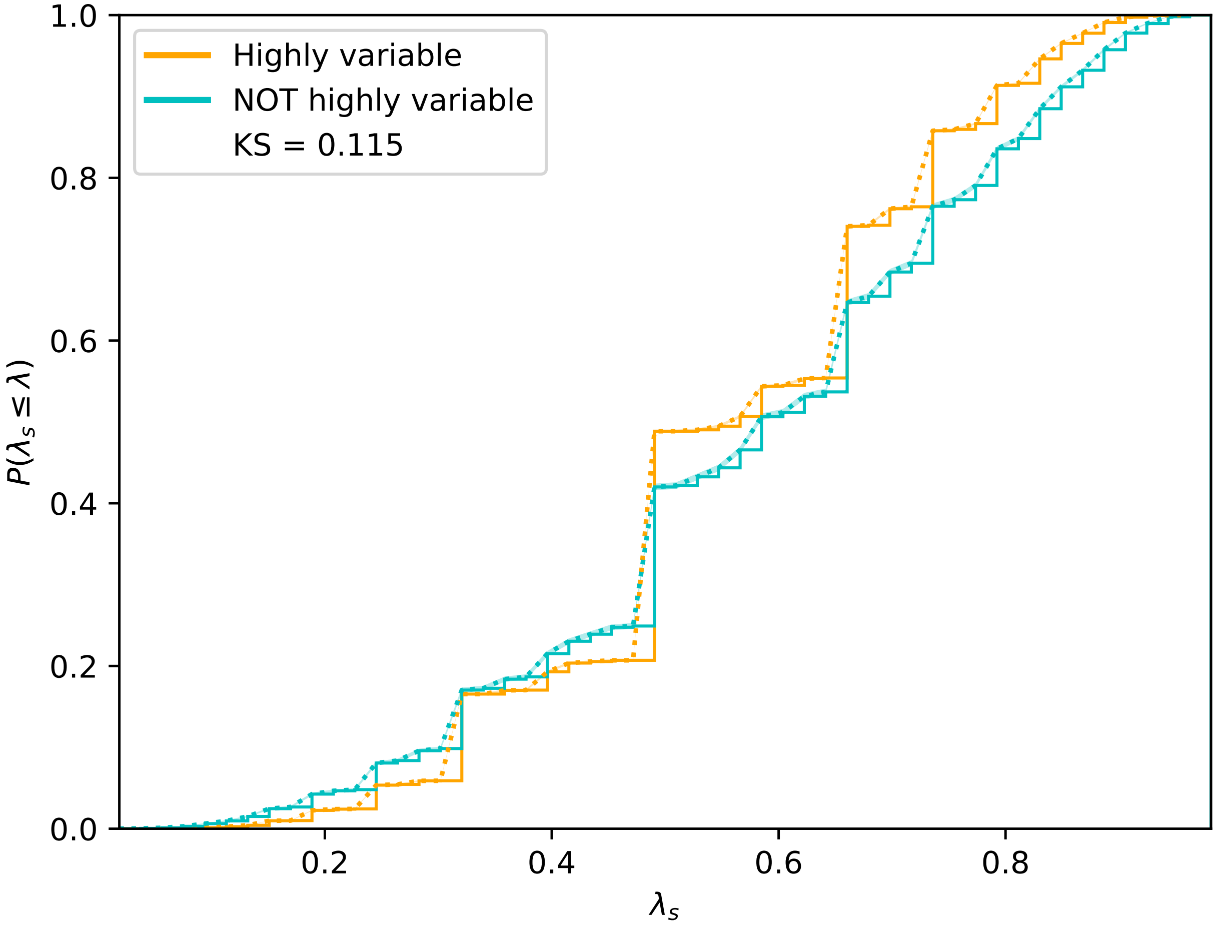

Supplementary Figure 12: Sensitive emotional state.

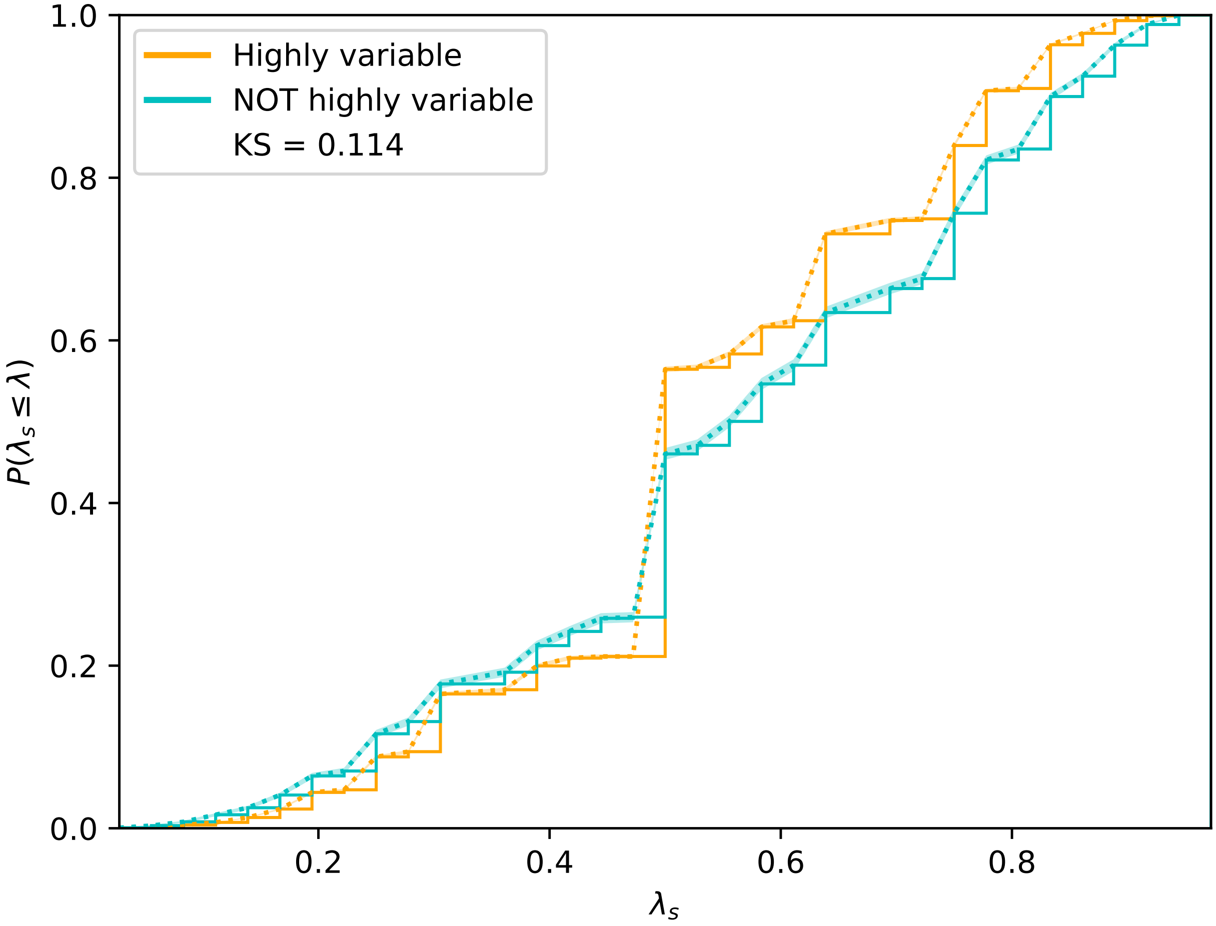

Supplementary Figure 13: Gassy digestive health.

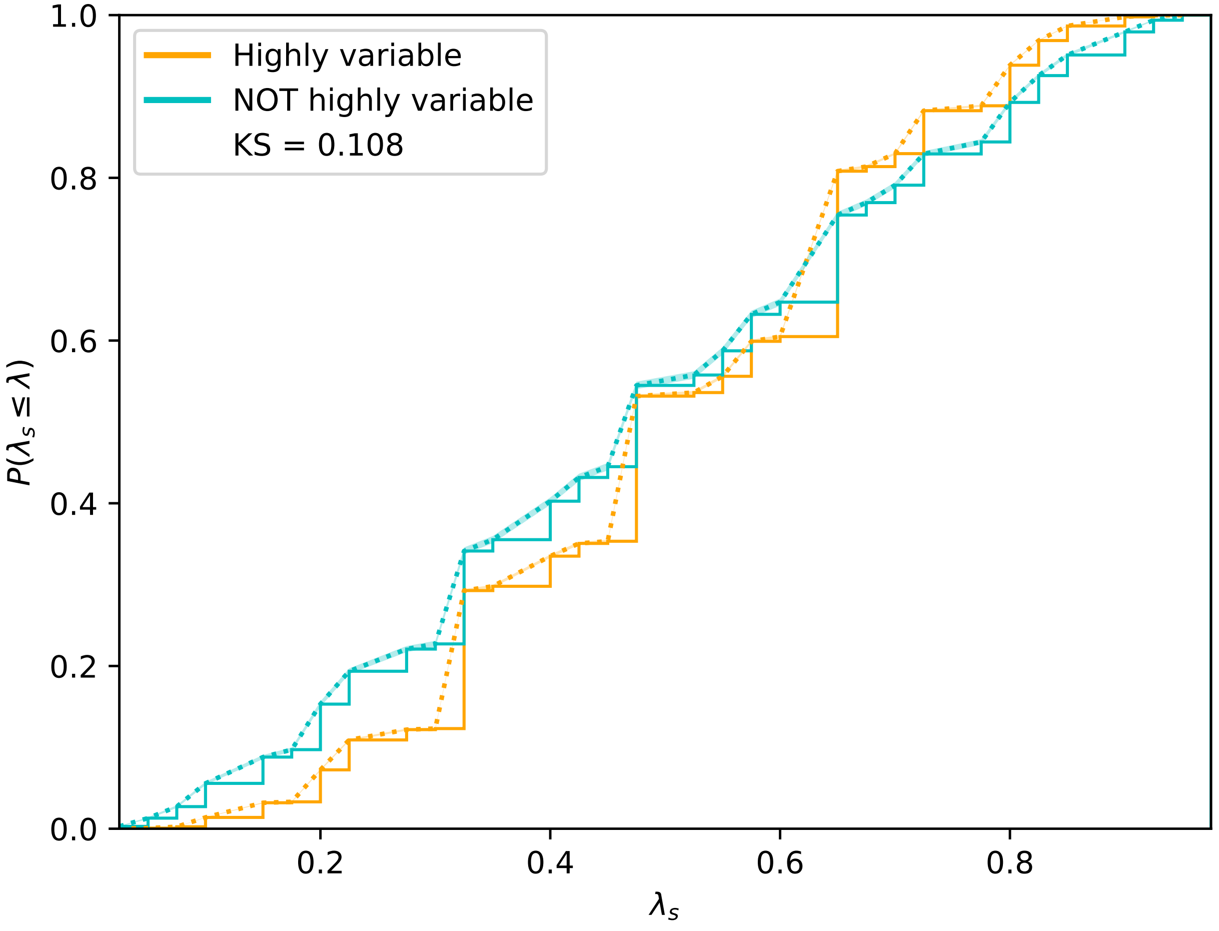

Supplementary Figure 14: Happy emotional state.

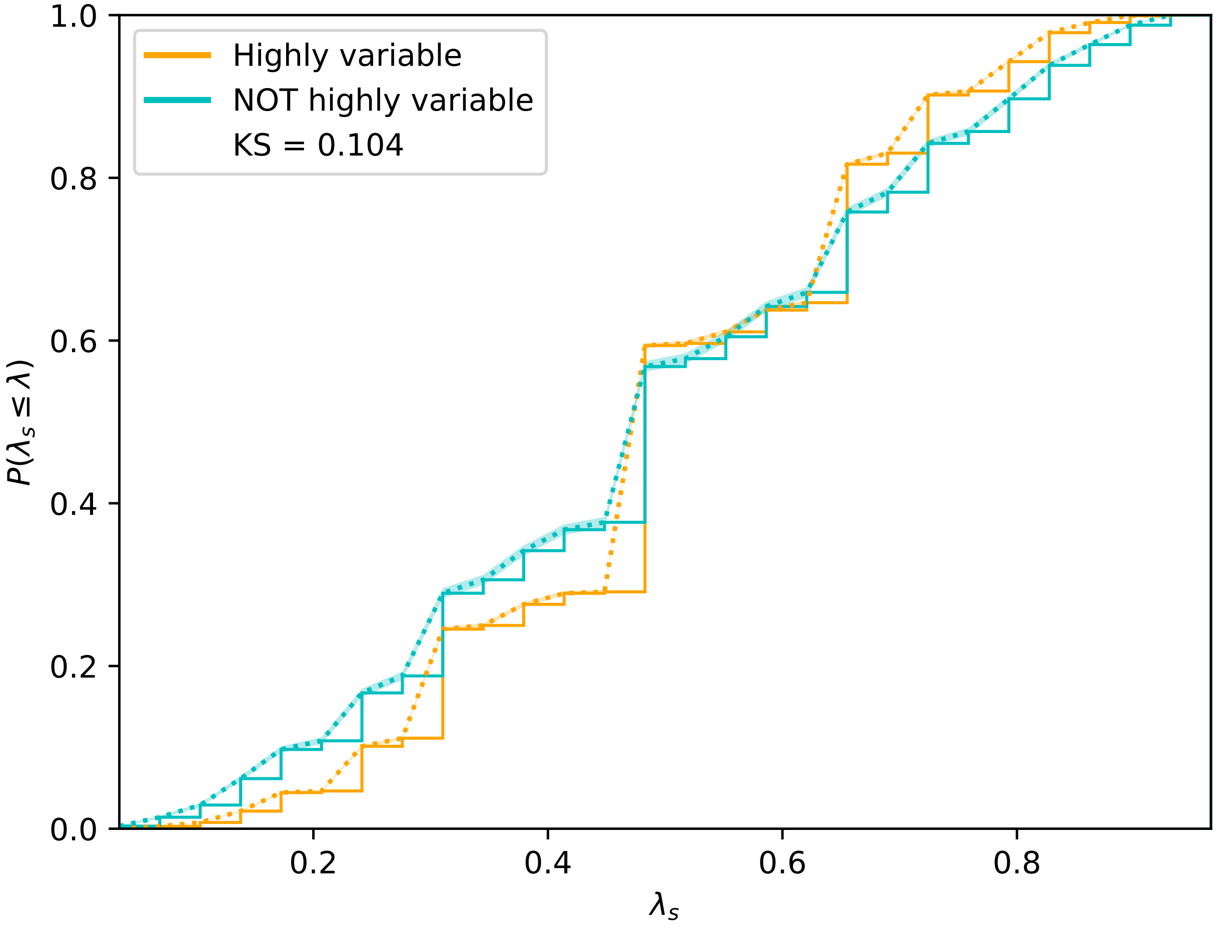

Supplementary Figure 15: Calm mental state.

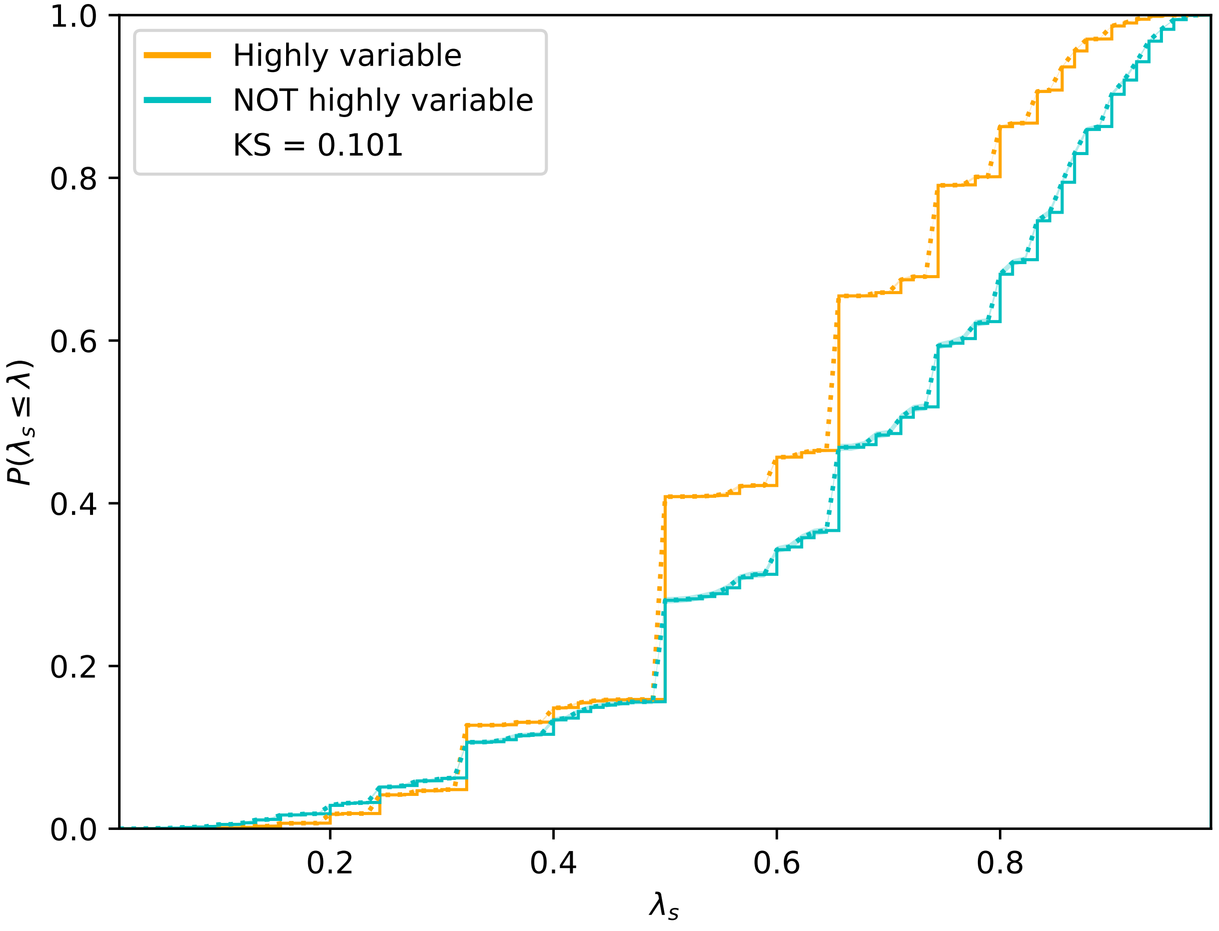

Supplementary Figure 16: Cramps pain experienced.

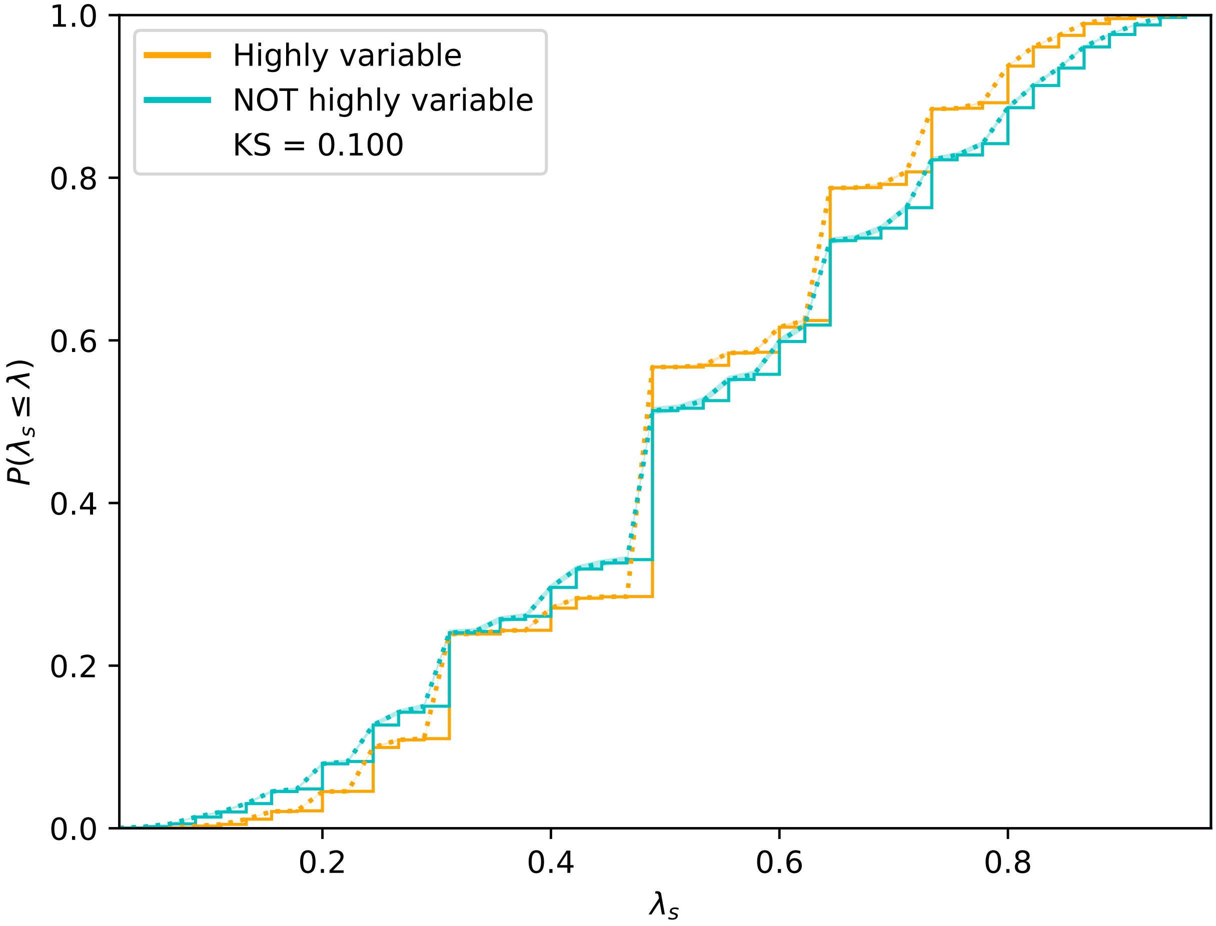

Supplementary Figure 17: 3-6 hours of sleep.

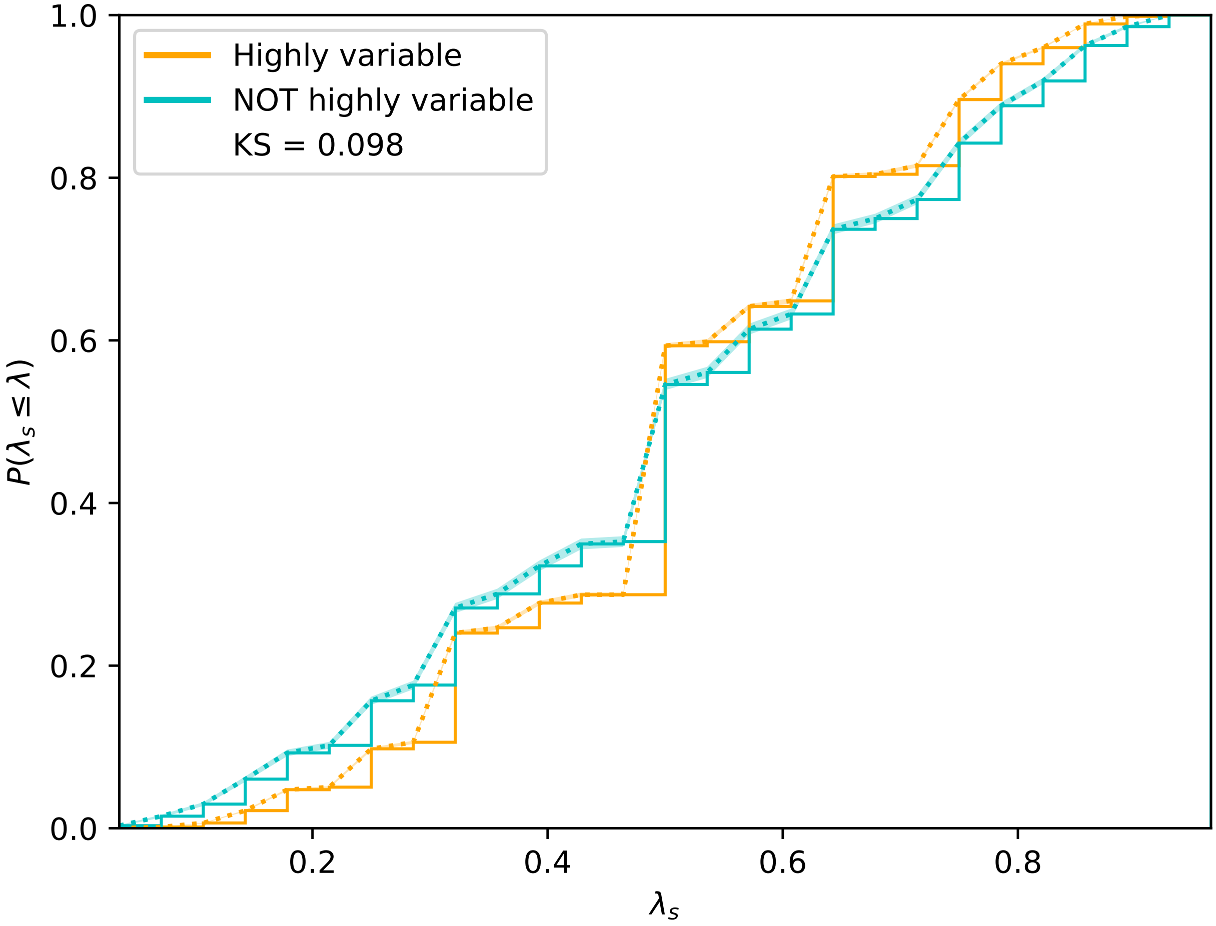

Supplementary Figure 18: Carbs food craving experienced.

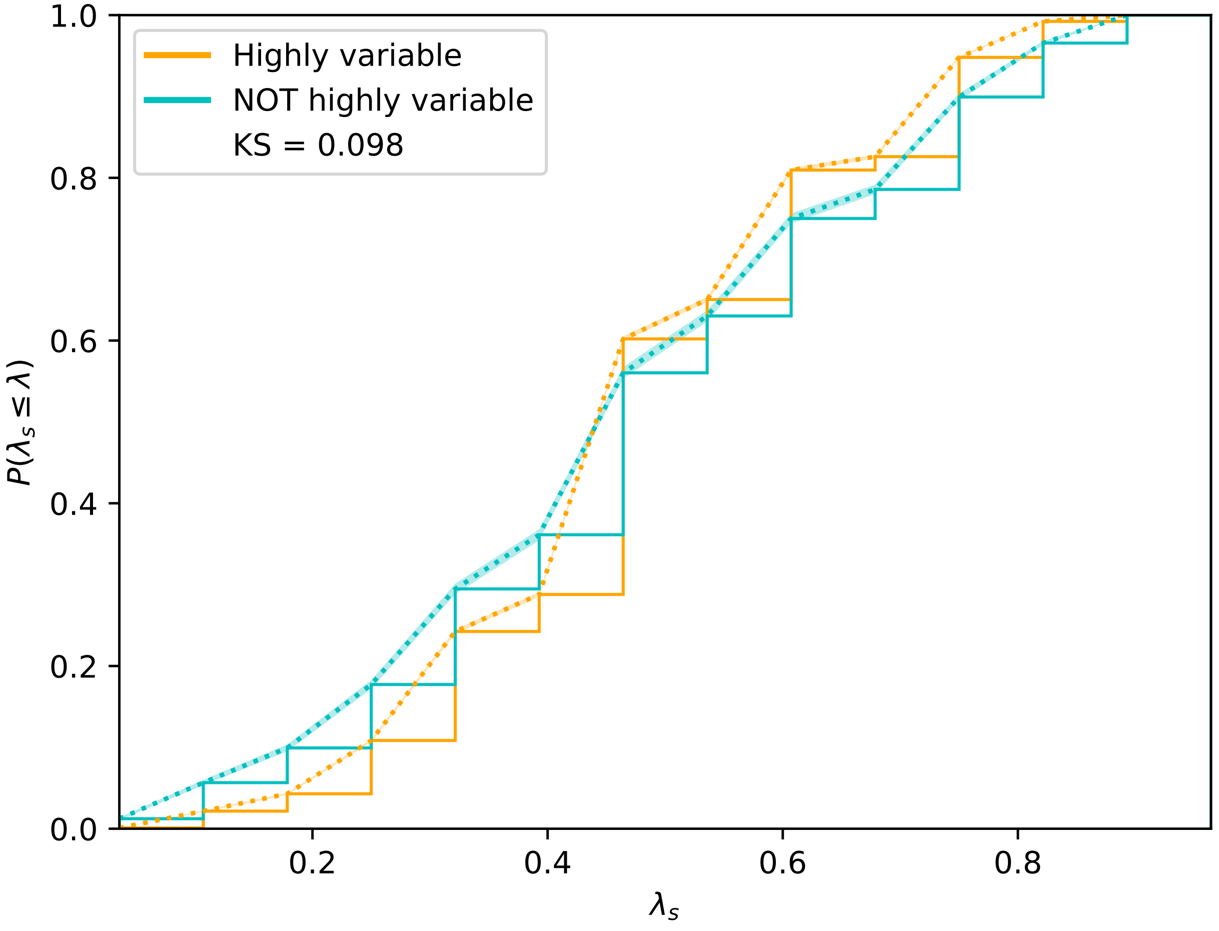

Supplementary Figure 19: Motivated motivation level.

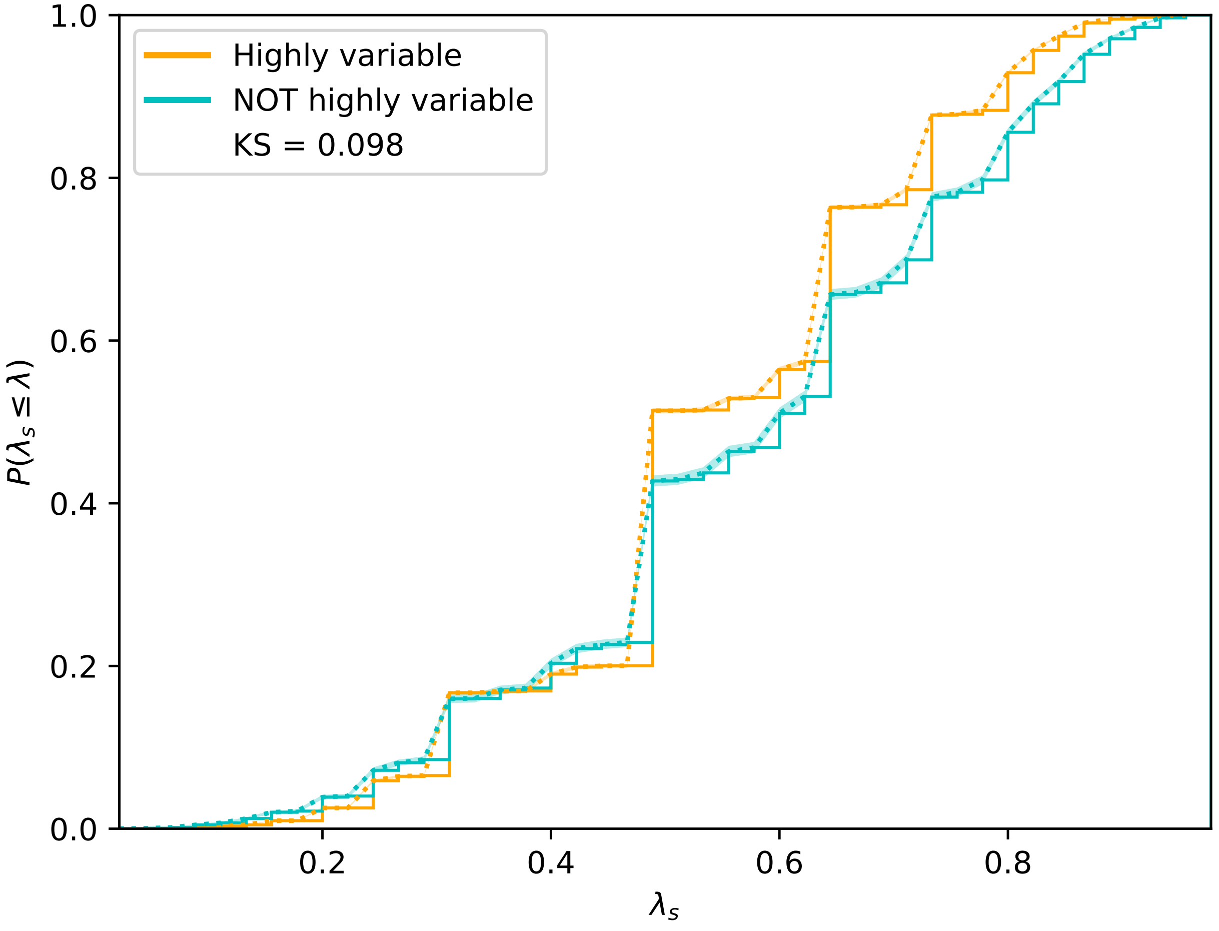

Supplementary Figure 20: Unmotivated motivation level.

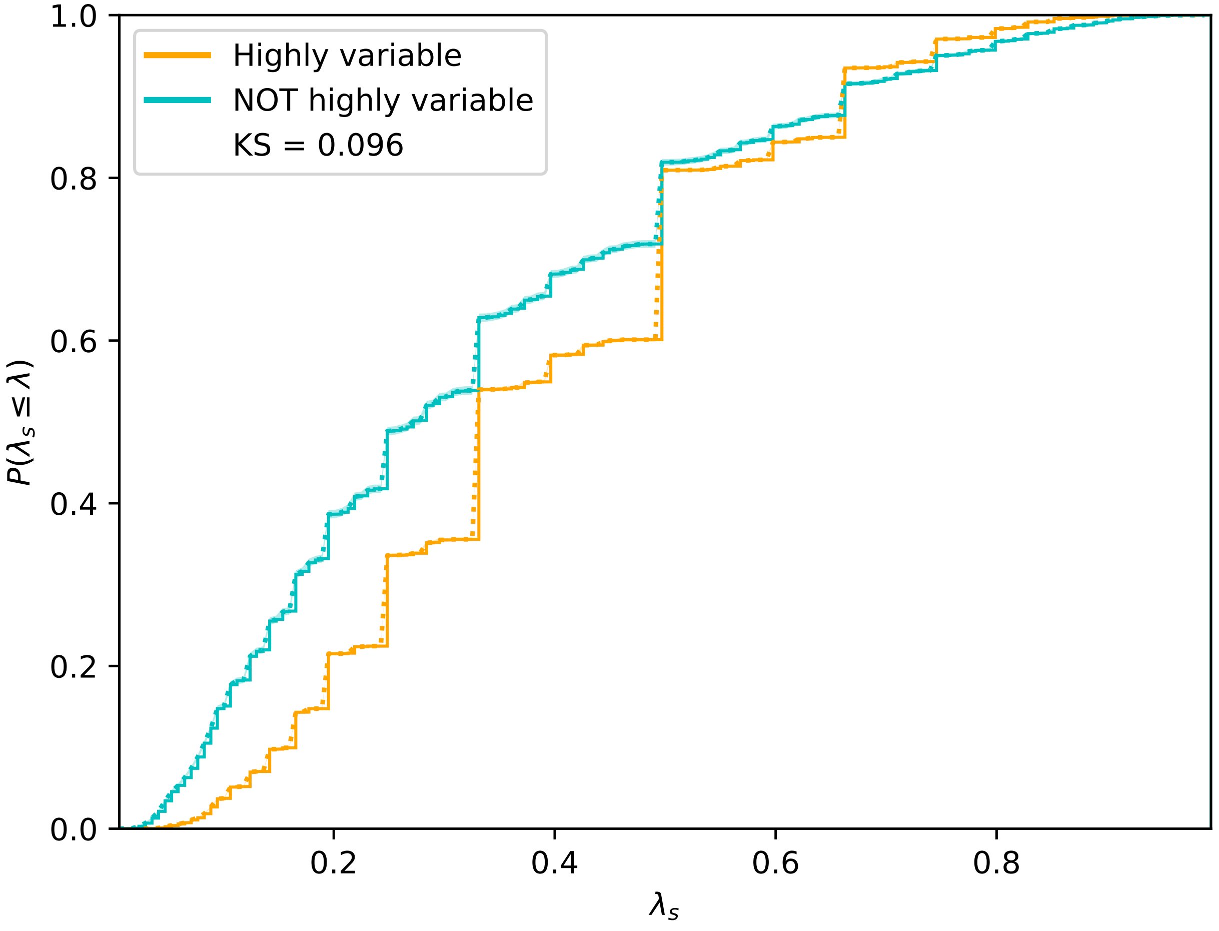

Supplementary Figure 21: Ovulation pain experienced.

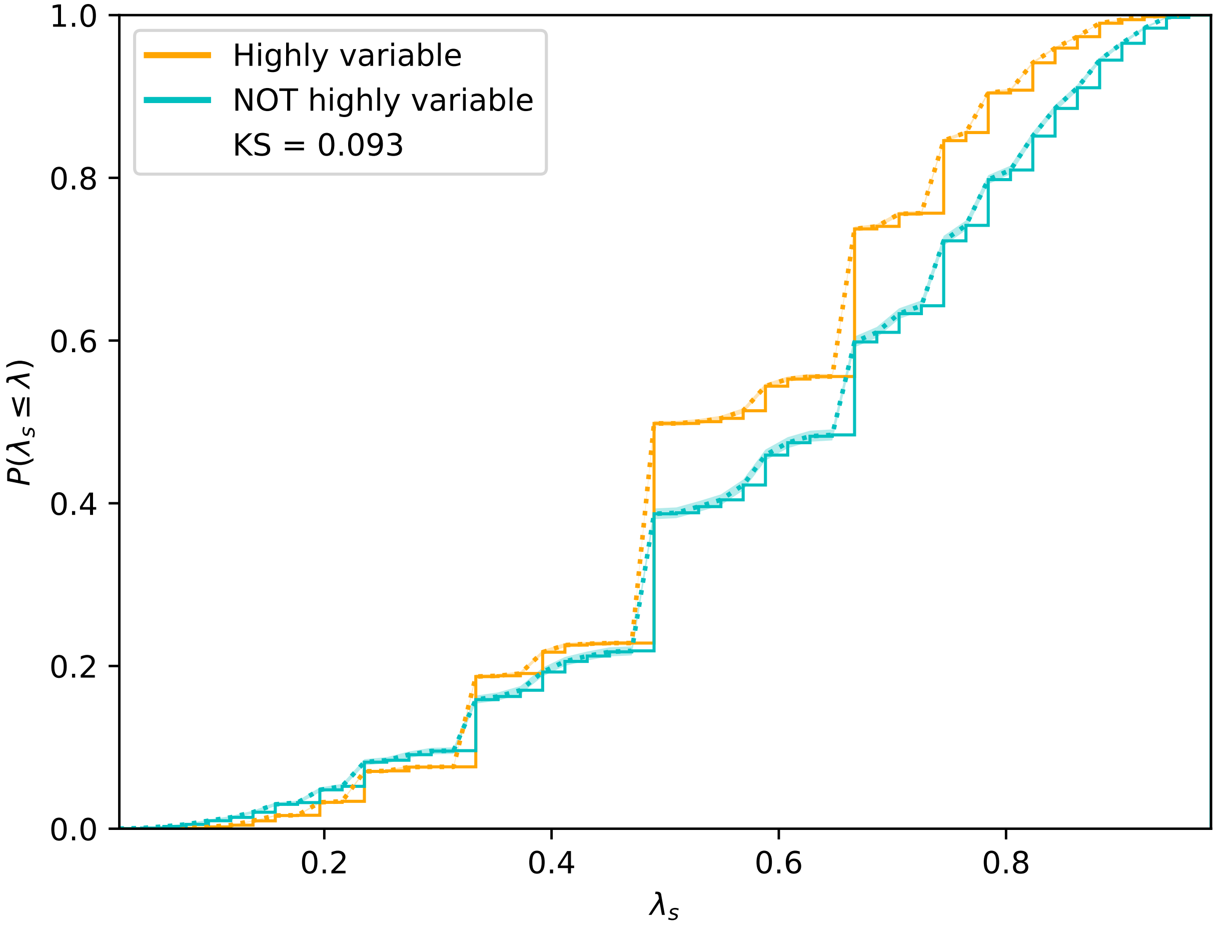

Supplementary Figure 22: Acne skin health reported.

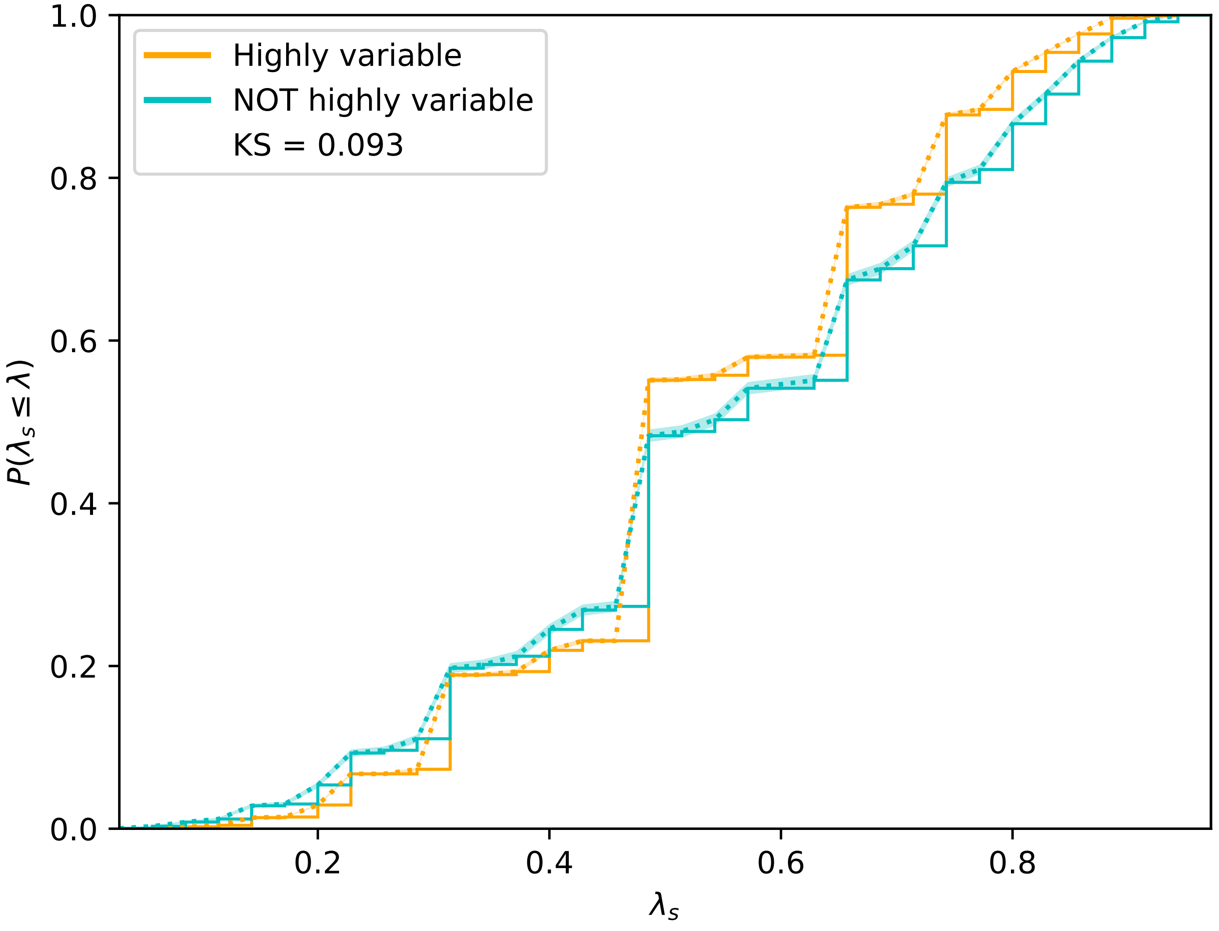

Supplementary Figure 23: Withdrawn social behavior.

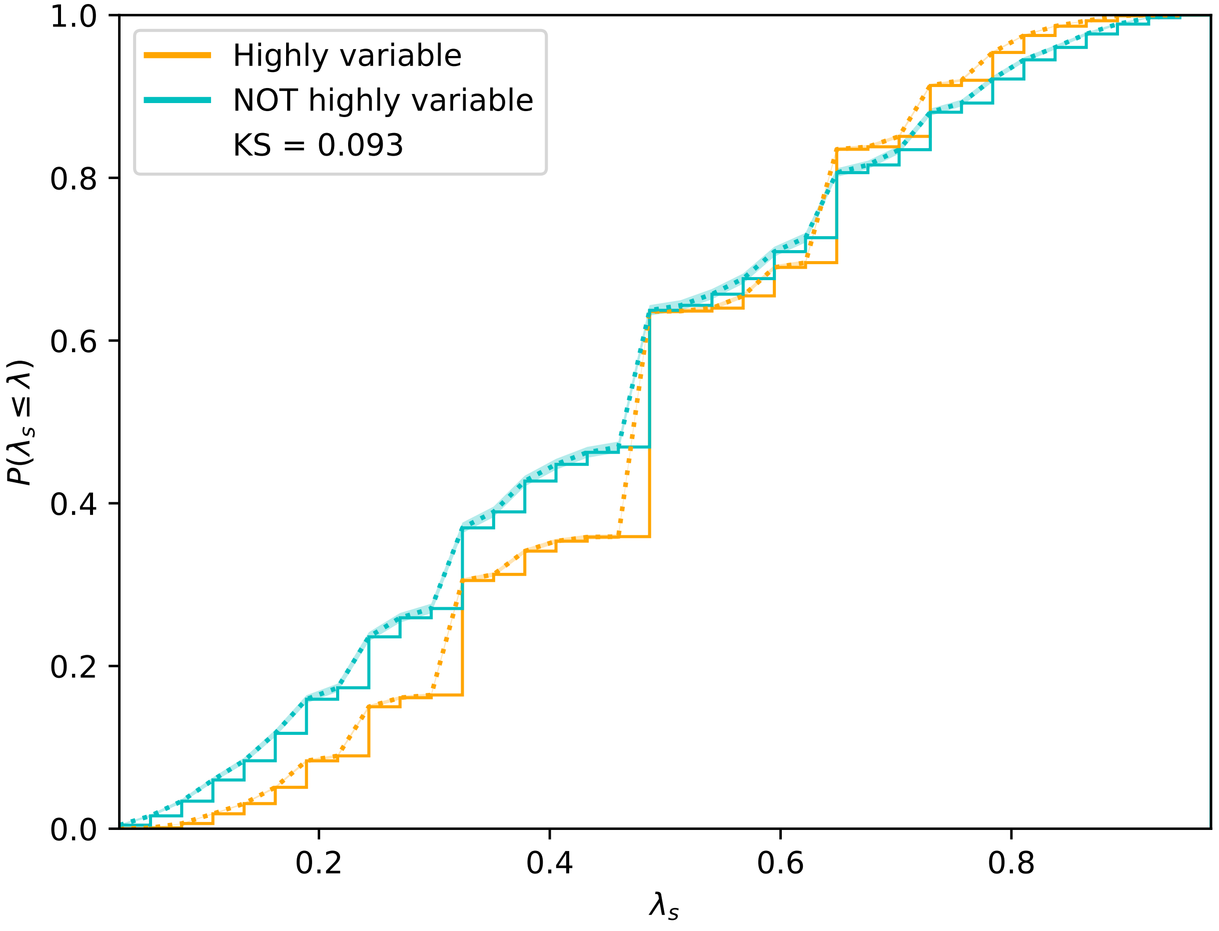

Supplementary Figure 24: Oily skin health reported.

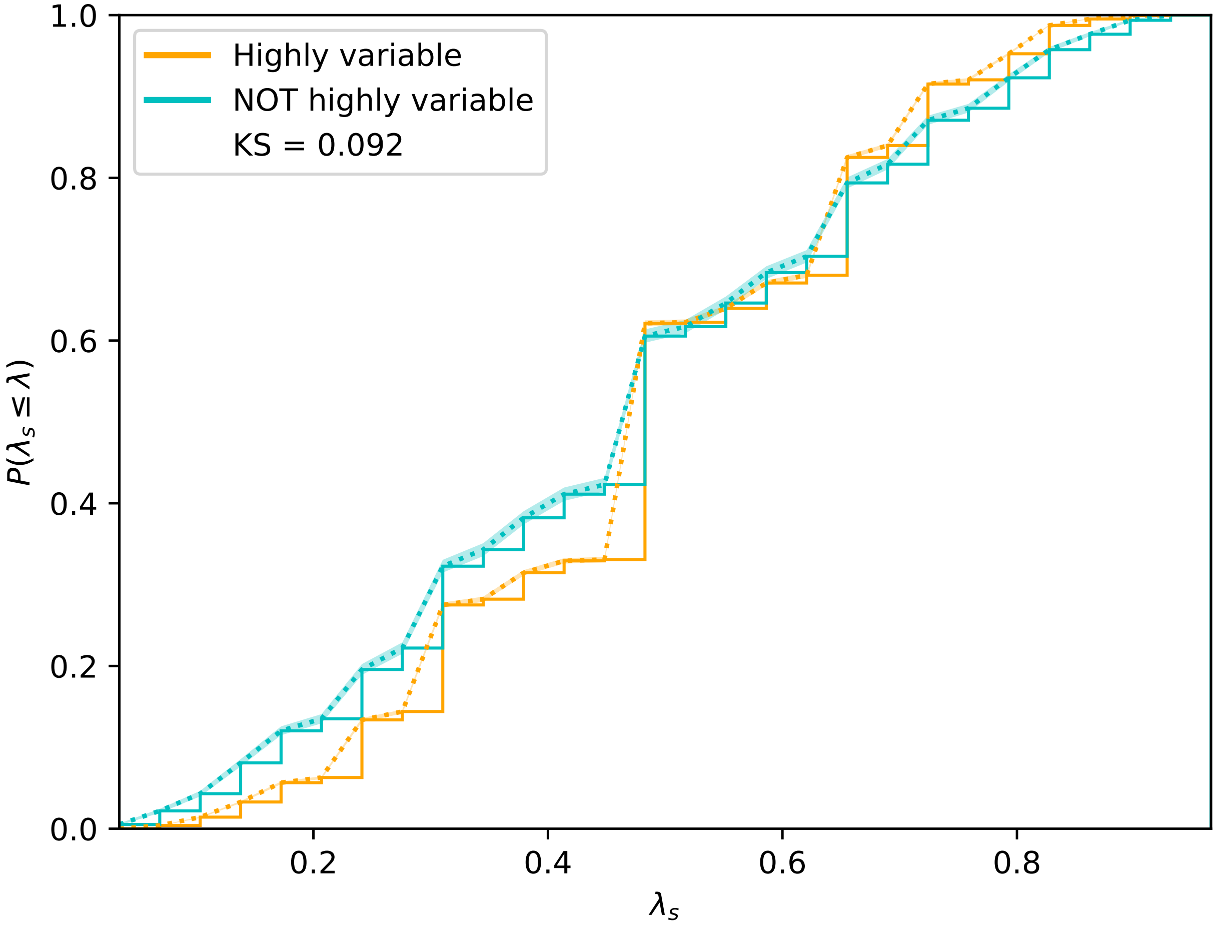

Supplementary Figure 25: Bad hair reported.

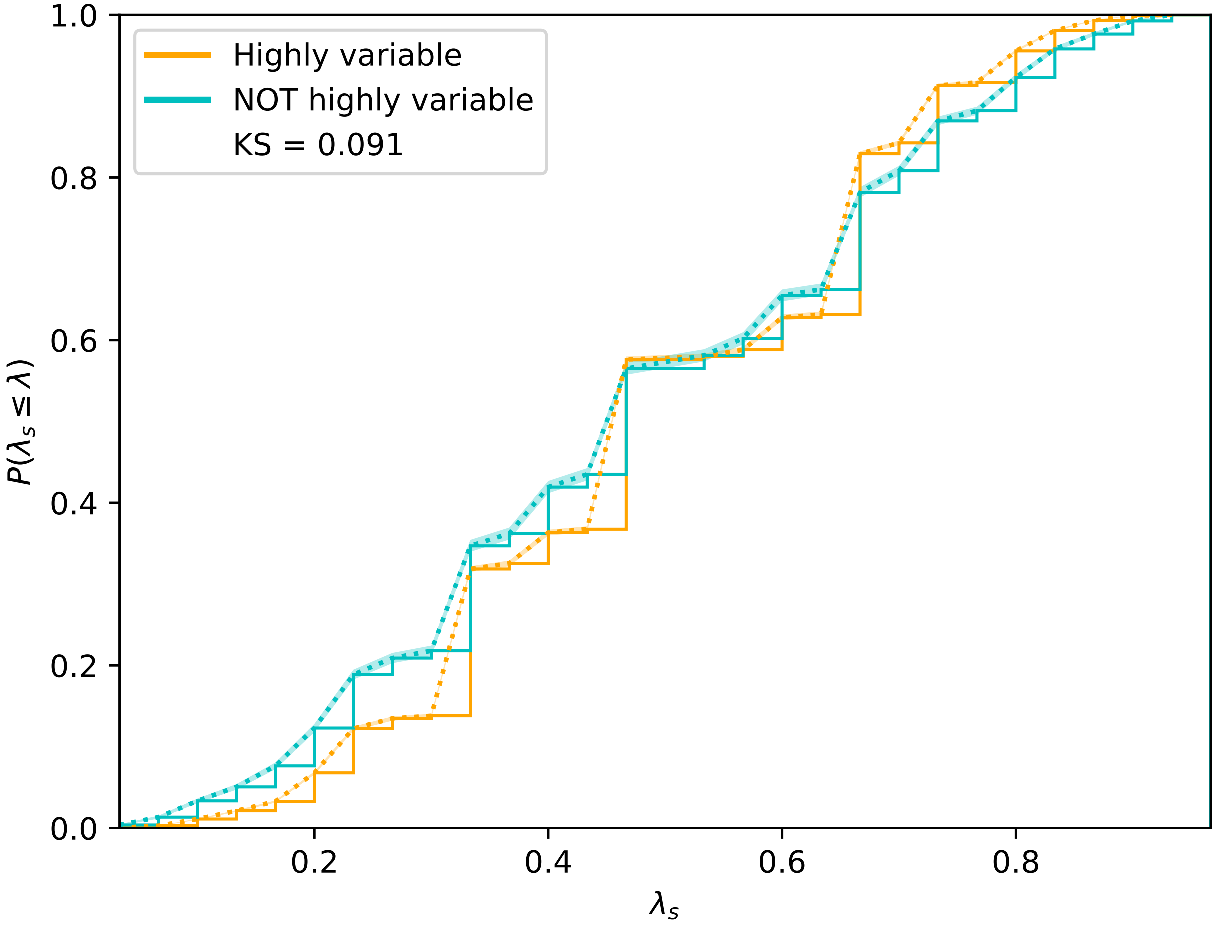

Supplementary Figure 26: Creamy vaginal discharge type.

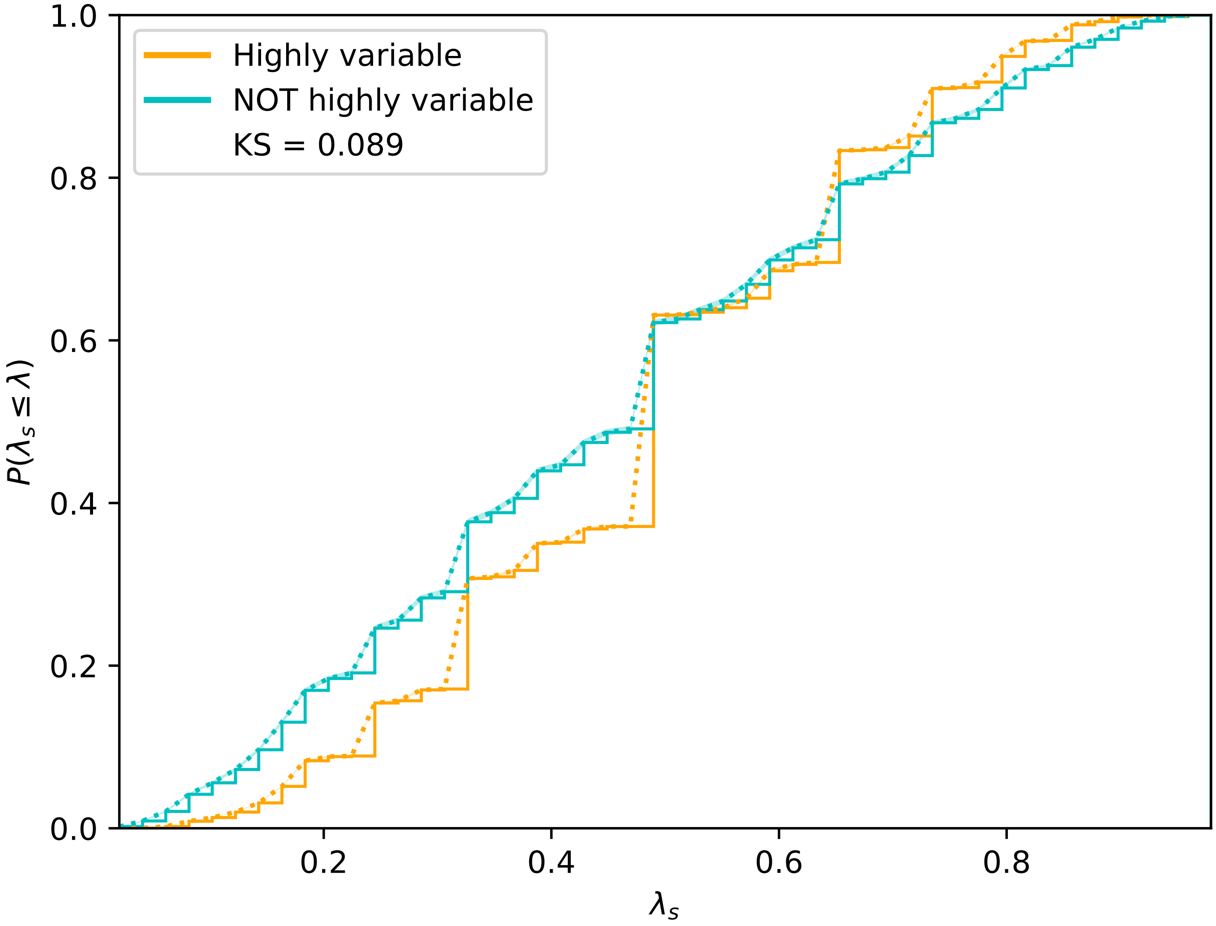

Supplementary Figure 27: Headache pain experienced.

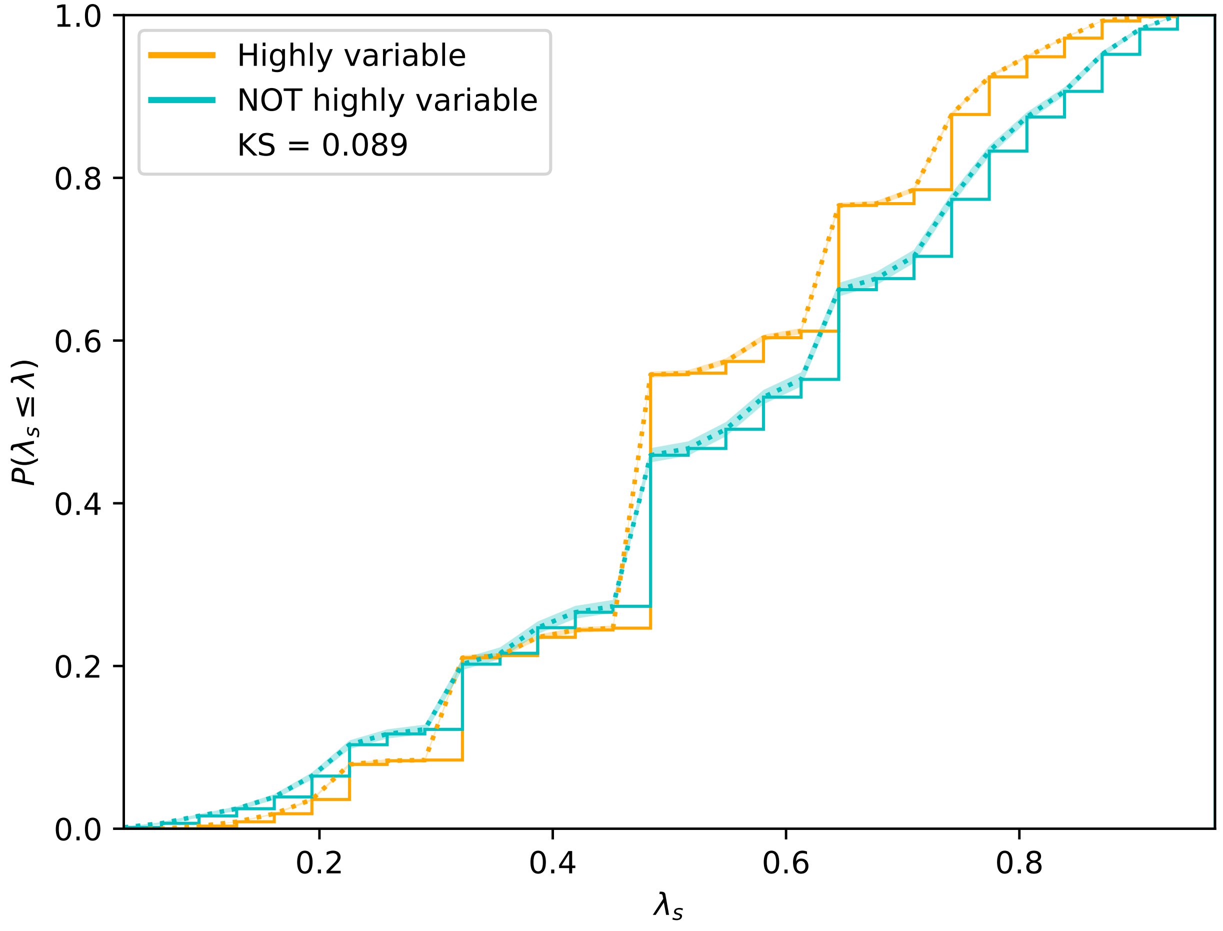

Supplementary Figure 28: Good hair reported.

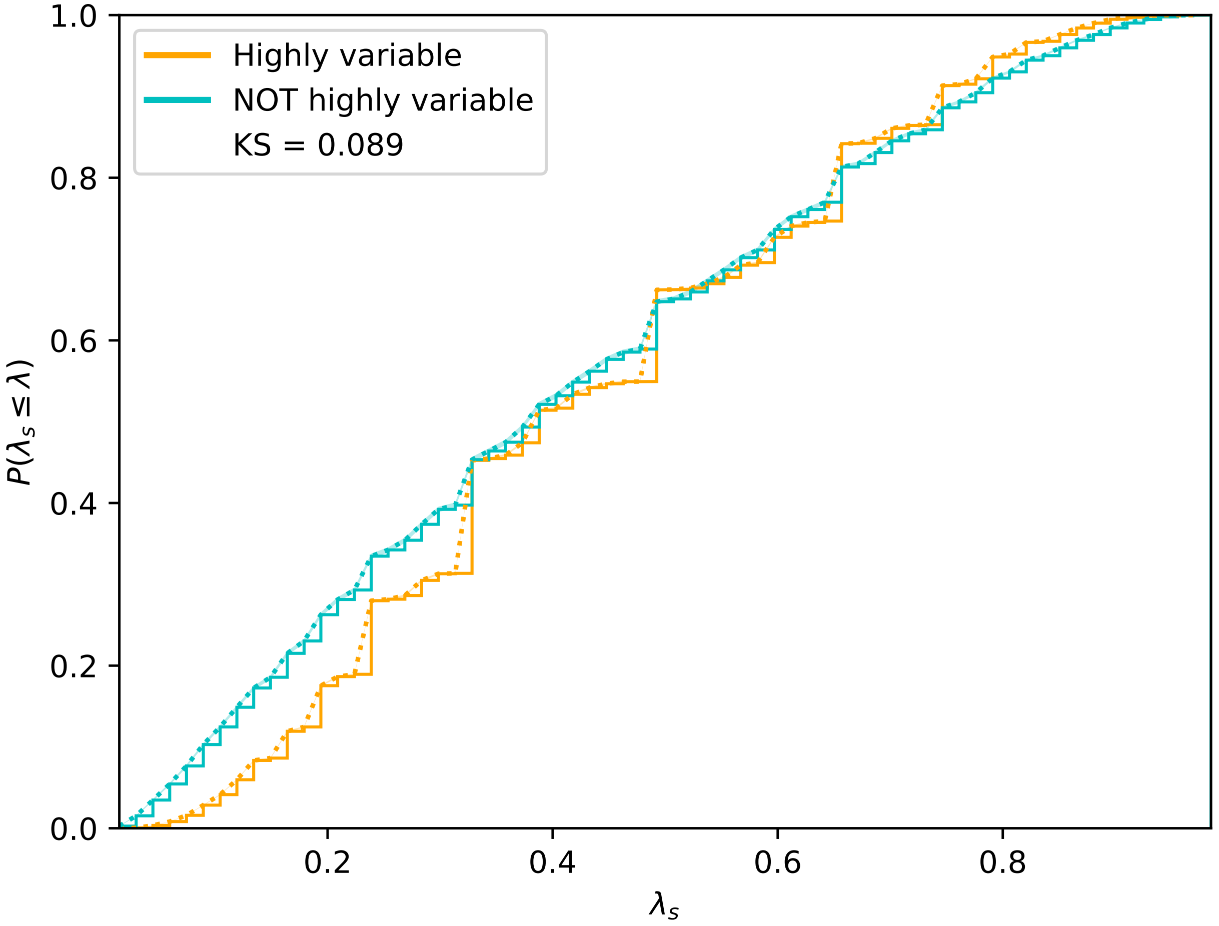

Supplementary Figure 29: Spotting period flow.

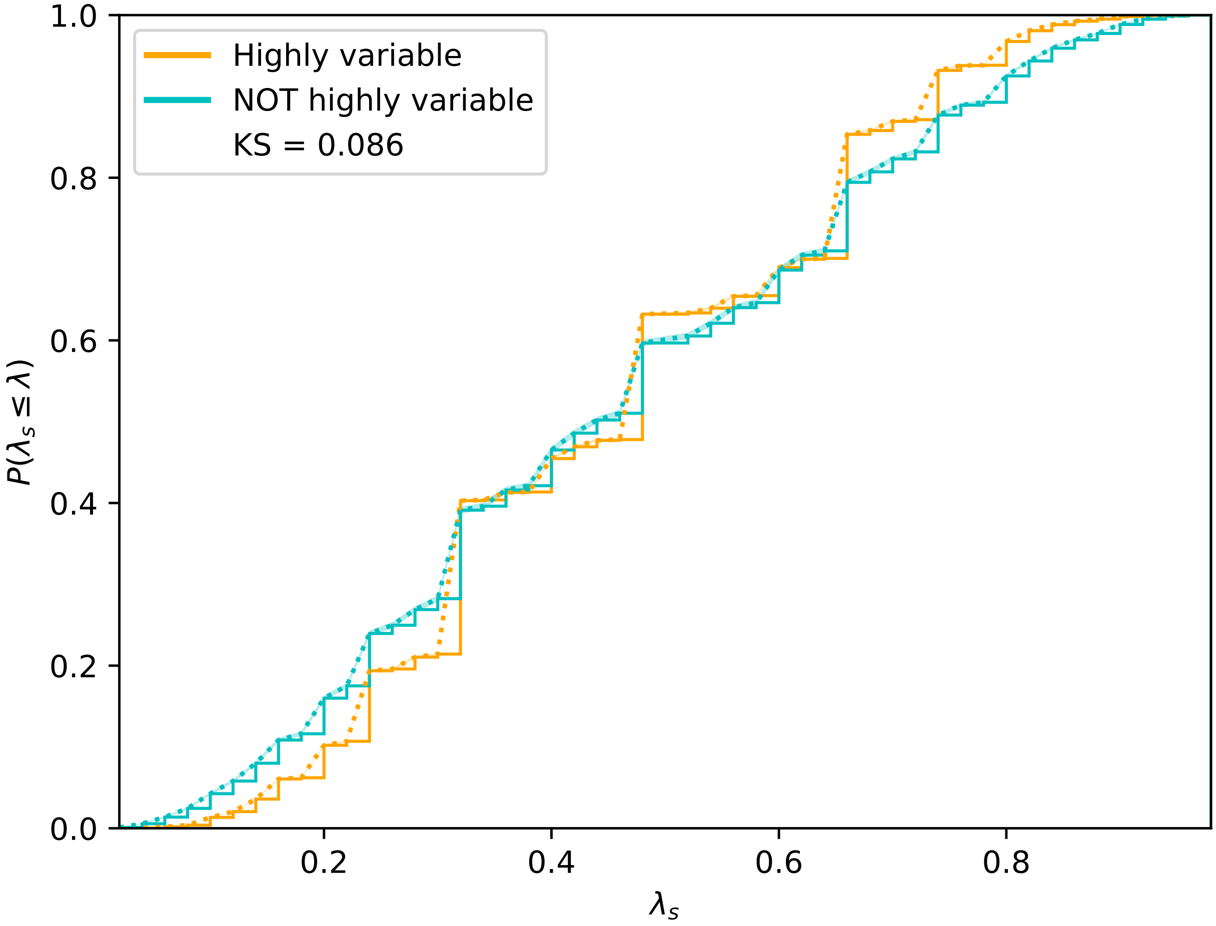

Supplementary Figure 30: PMS emotional state.

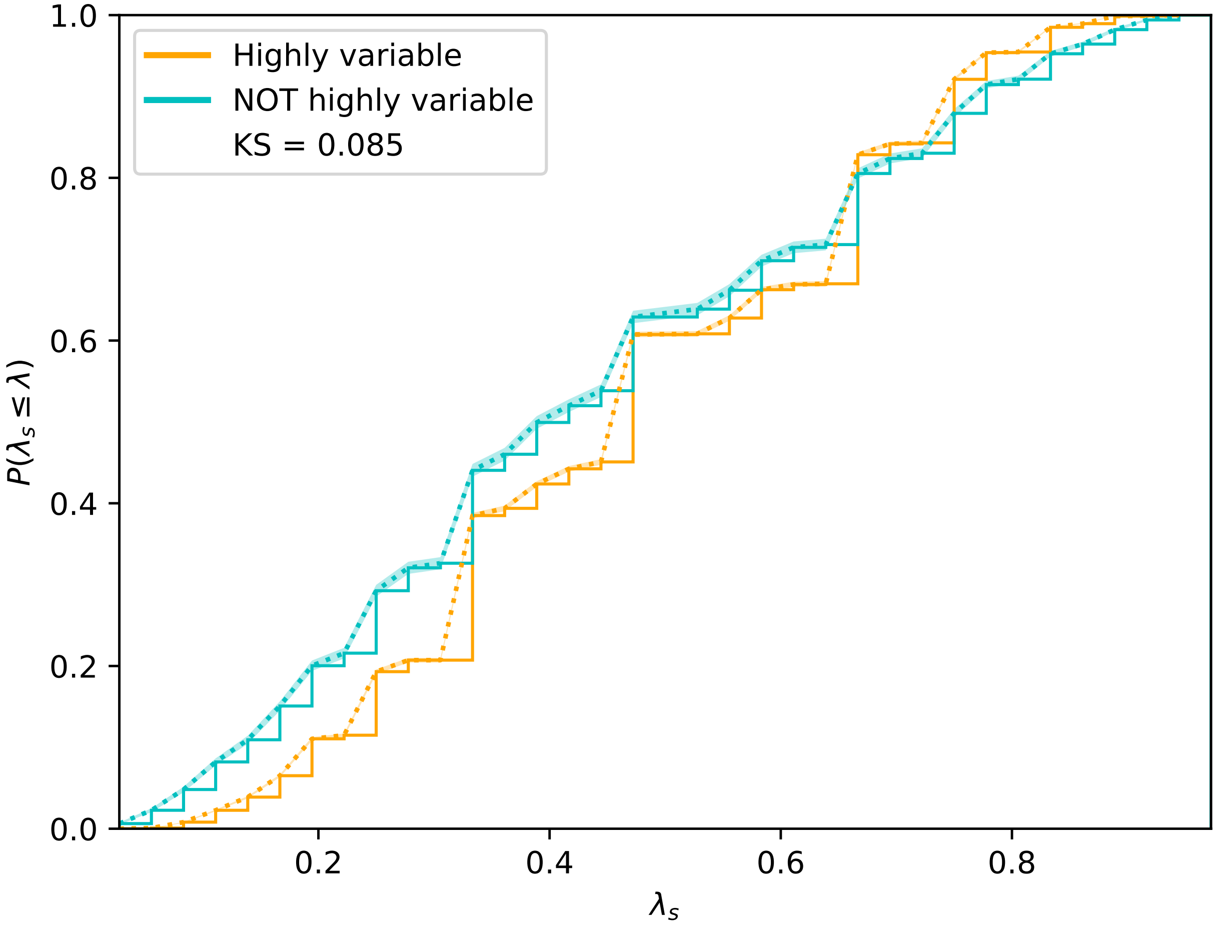

Supplementary Figure 31: Great digestive health.

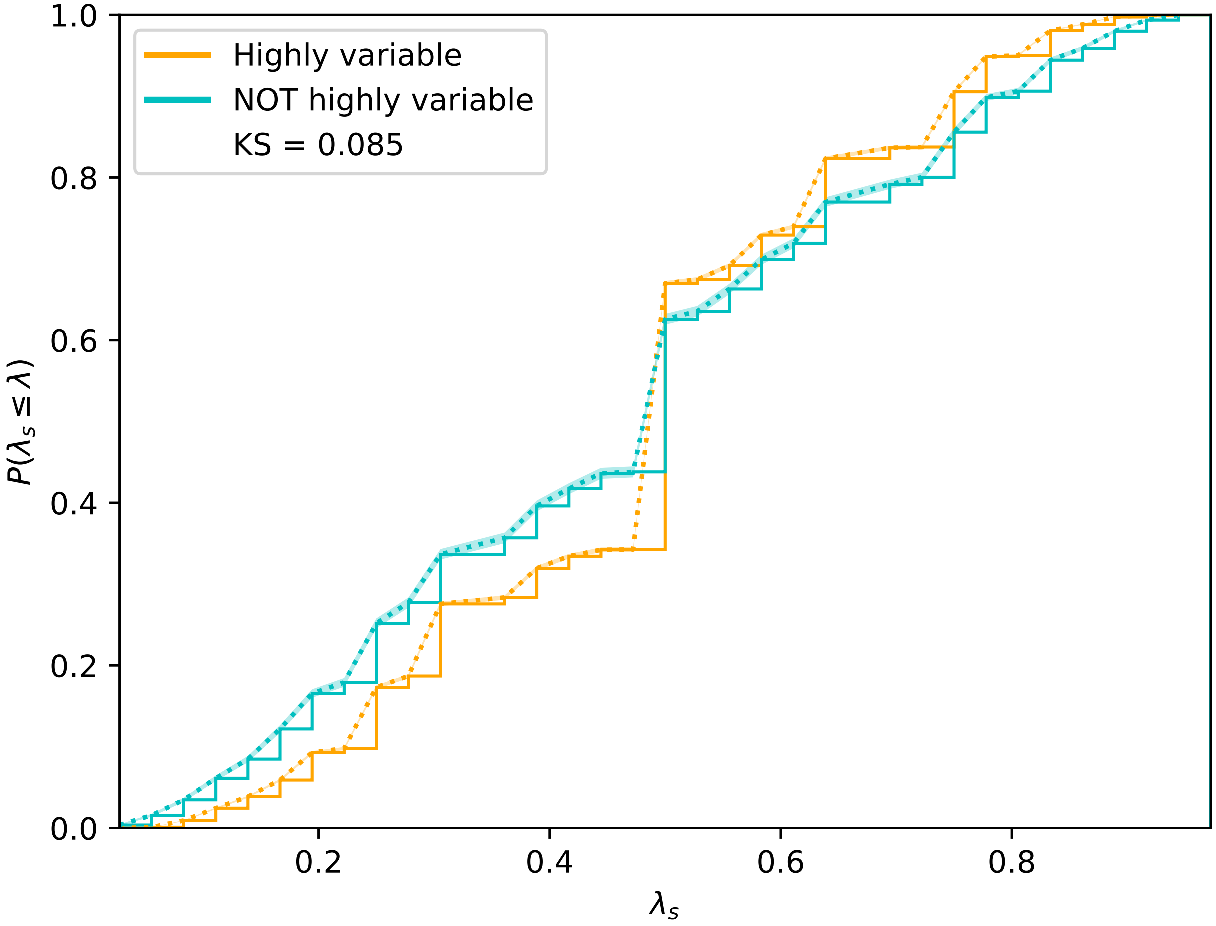

Supplementary Figure 32: Good skin health reported.

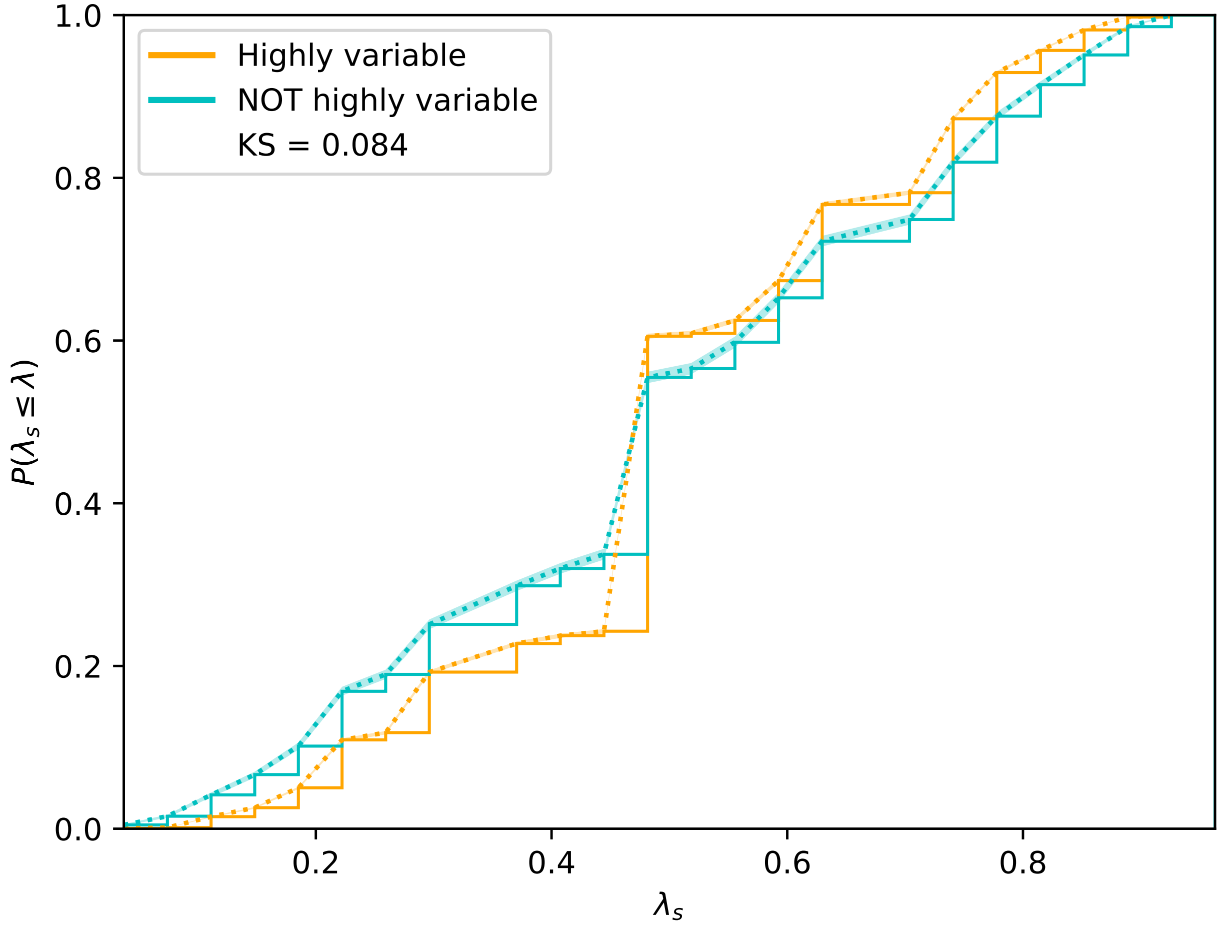

Supplementary Figure 33: Salty food craving experienced.

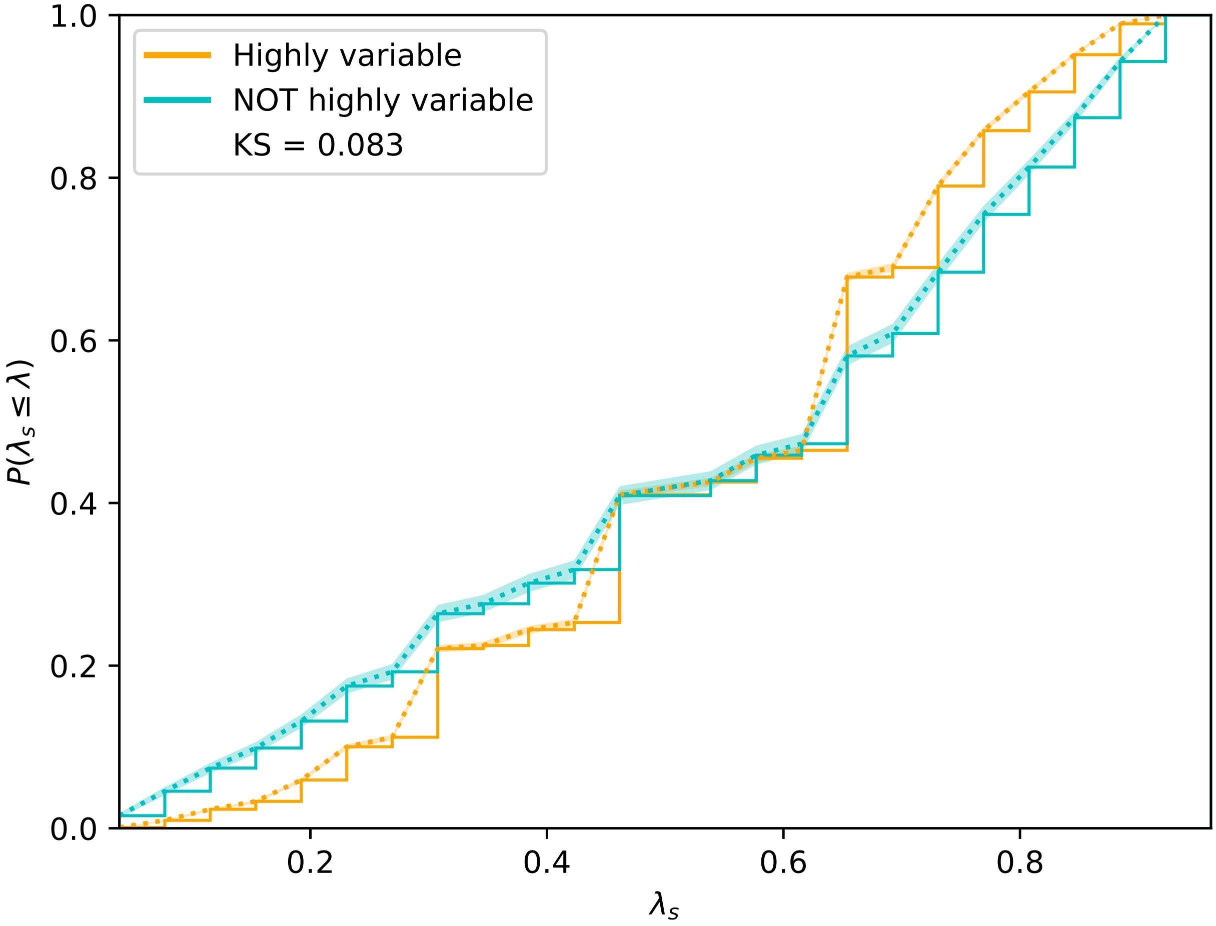

Supplementary Figure 34: Pad method used for period collection.

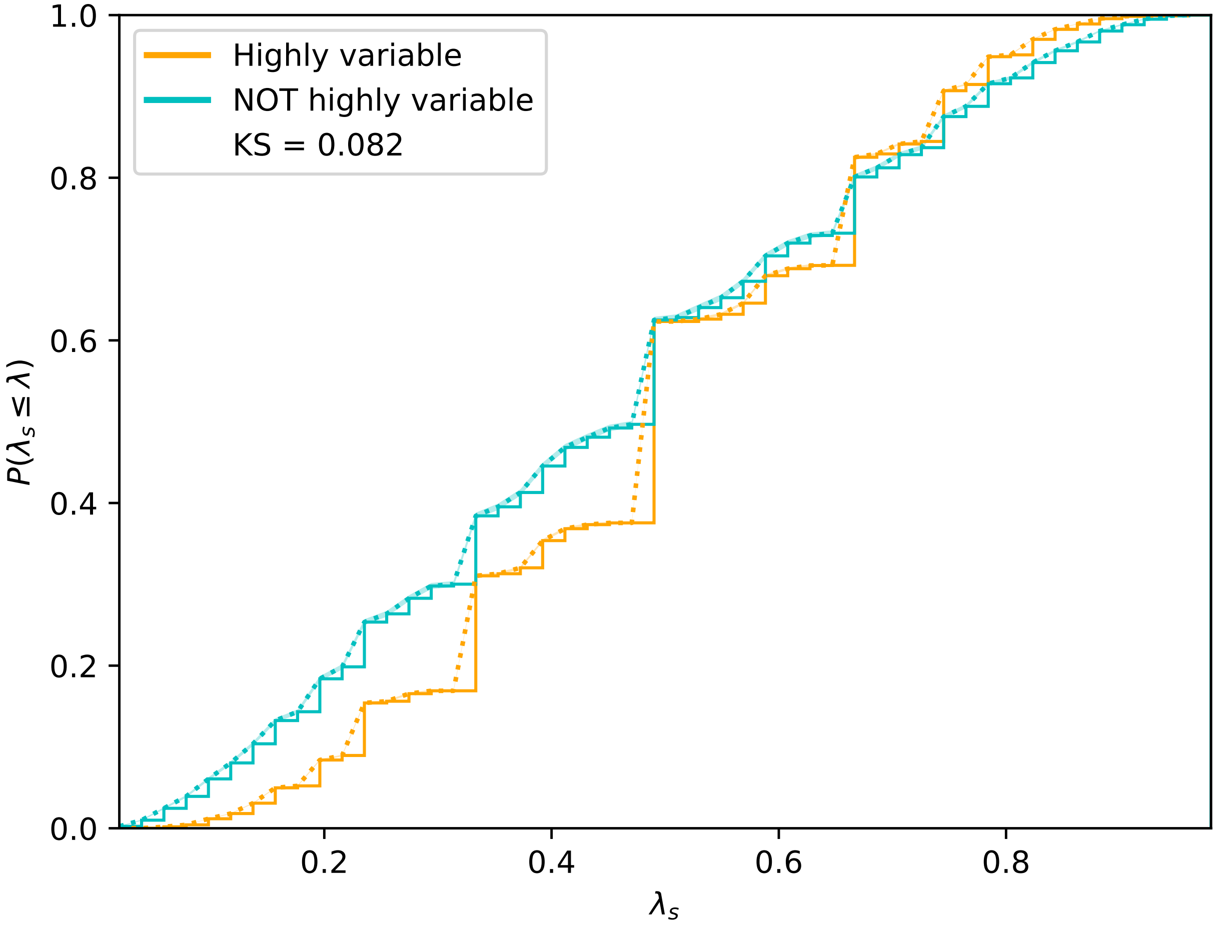

Supplementary Figure 35: Tender breasts pain experienced.

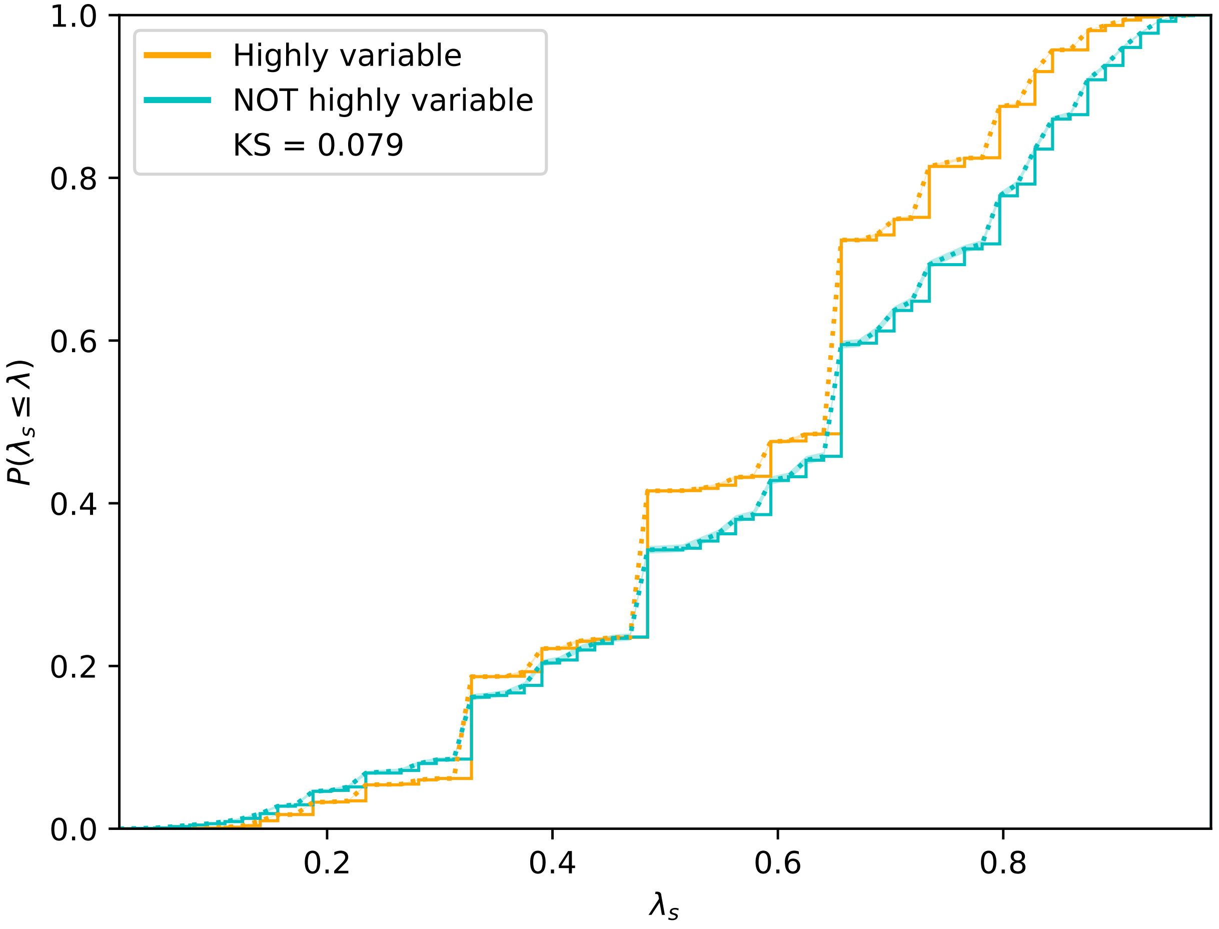

Supplementary Figure 36: 6-9 hours of sleep.

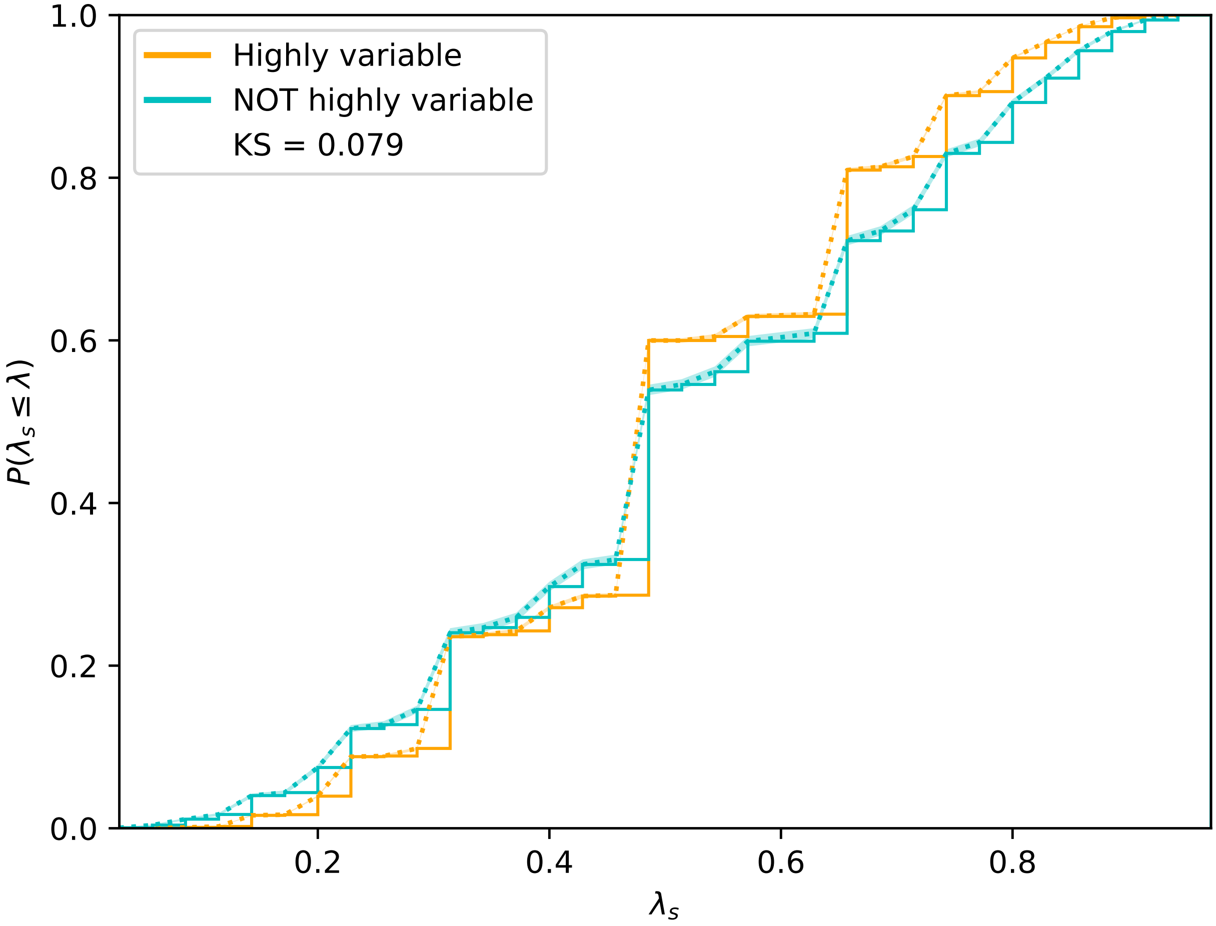

Supplementary Figure 37: Stressed mental state.

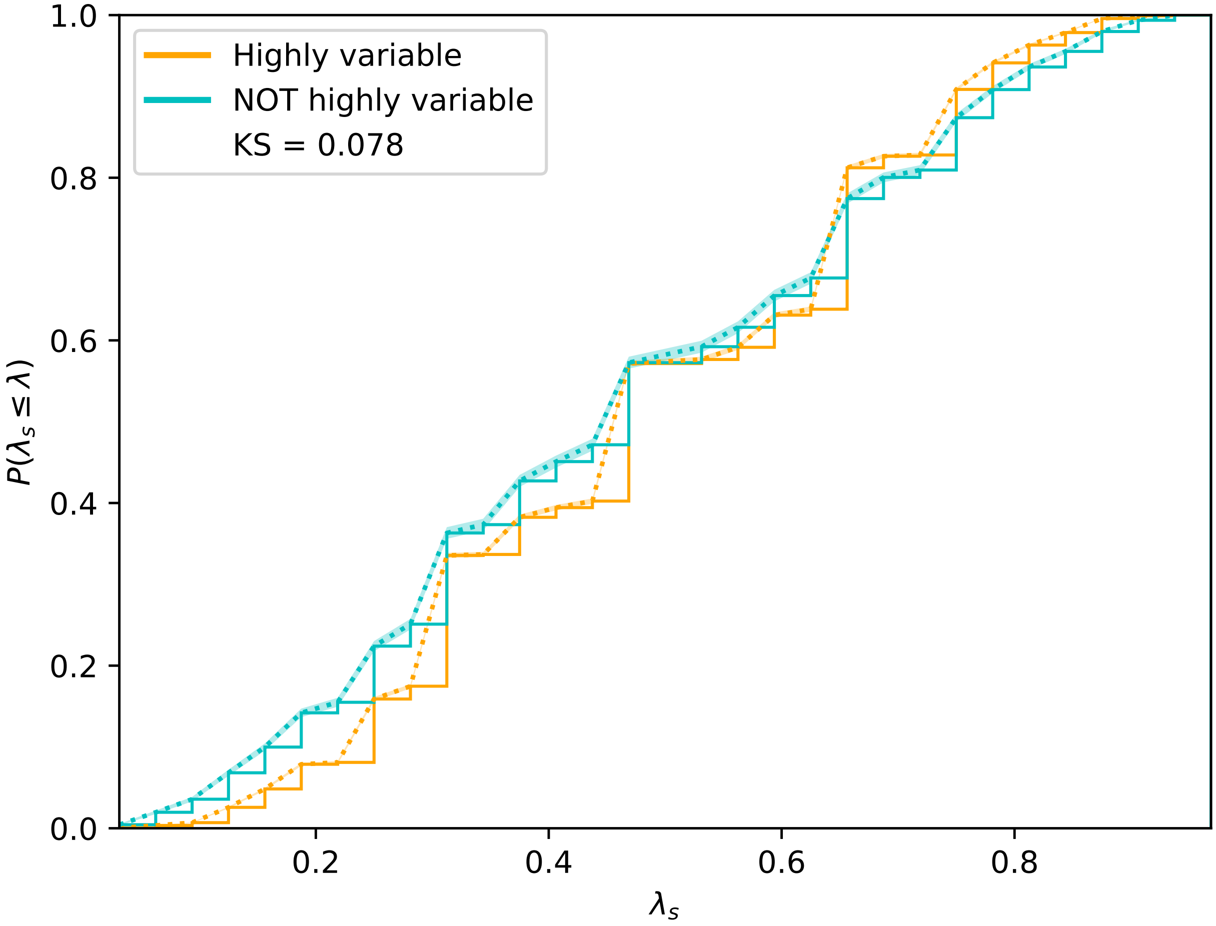

Supplementary Figure 38: Constipated stool health.

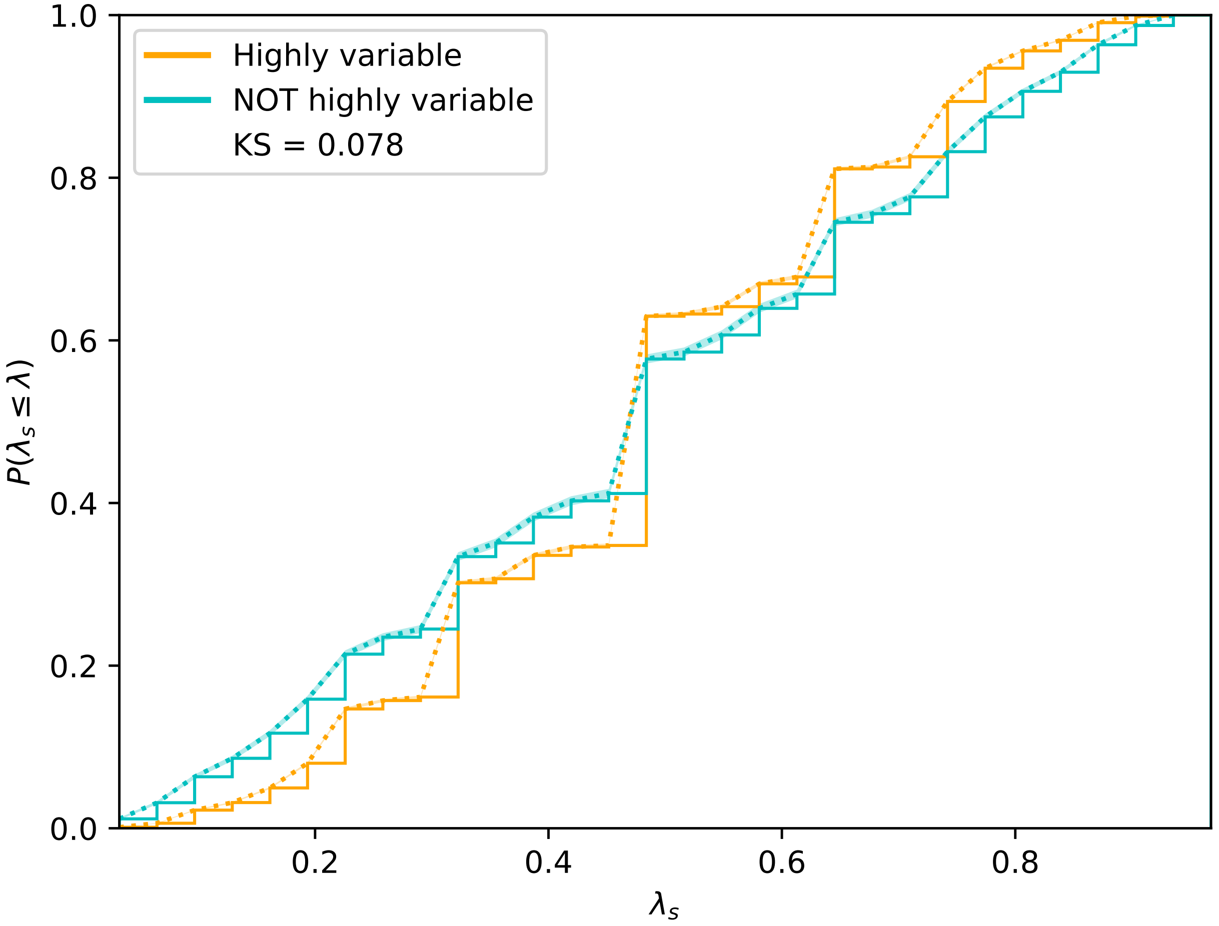

Supplementary Figure 39: Unprotected sex reported.

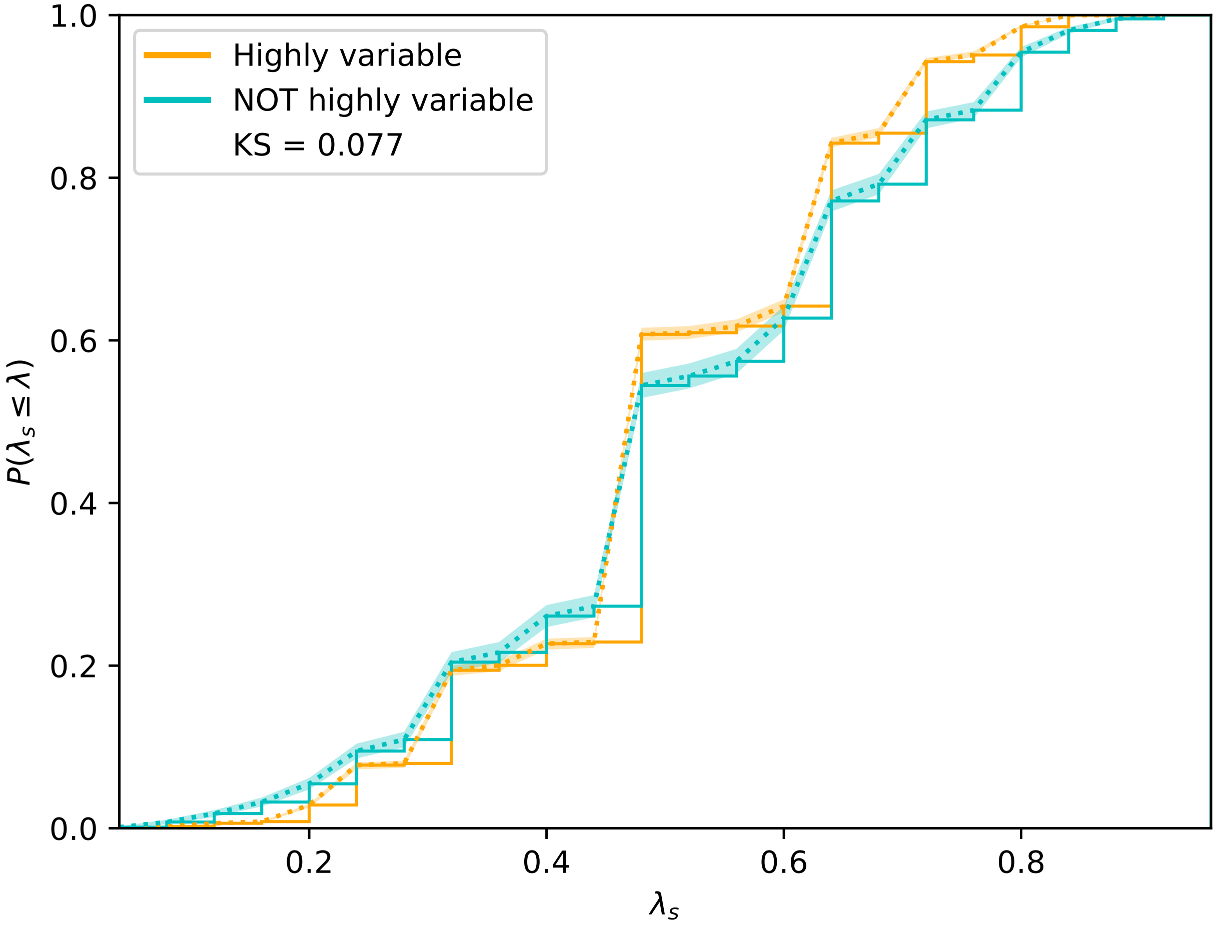

Supplementary Figure 40: Physical maladies: cold/flu.

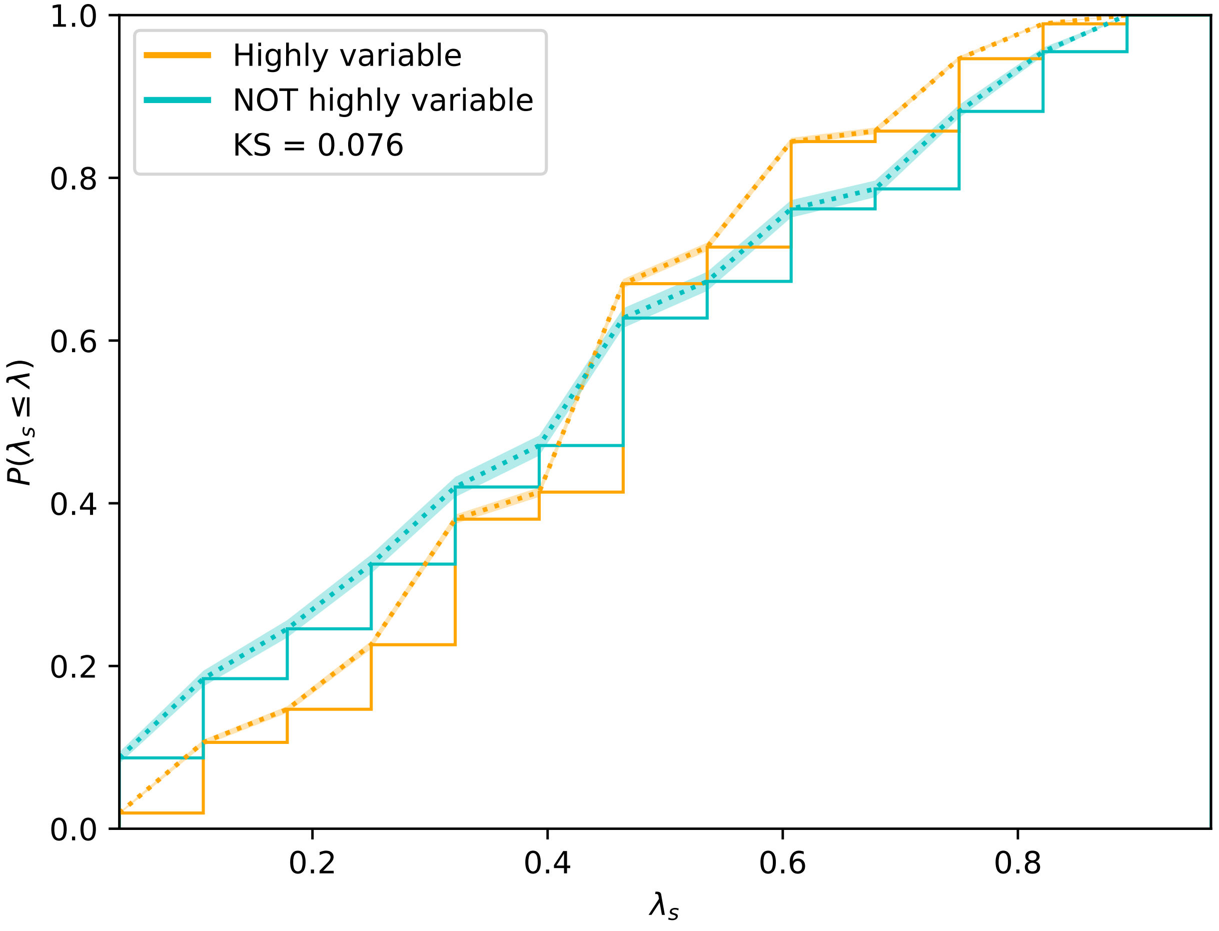

Supplementary Figure 41: Tampon method used for period collection.

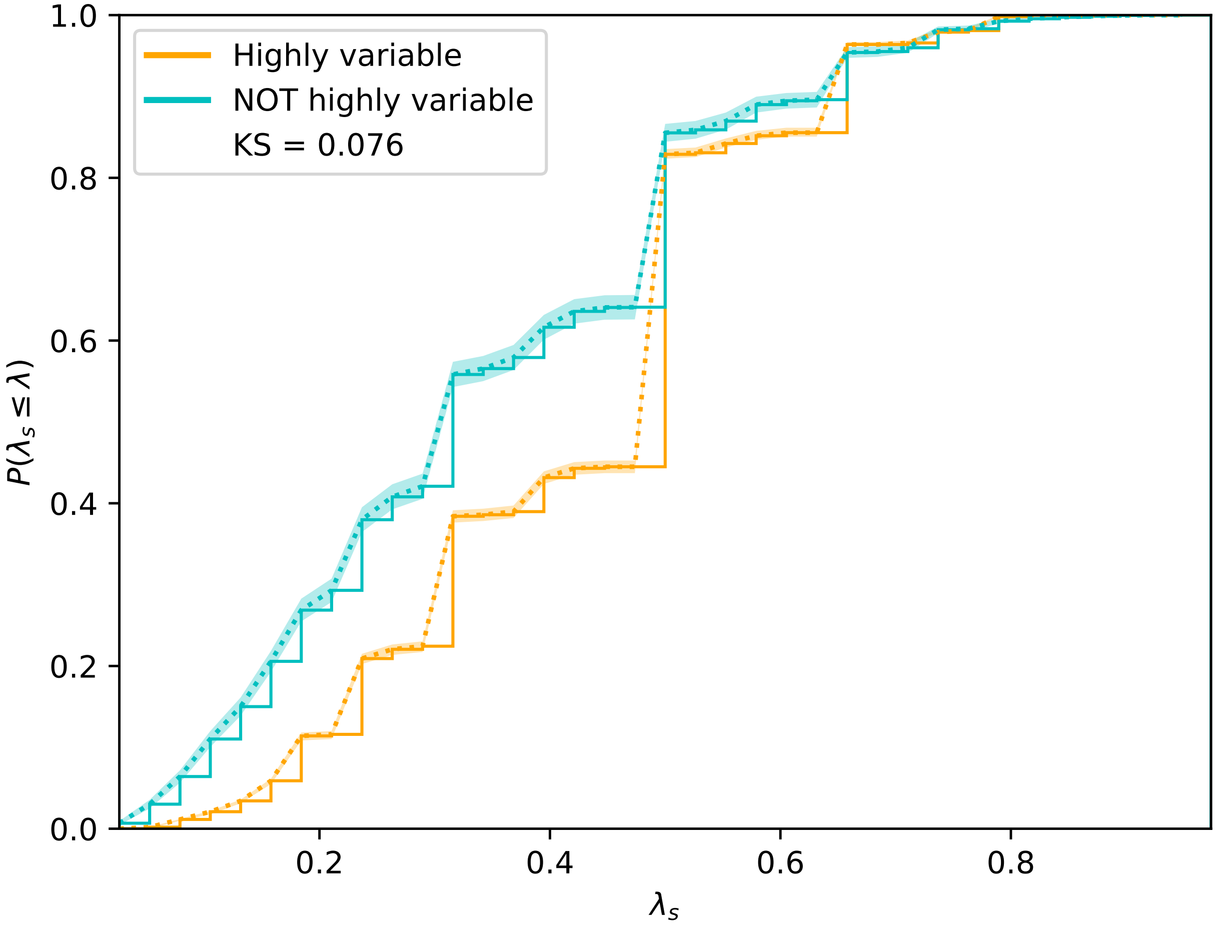

Supplementary Figure 42: Cold/flu medication taken.

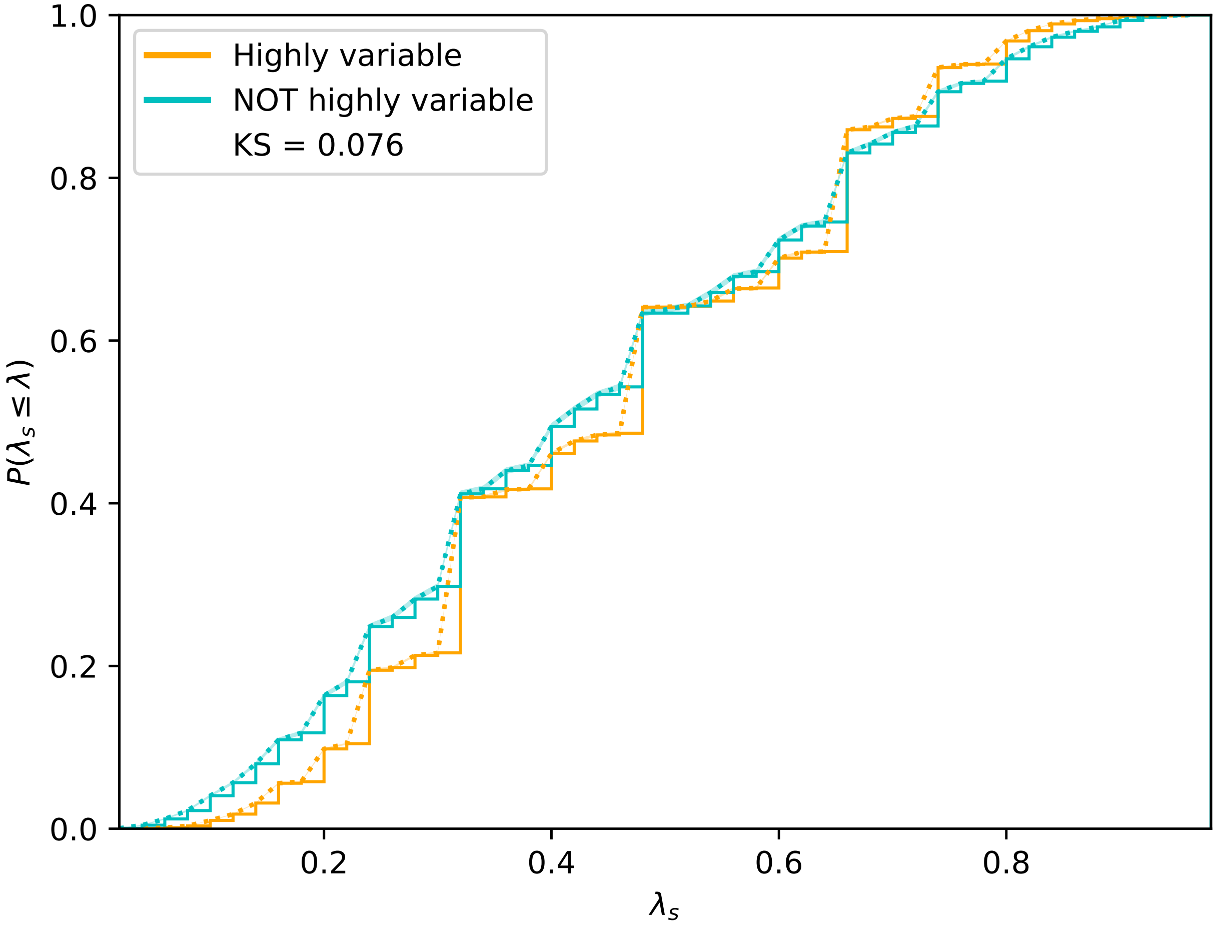

Supplementary Figure 43: Sad emotional state.

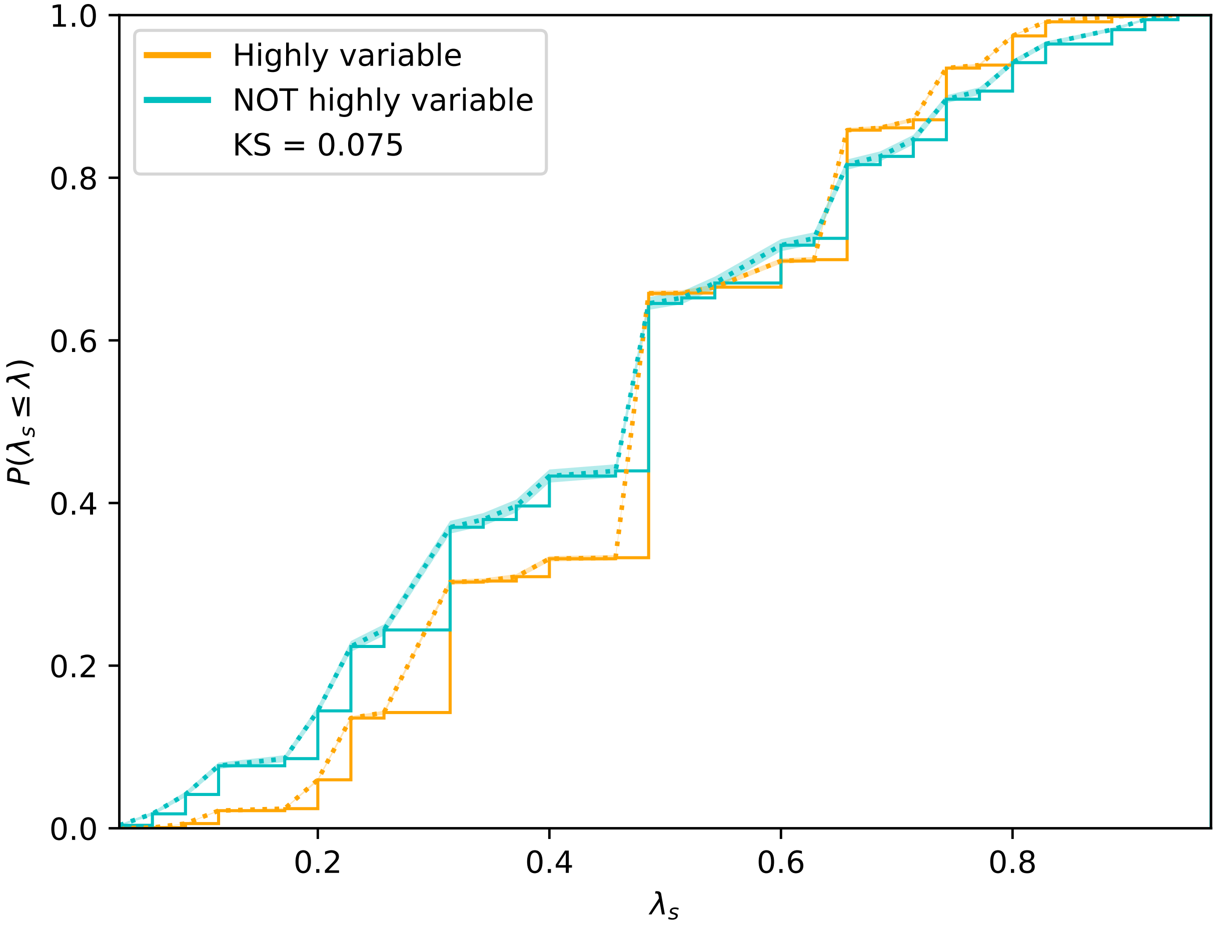

Supplementary Figure 44: Supportive social behavior.

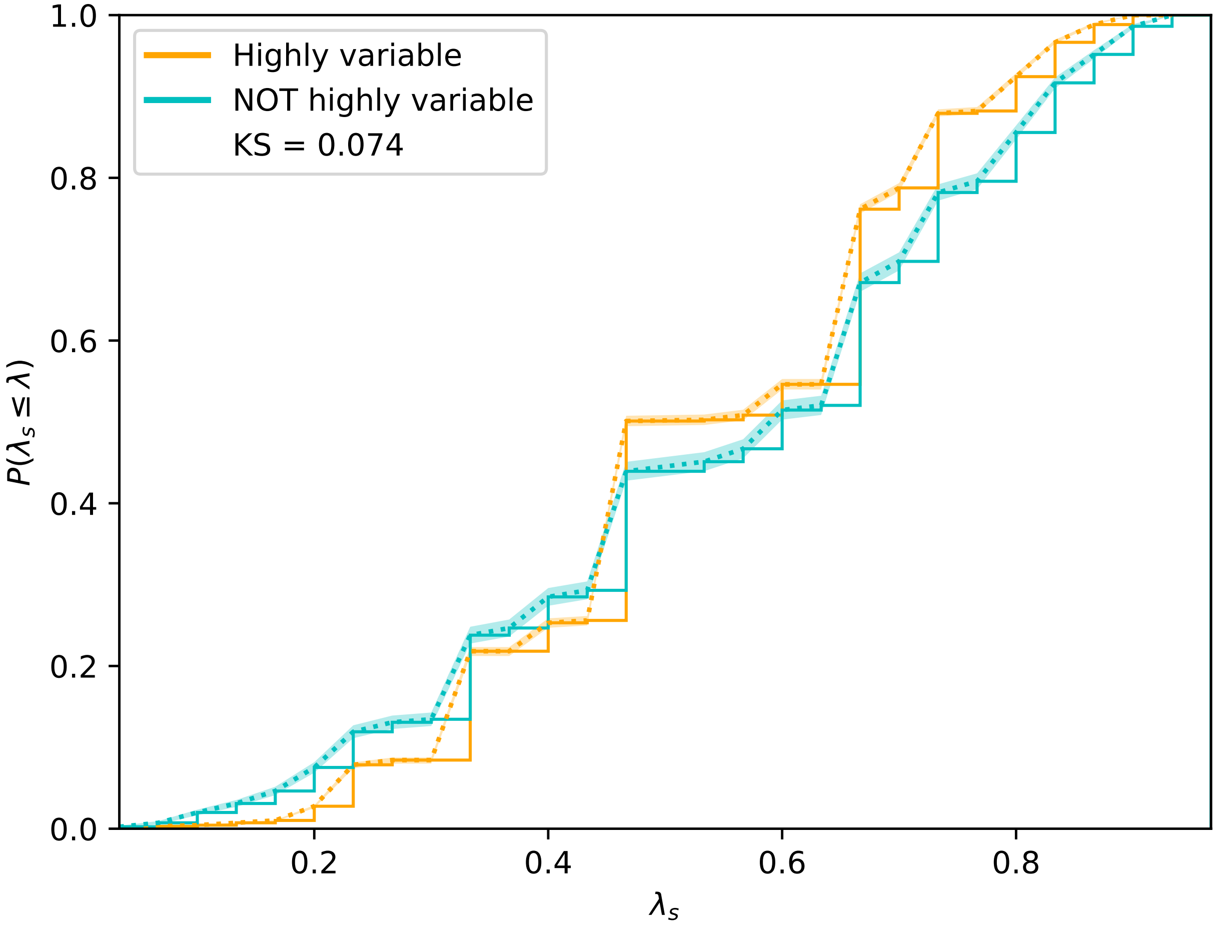

Supplementary Figure 45: Physical exercise: running.

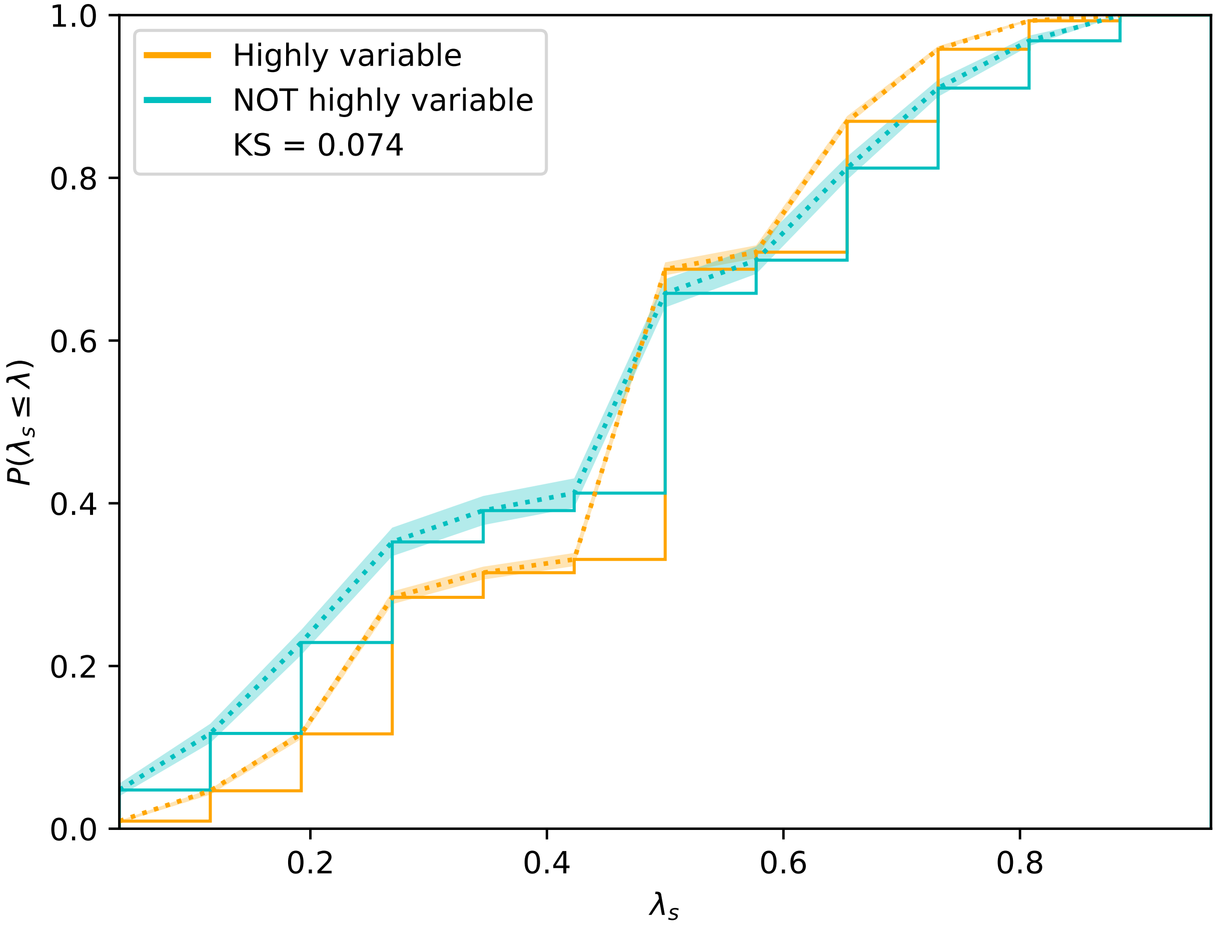

Supplementary Figure 46: Party-related experience: cigarettes.

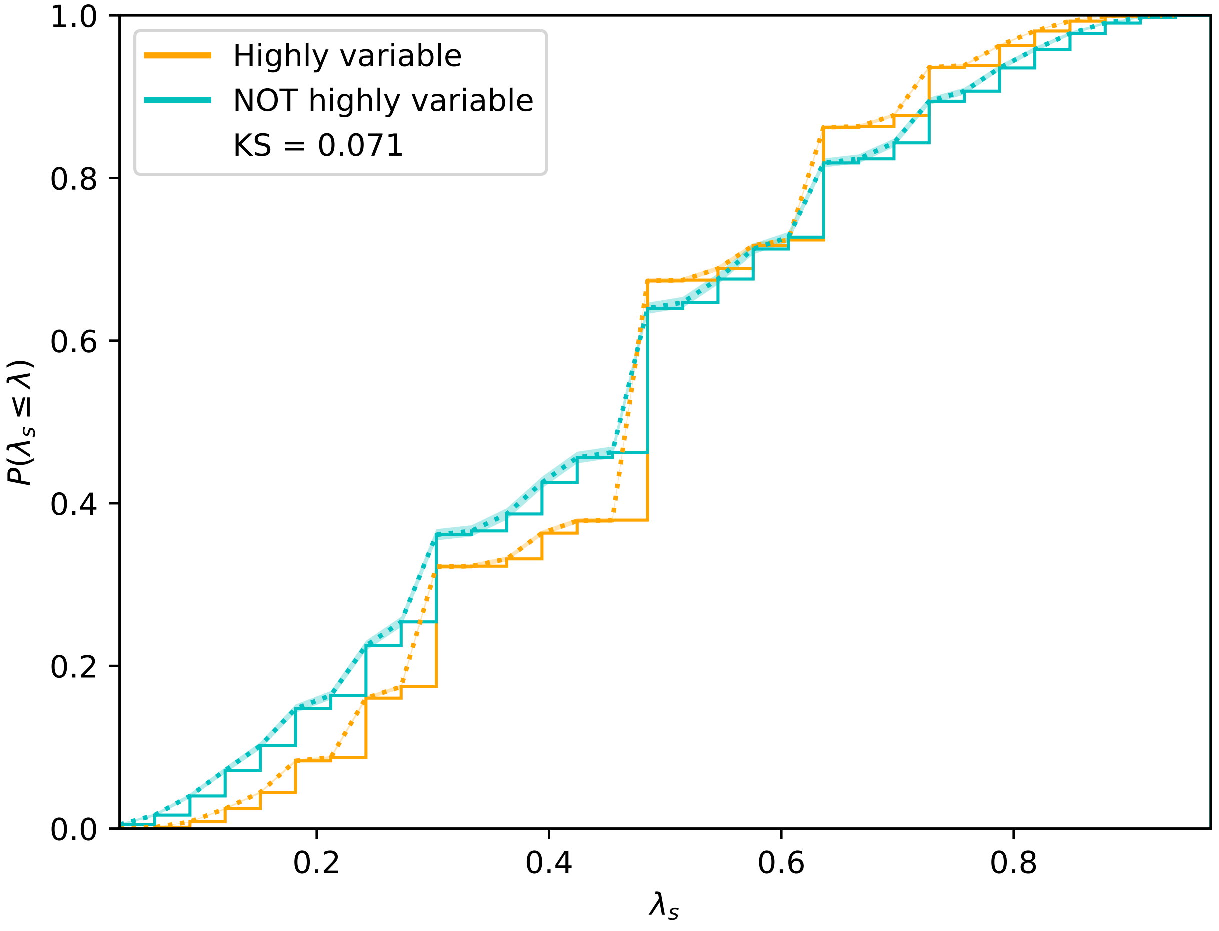

Supplementary Figure 47: Diarrhea stool health.

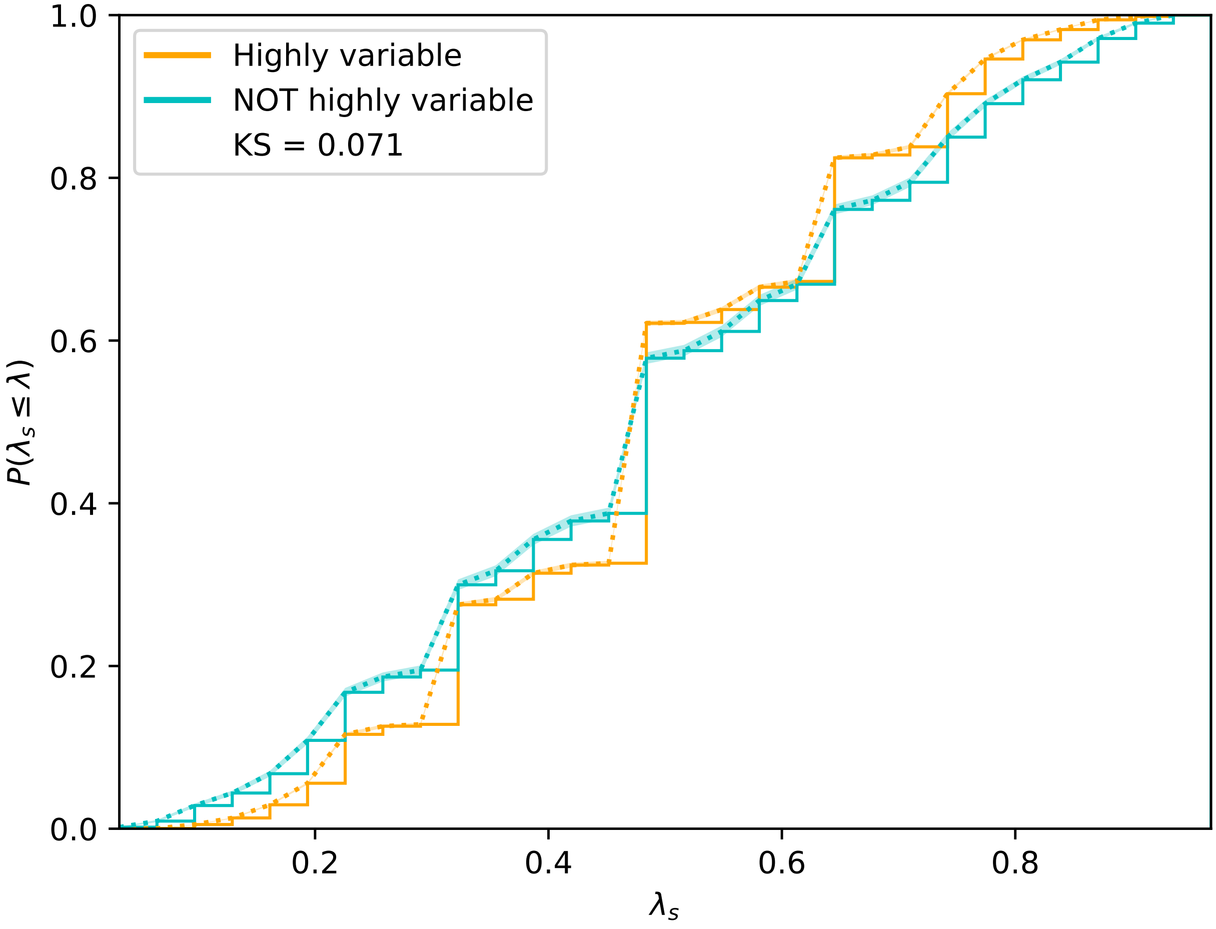

Supplementary Figure 48: Productive motivation level.

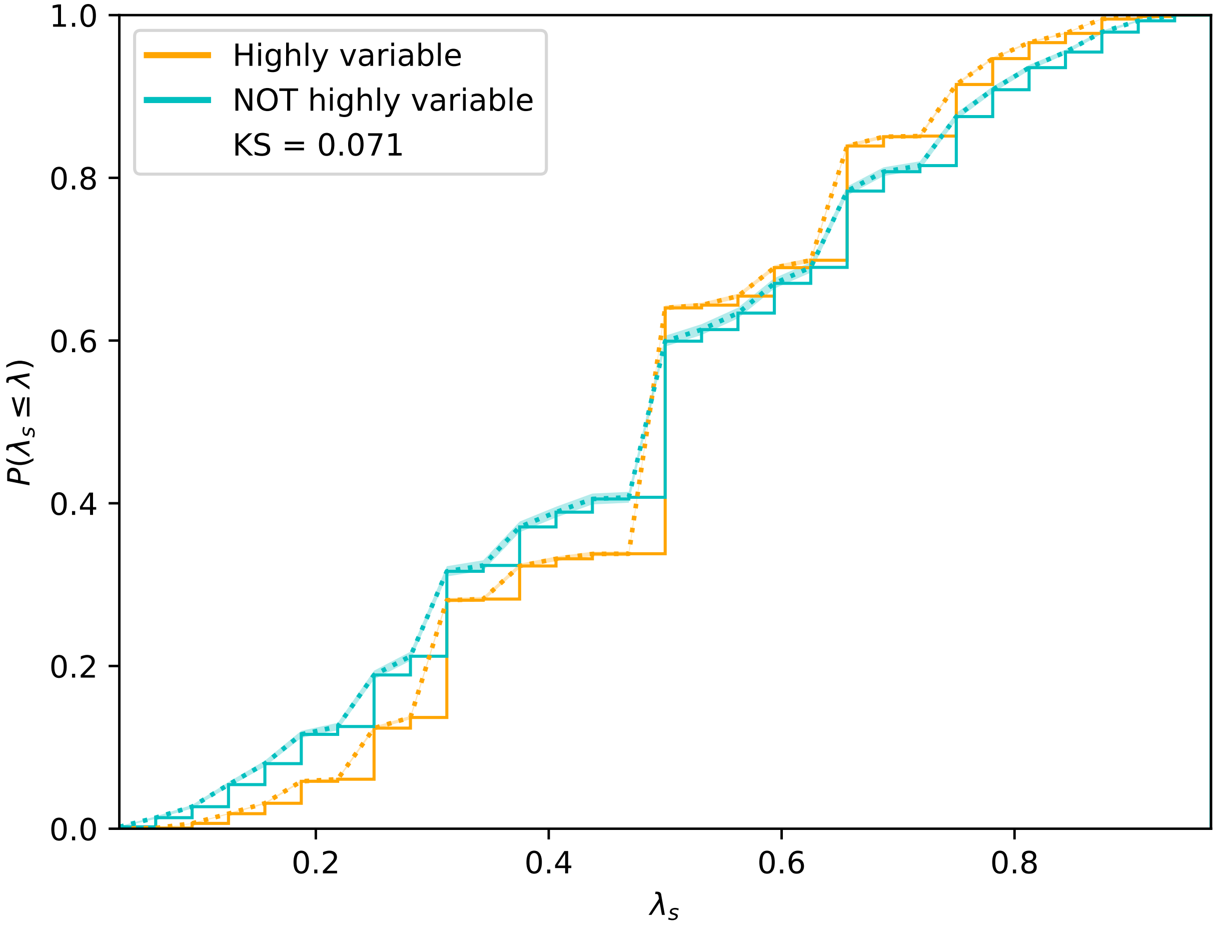

Supplementary Figure 49: Chocolate food craving experienced.

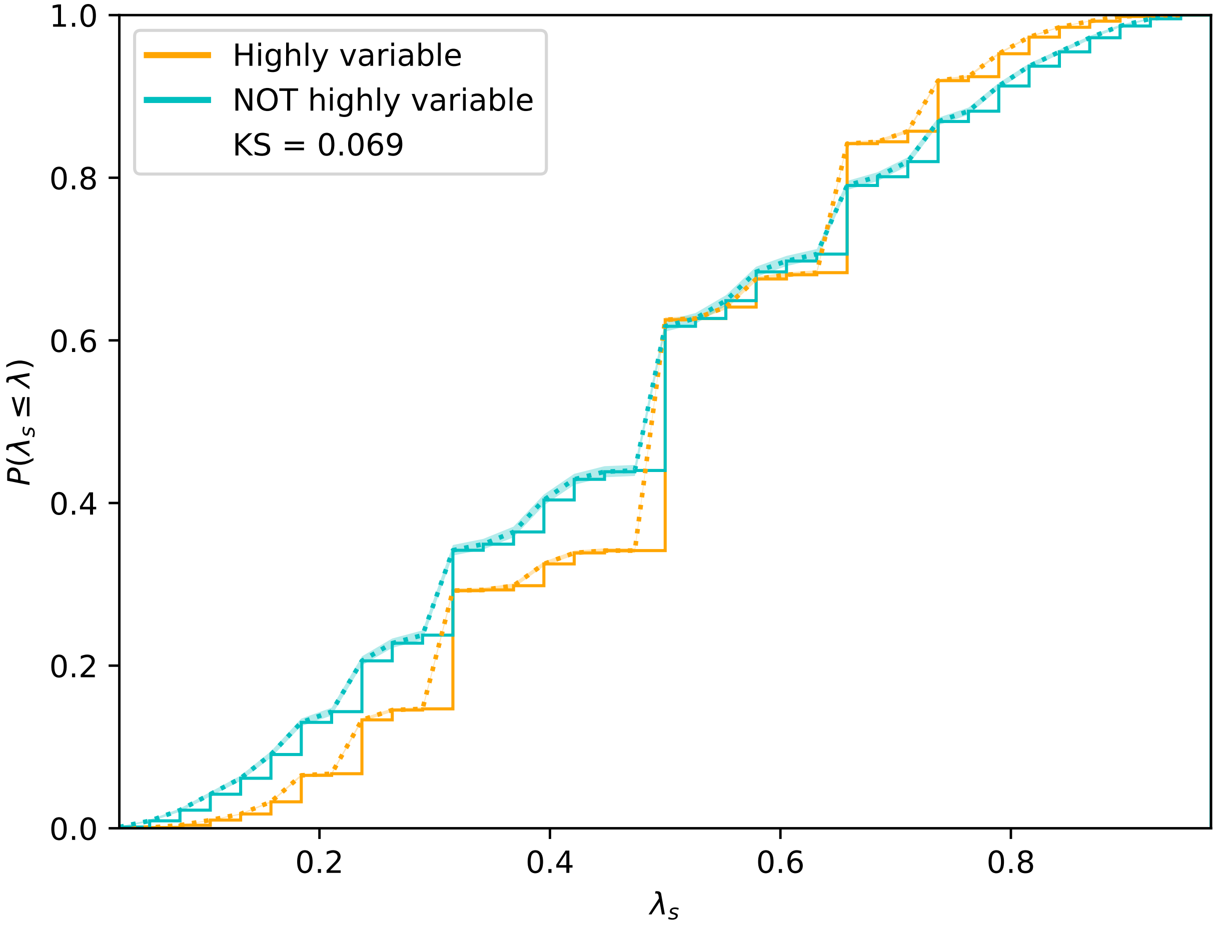

Supplementary Figure 50: Focused mental state.

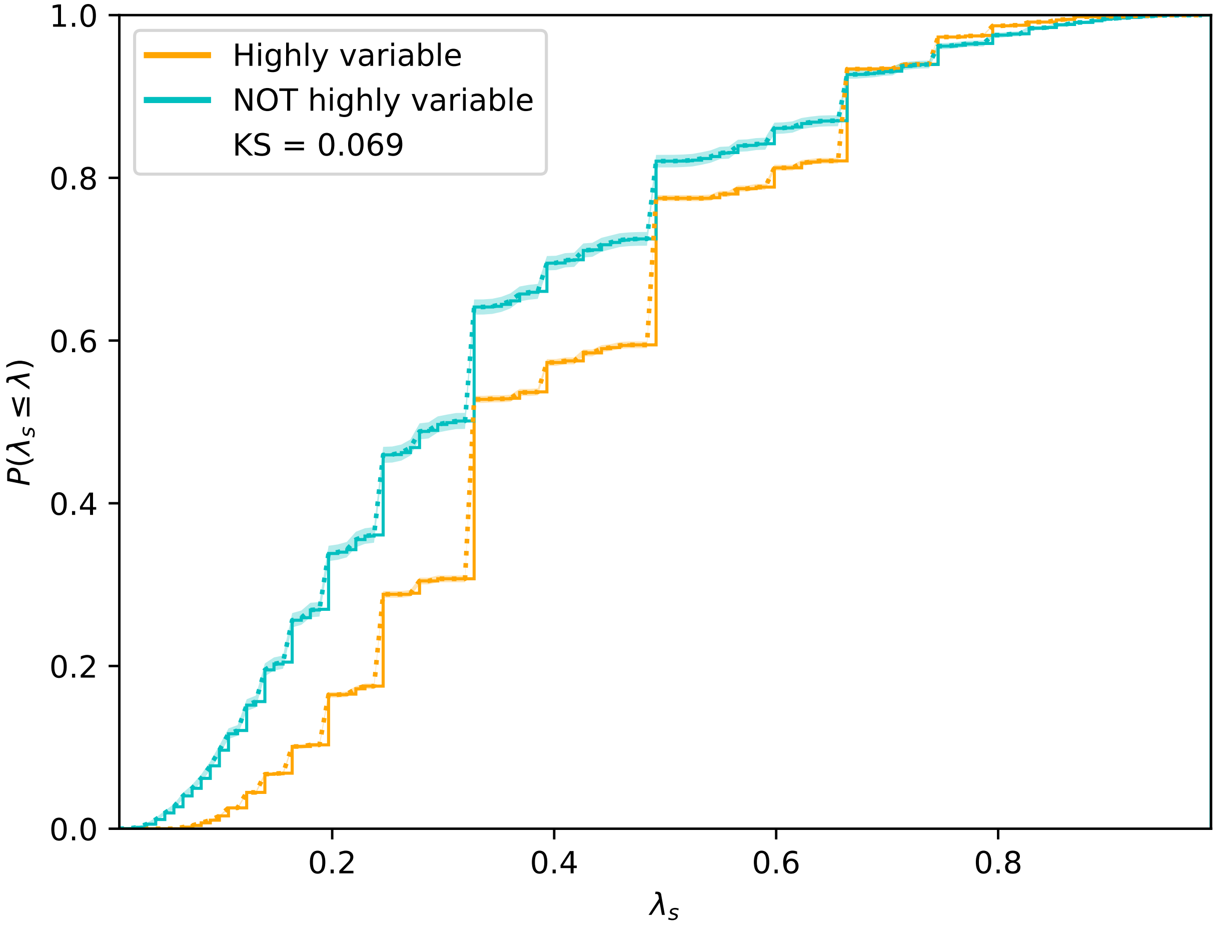

Supplementary Figure 51: Atypical vaginal discharge type.

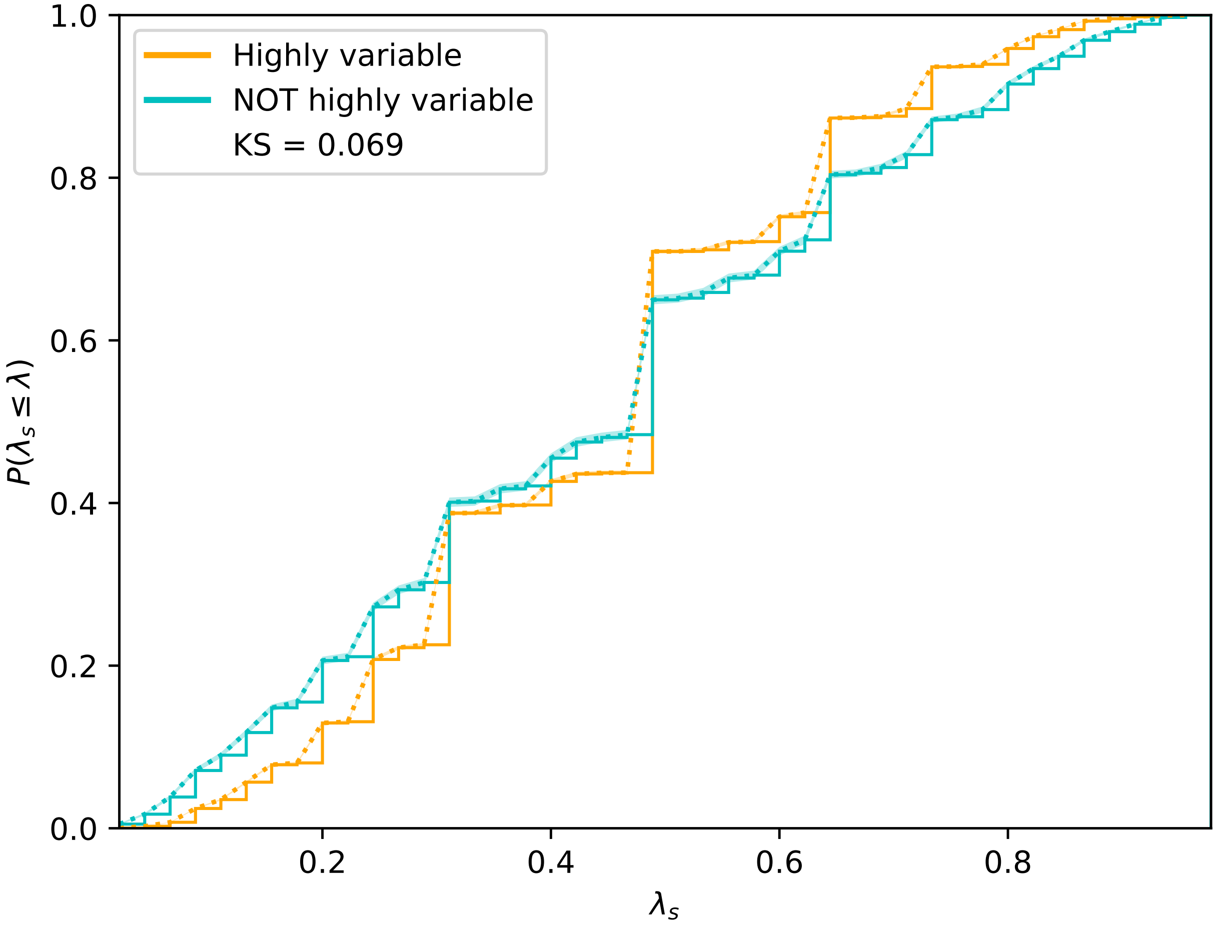

Supplementary Figure 52: Protected sex reported.

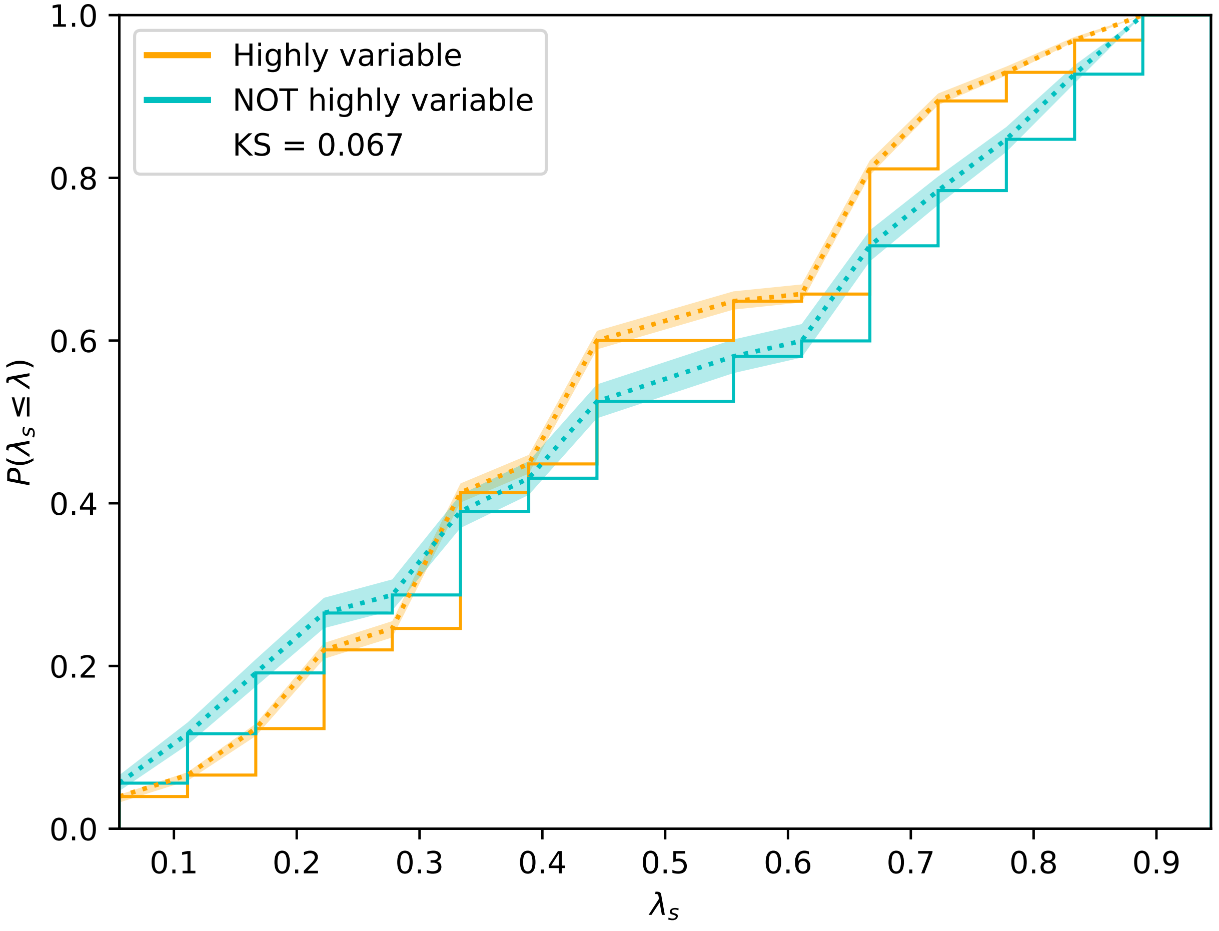

Supplementary Figure 53: Menstrual cup method used for period collection.

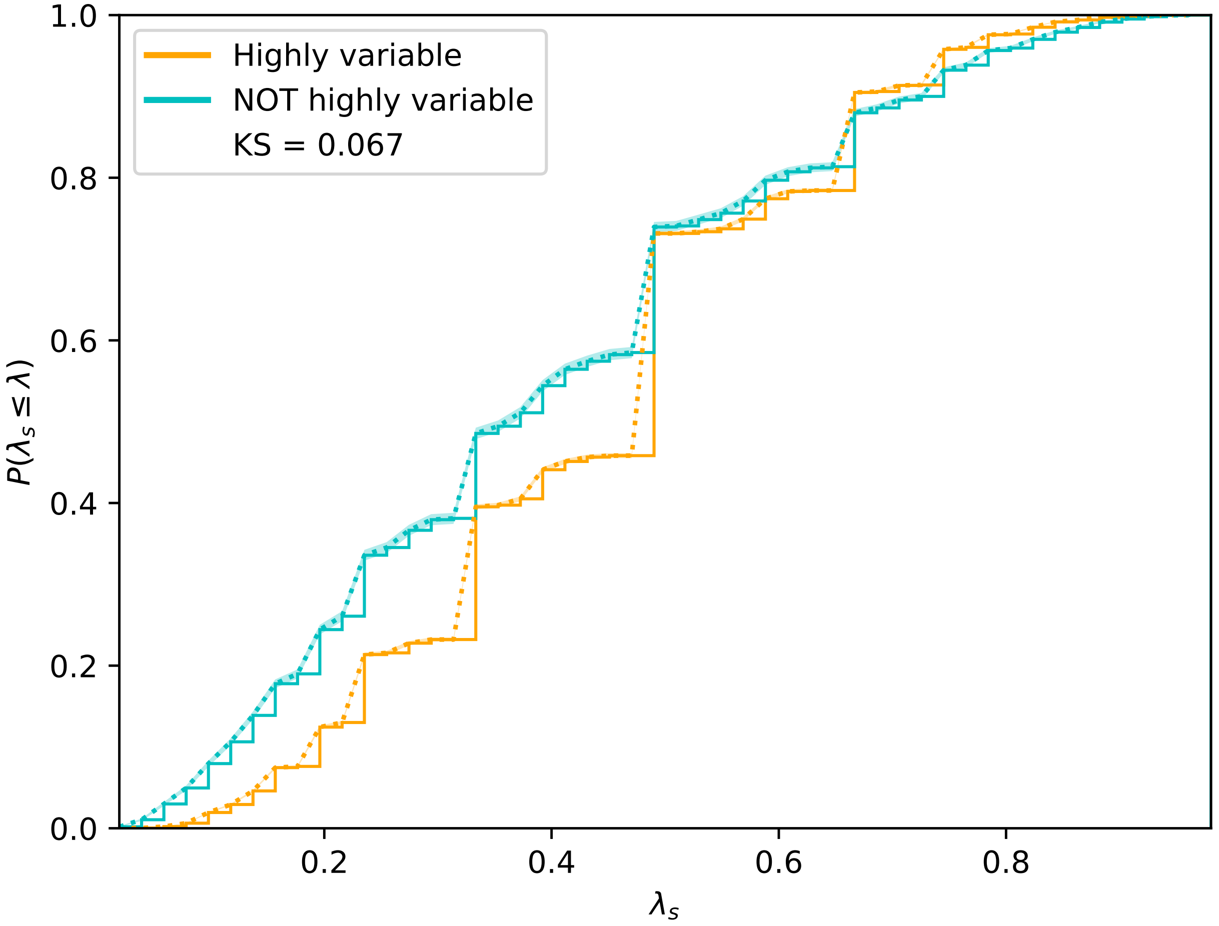

Supplementary Figure 54: Dry skin health reported.

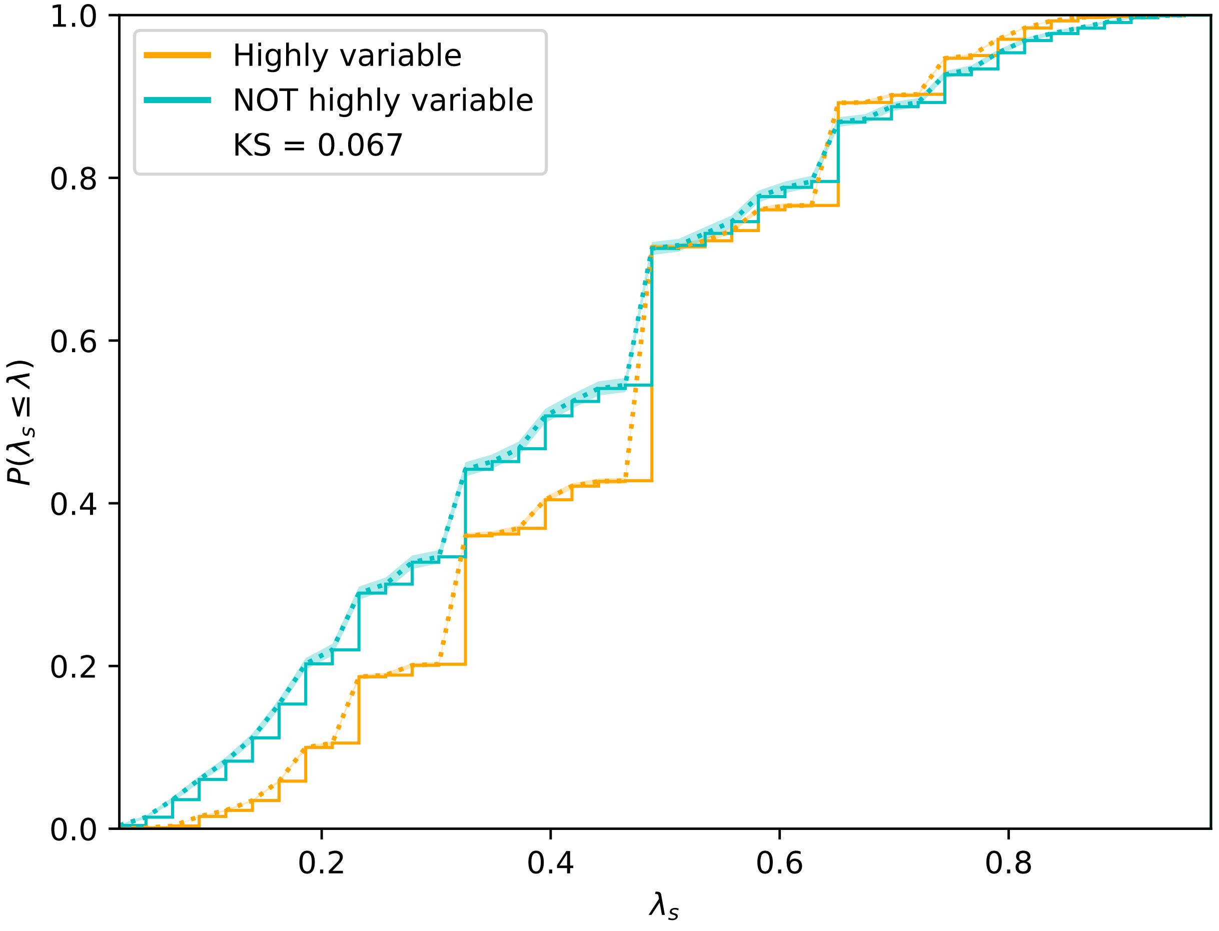

Supplementary Figure 55: Dry hair reported.

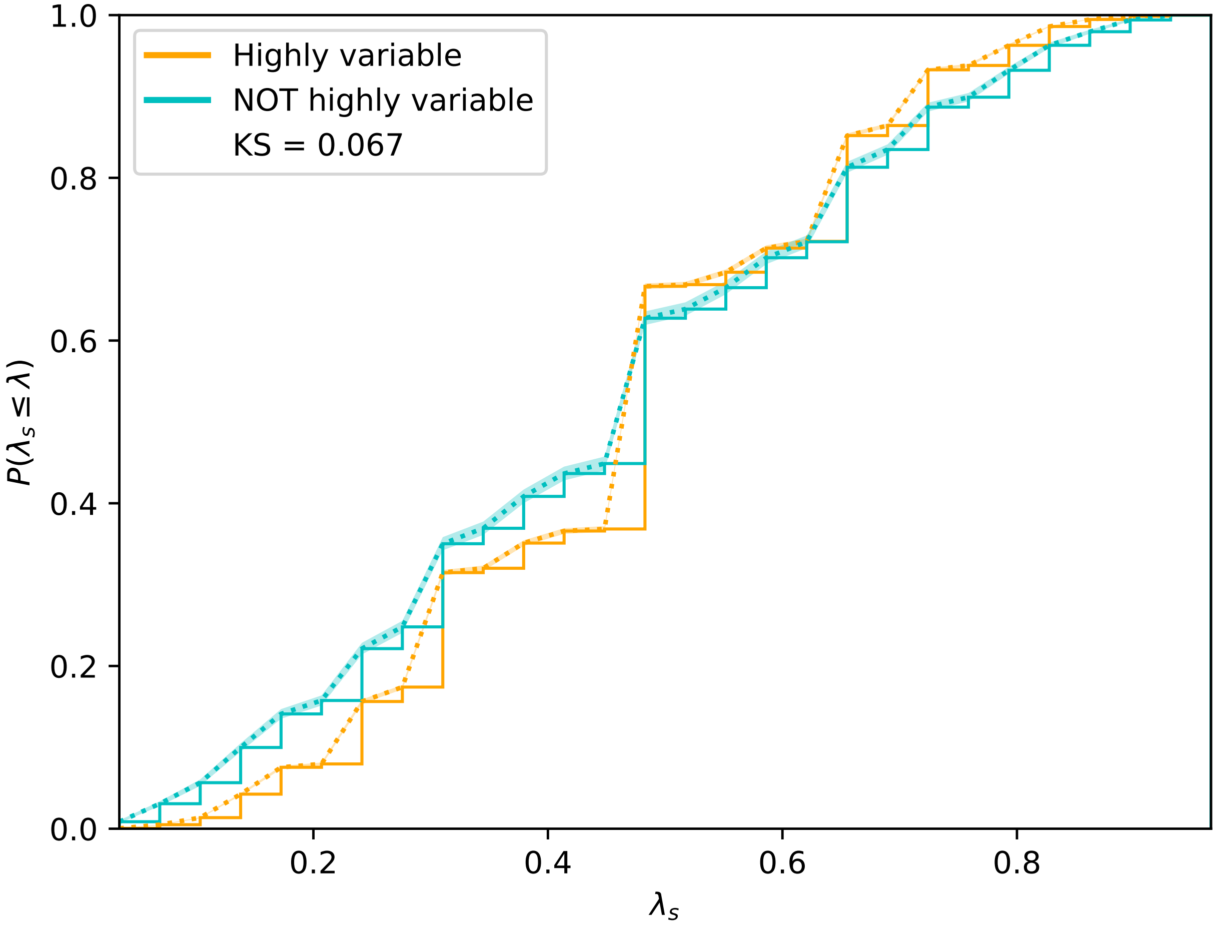

Supplementary Figure 56: Oily hair reported.

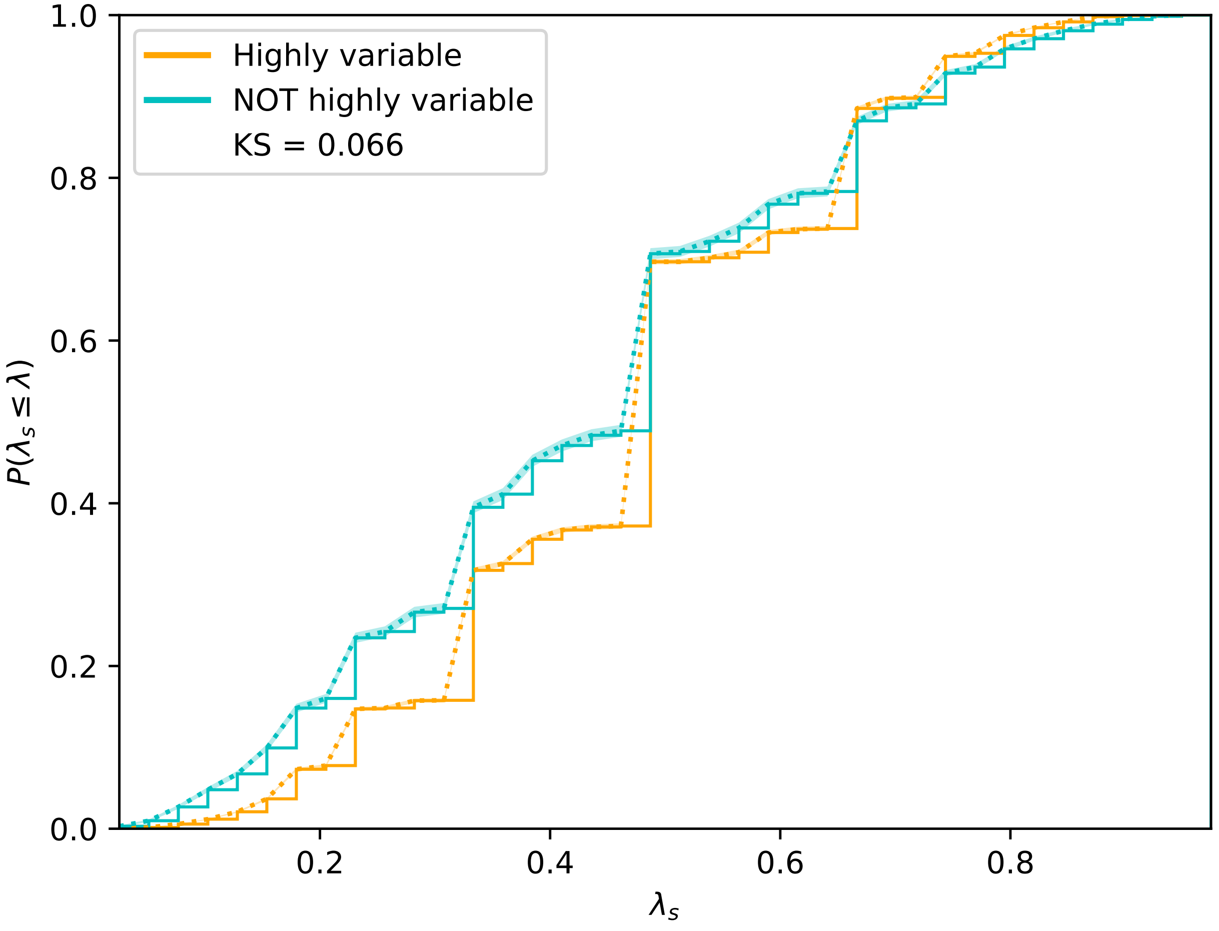

Supplementary Figure 57: Sticky vaginal discharge type.

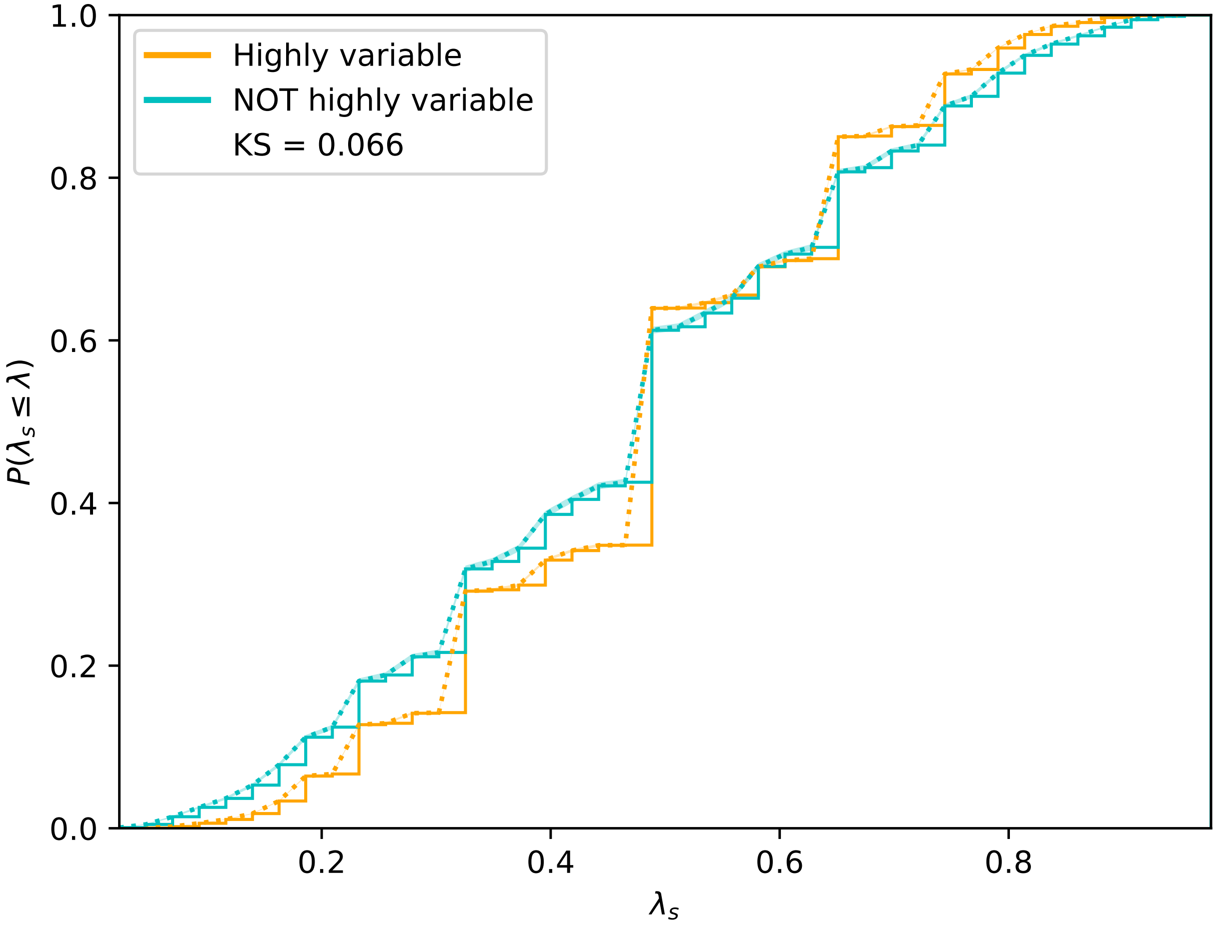

Supplementary Figure 58: Exhausted energy level.

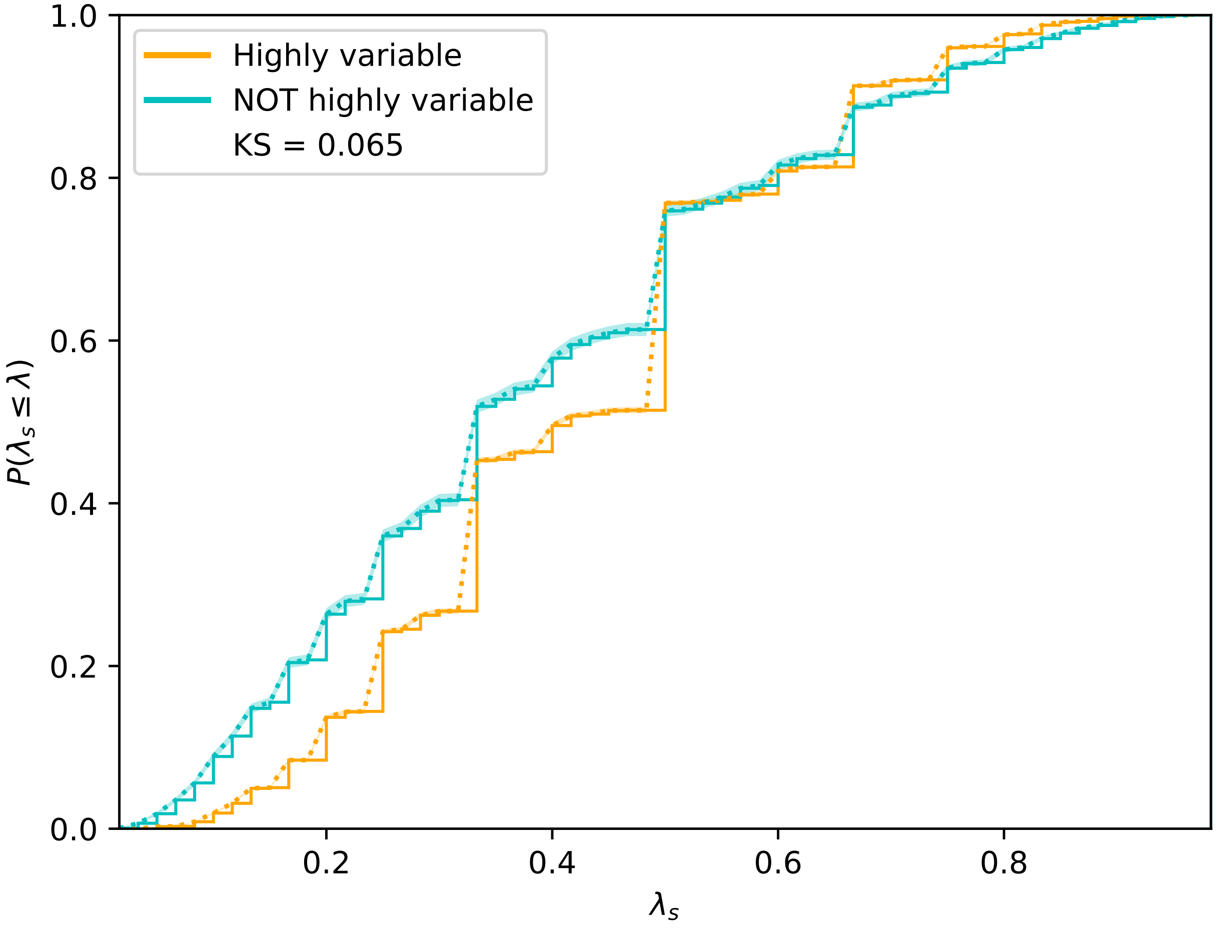

Supplementary Figure 59: Great stool health.

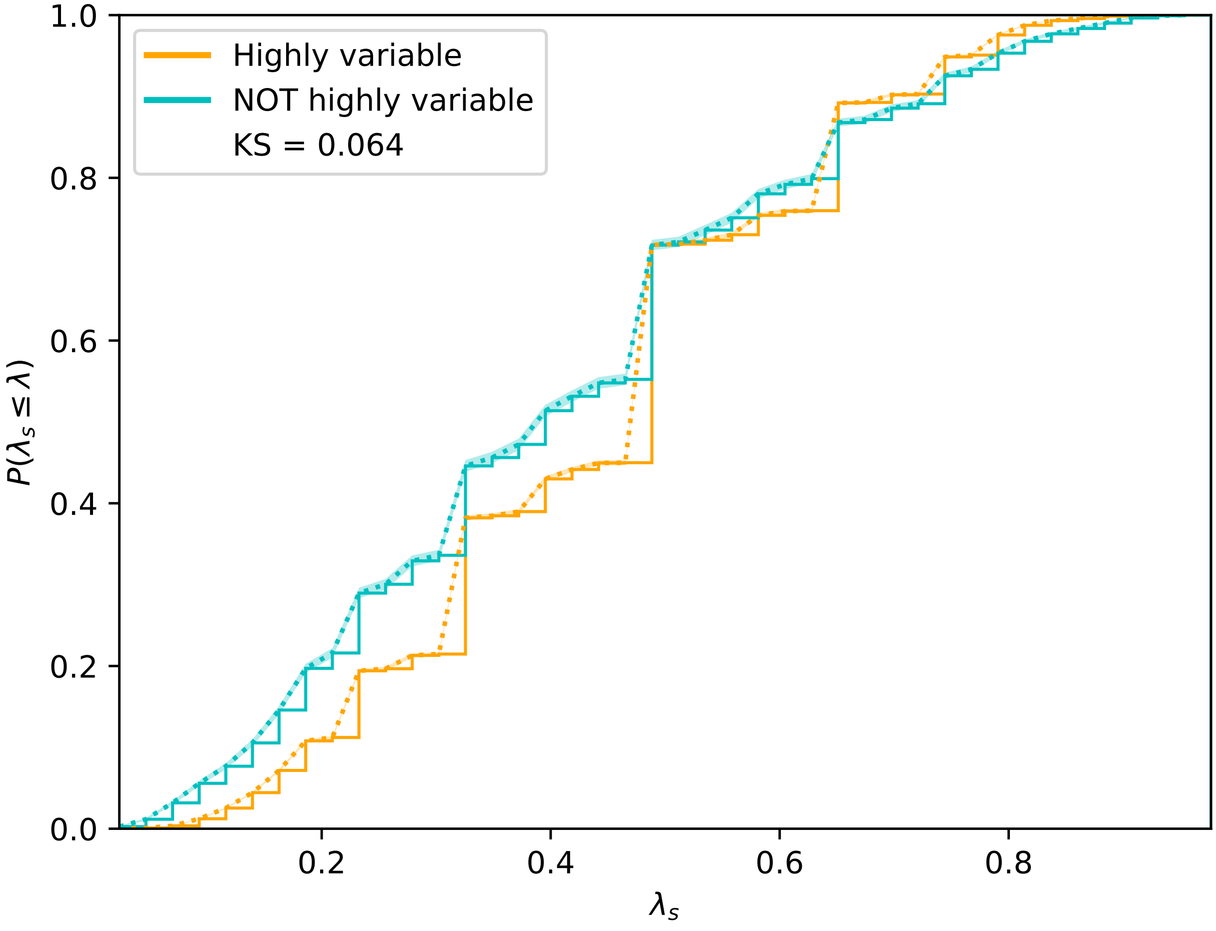

Supplementary Figure 60: Nauseated digestive health.

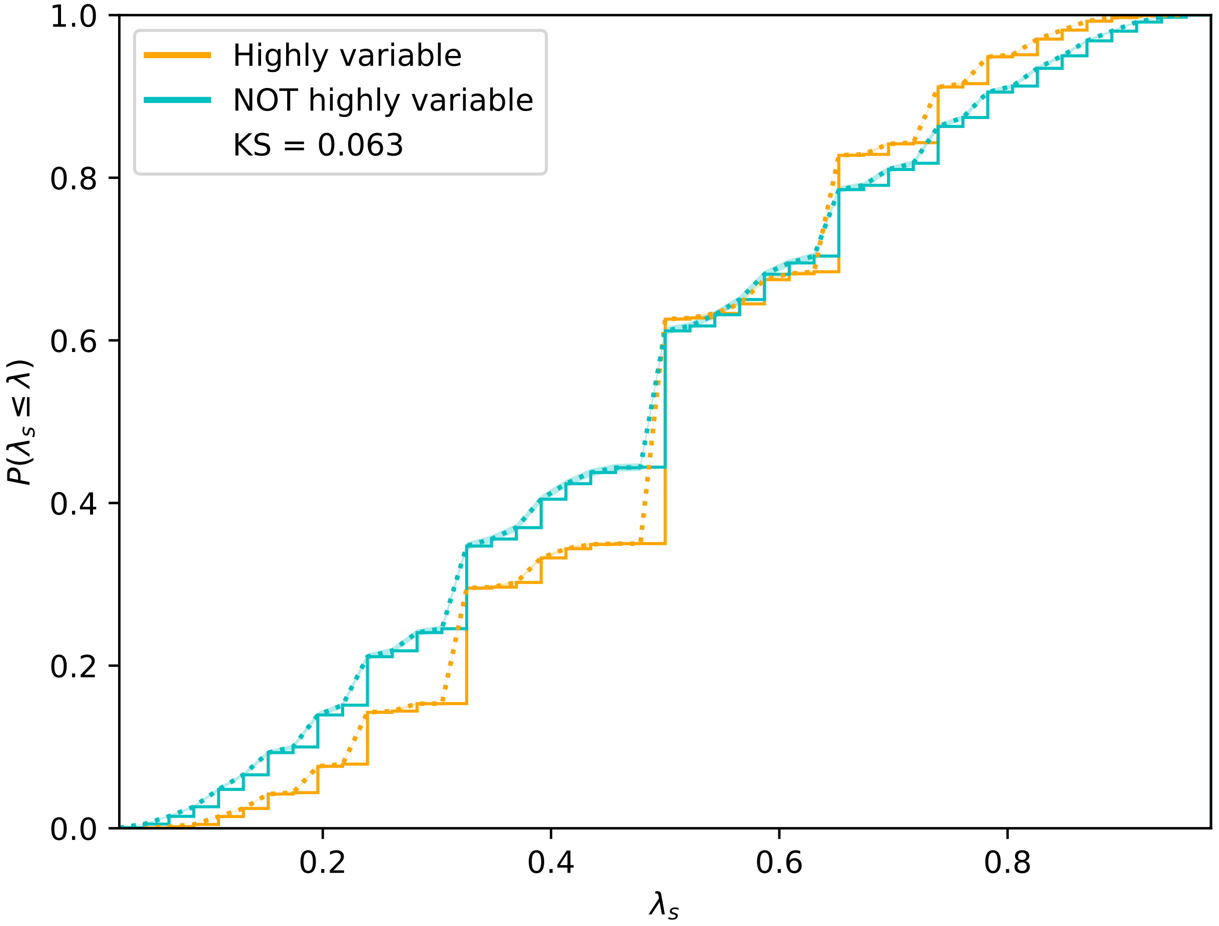

Supplementary Figure 61: High energy level.

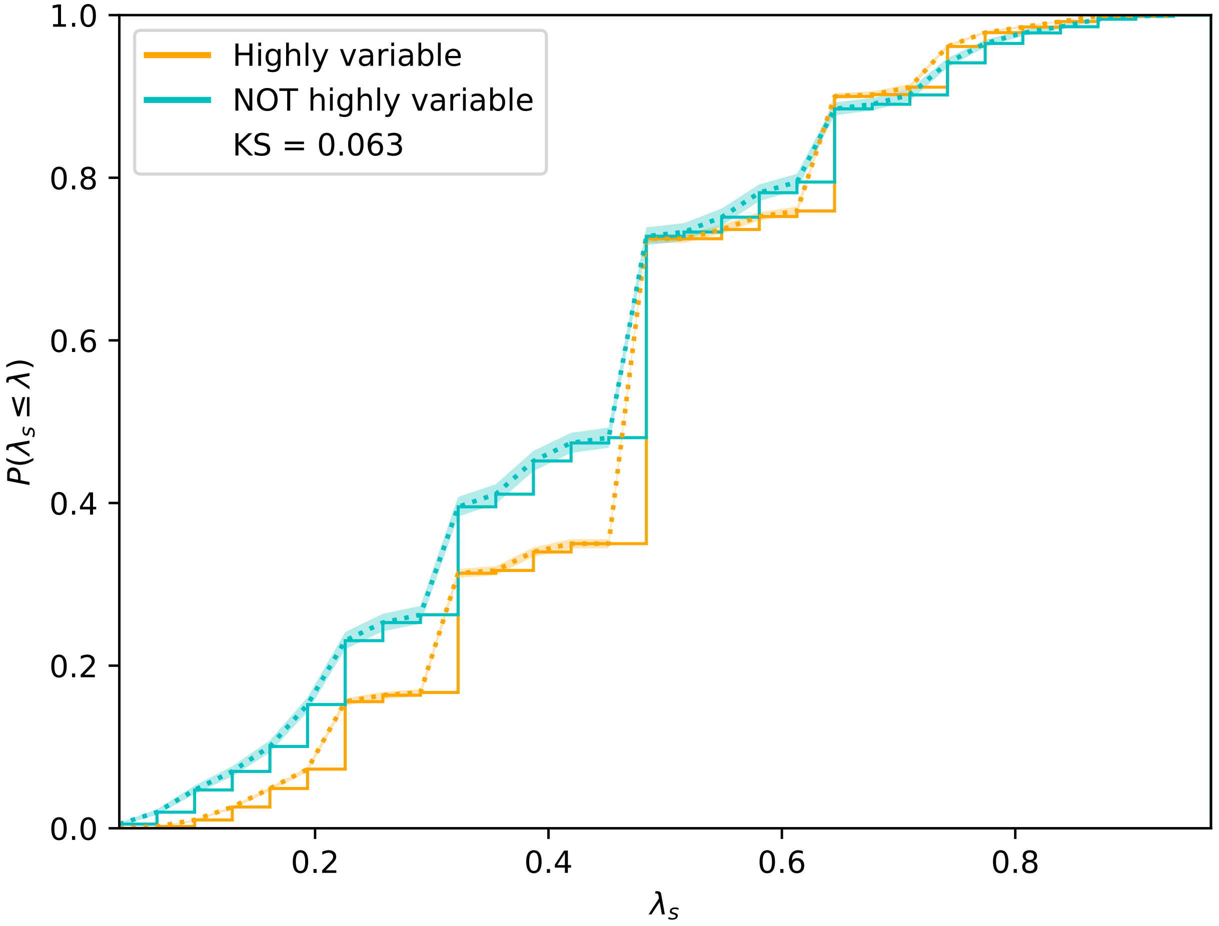

Supplementary Figure 62: Party-related experience: big night party.

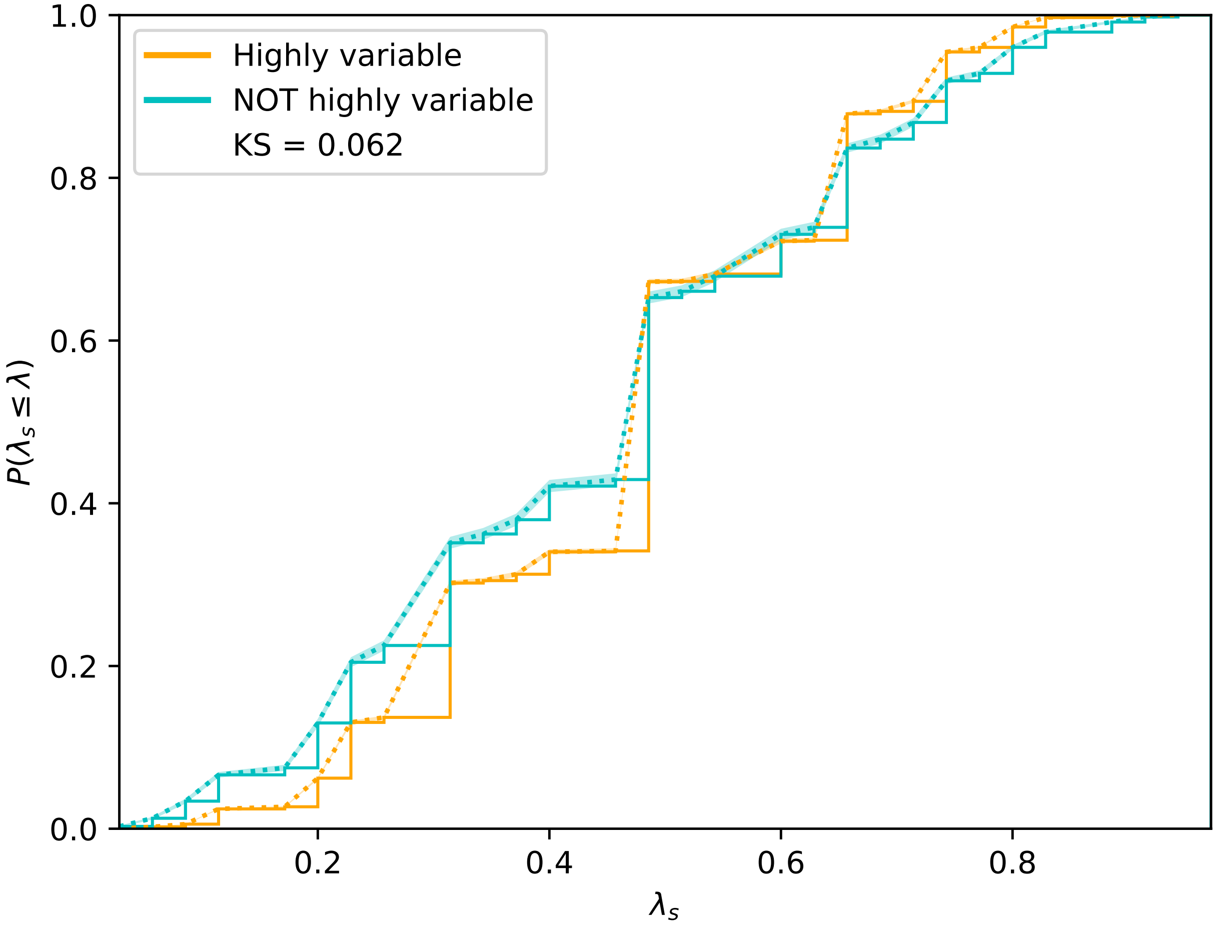

Supplementary Figure 63: Conflict social behavior.

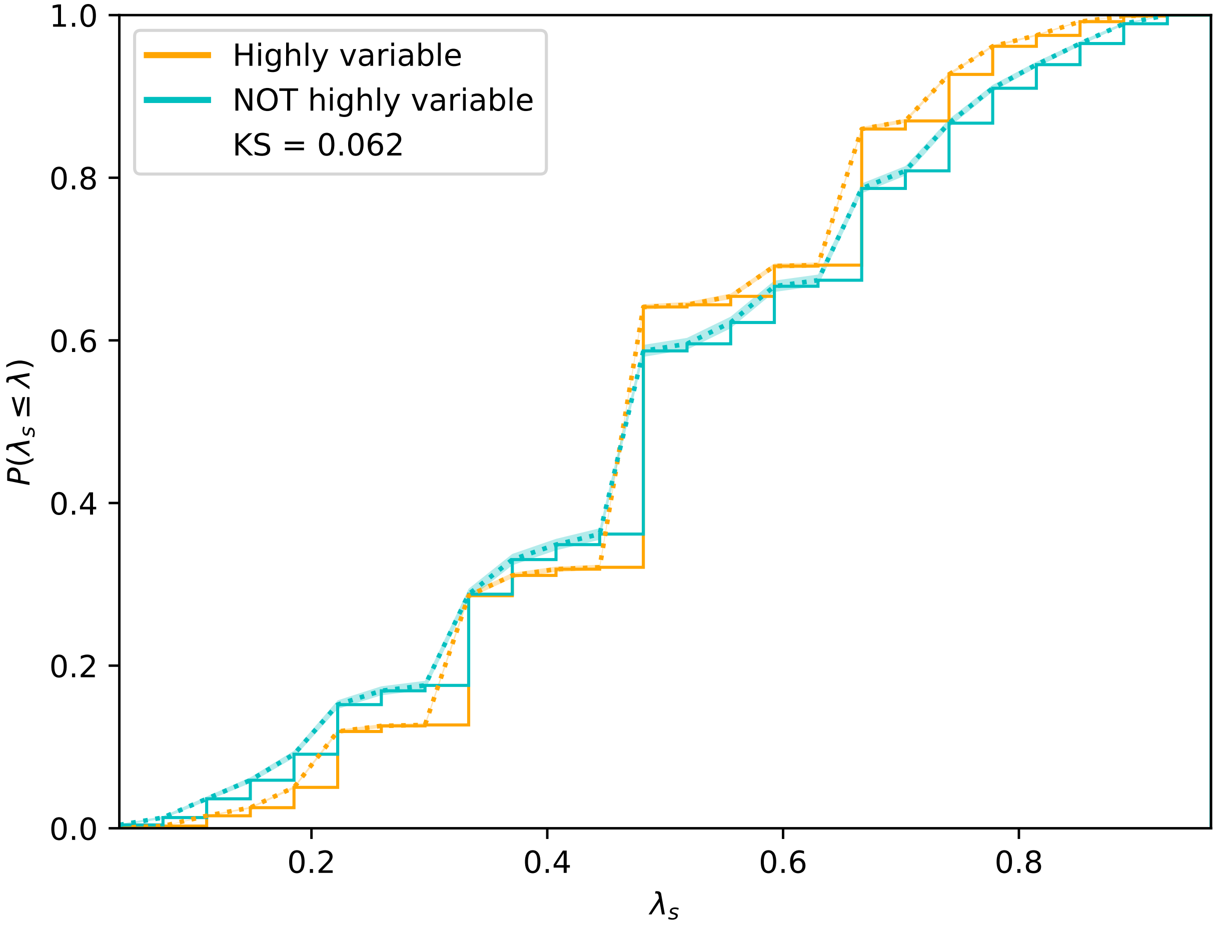

Supplementary Figure 64: Egg white vaginal discharge type.

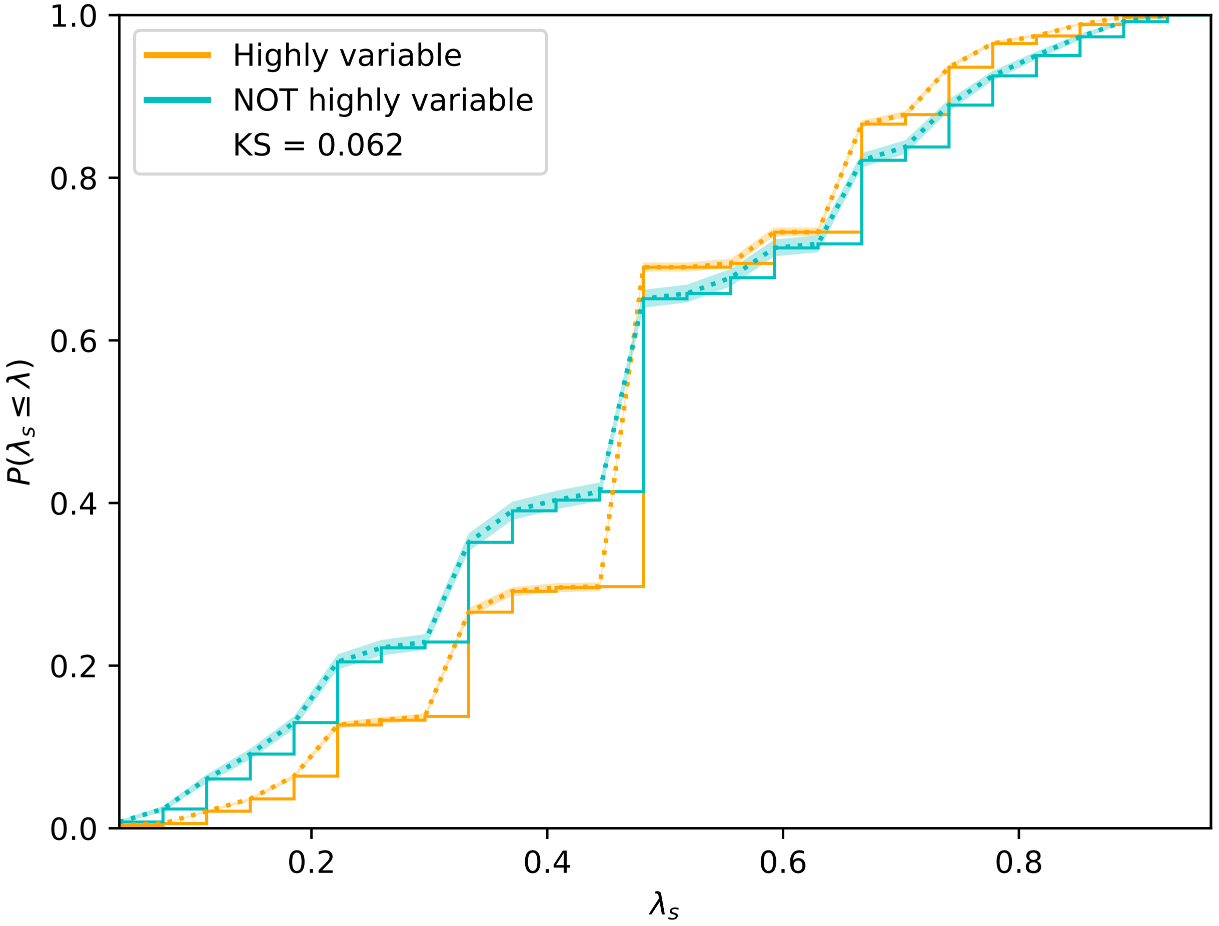

Supplementary Figure 65: Physical exercise: yoga.

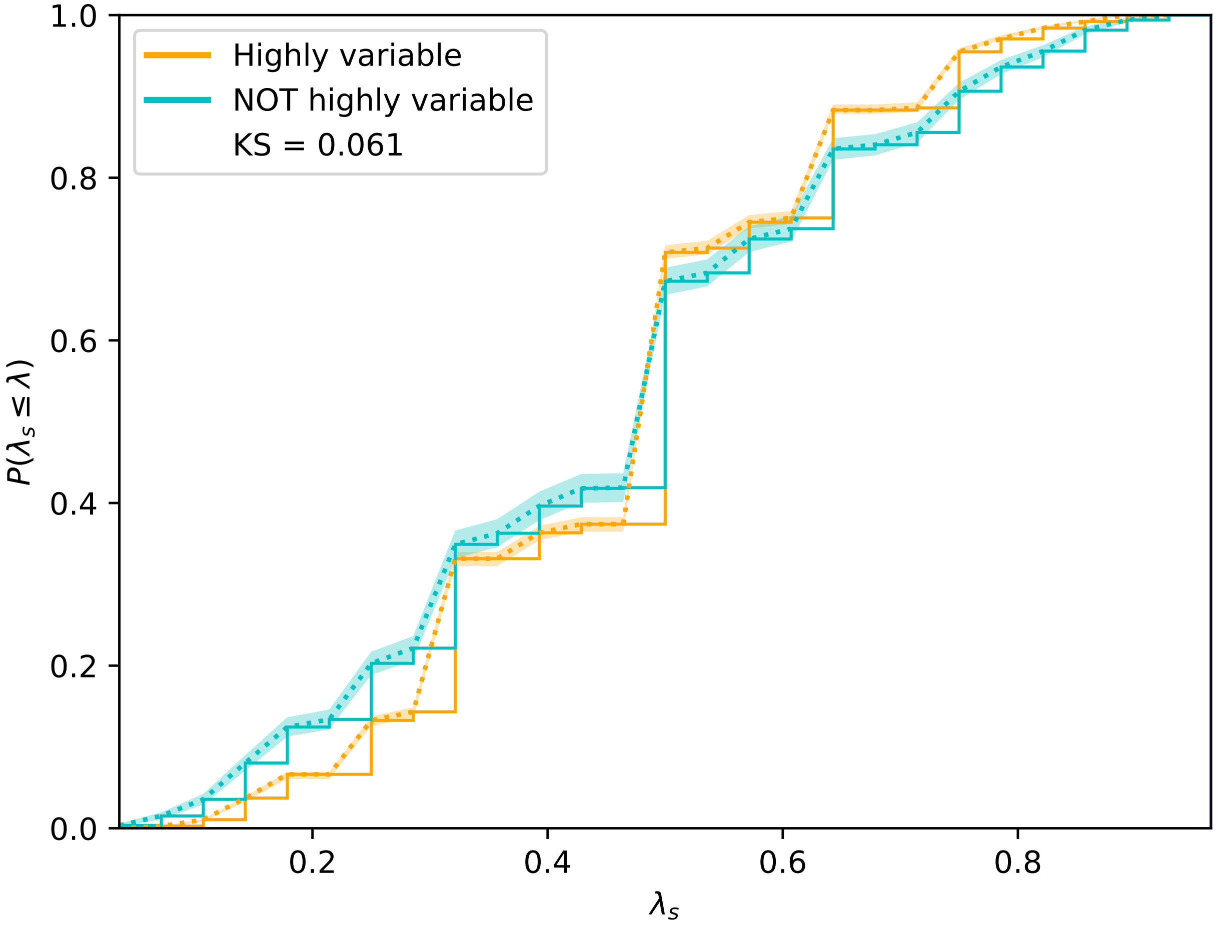

Supplementary Figure 66: Physical maladies: allergy.

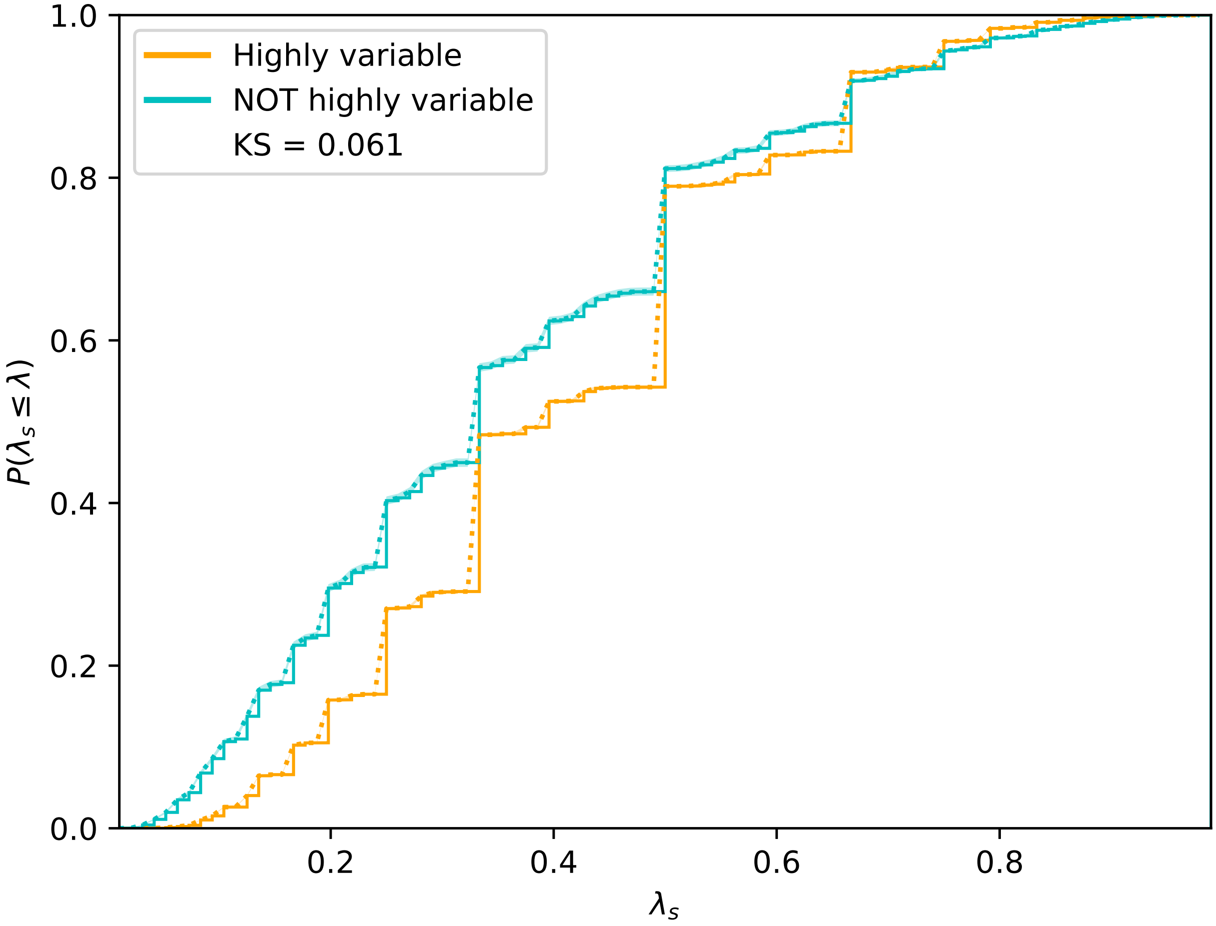

Supplementary Figure 67: >9 hours of sleep.

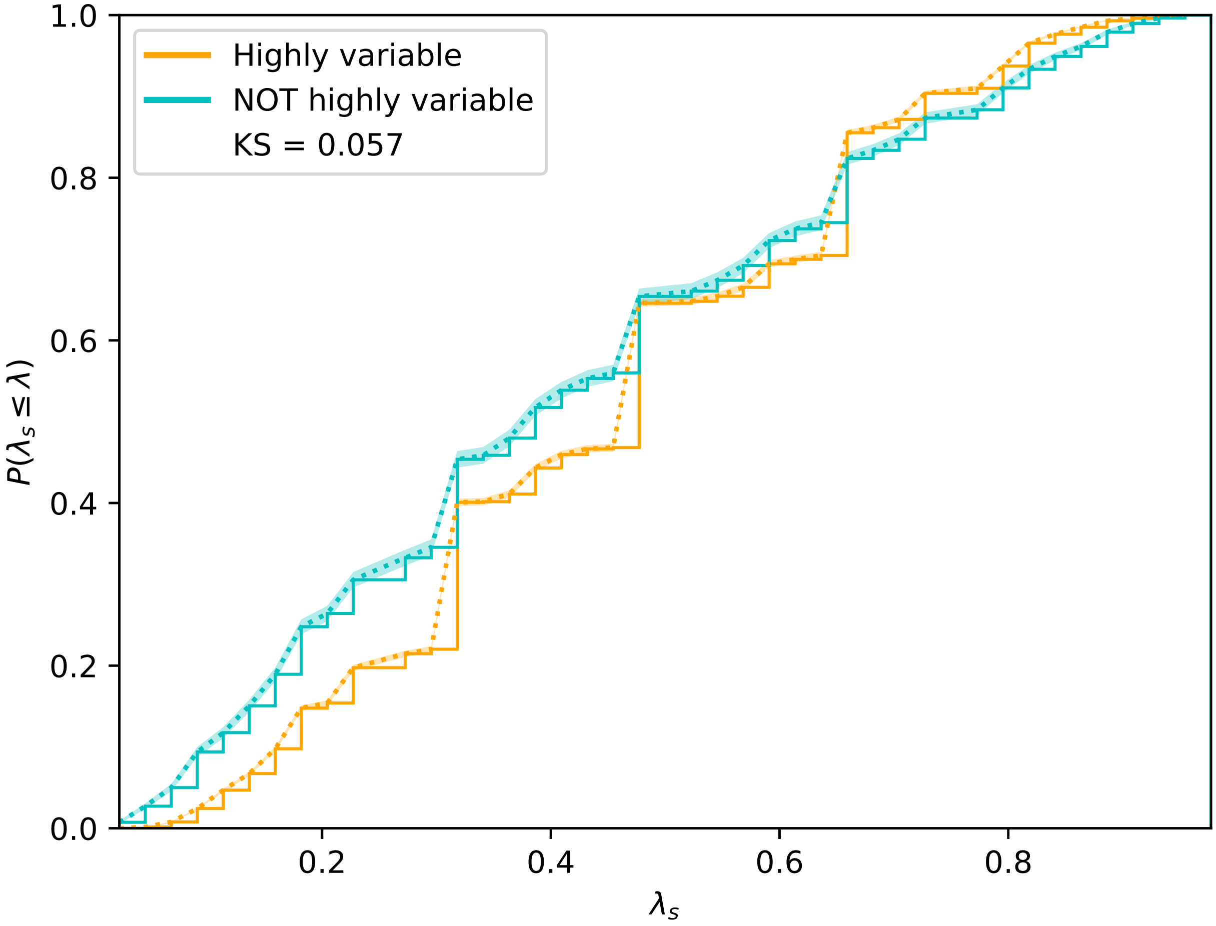

Supplementary Figure 68: Panty liner method used for period collection.

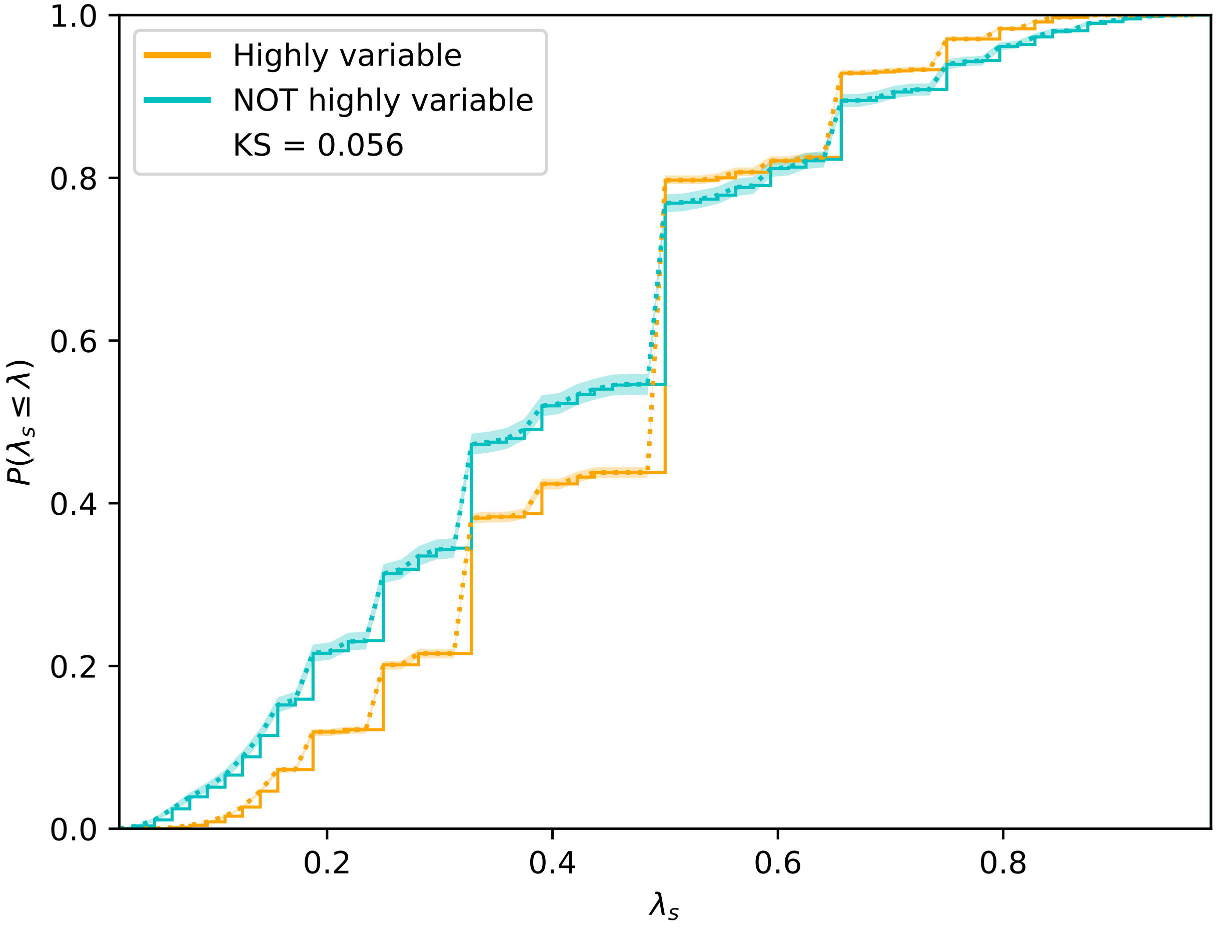

Supplementary Figure 69: Physical exercise: biking.

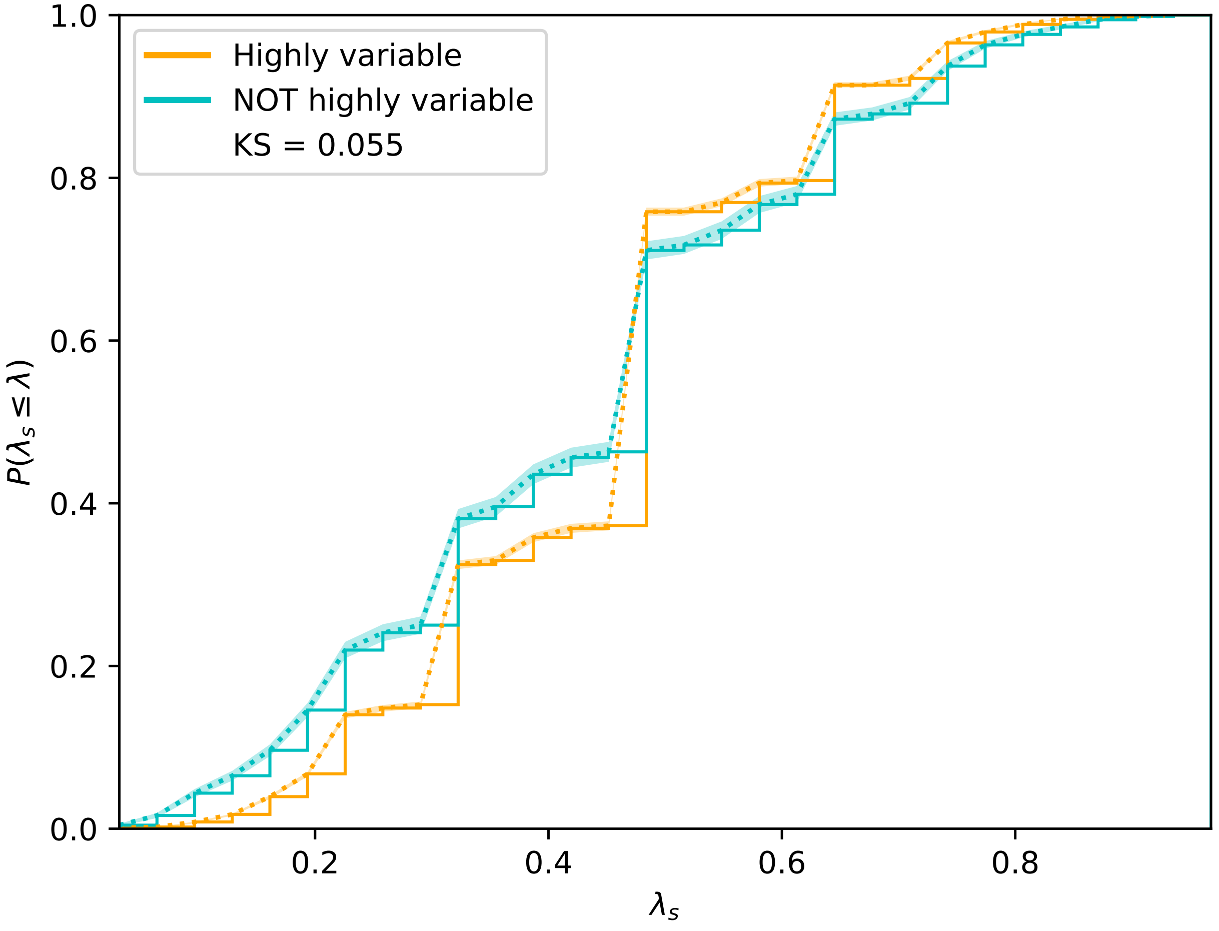

Supplementary Figure 70: Party-related experience: hangover.

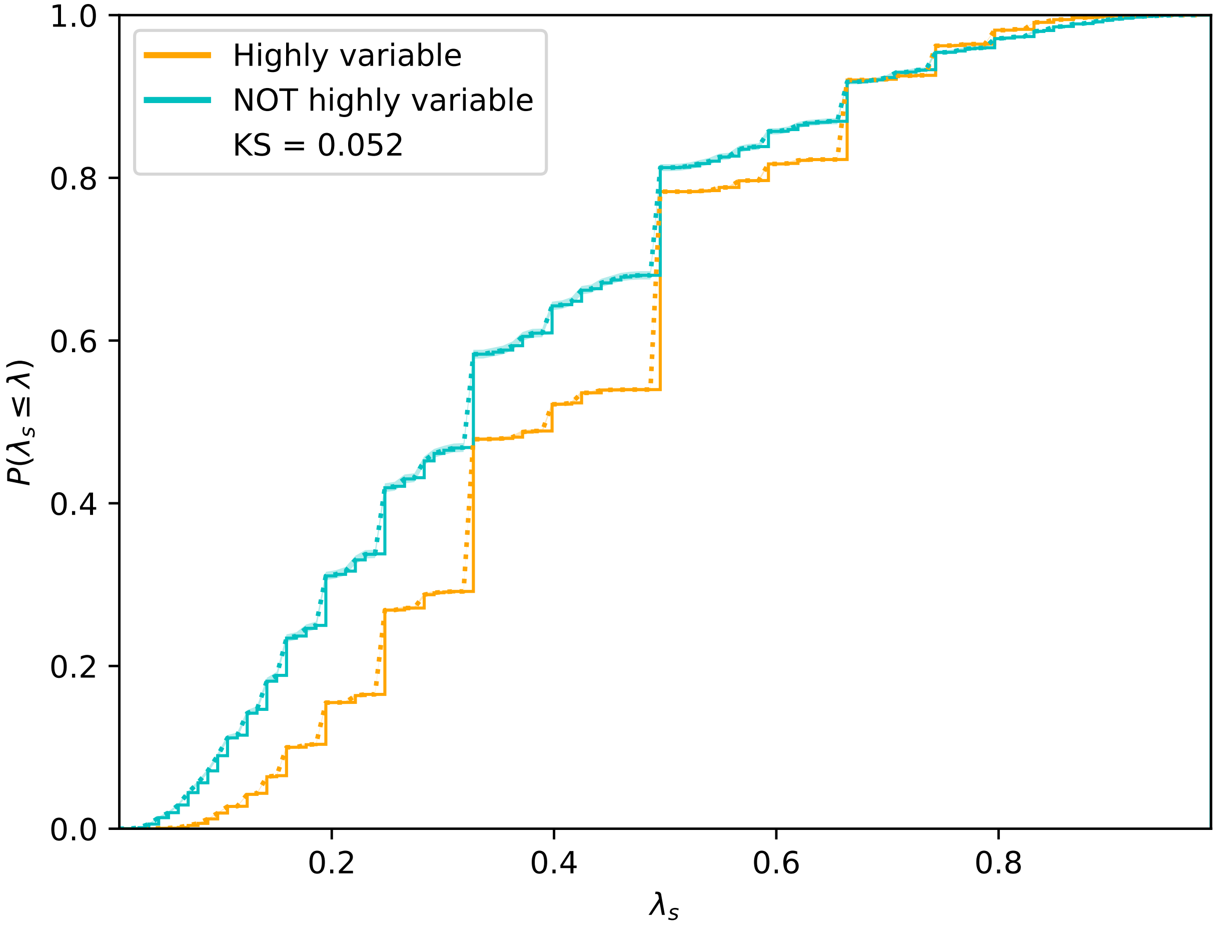

Supplementary Figure 71: Energized energy level.

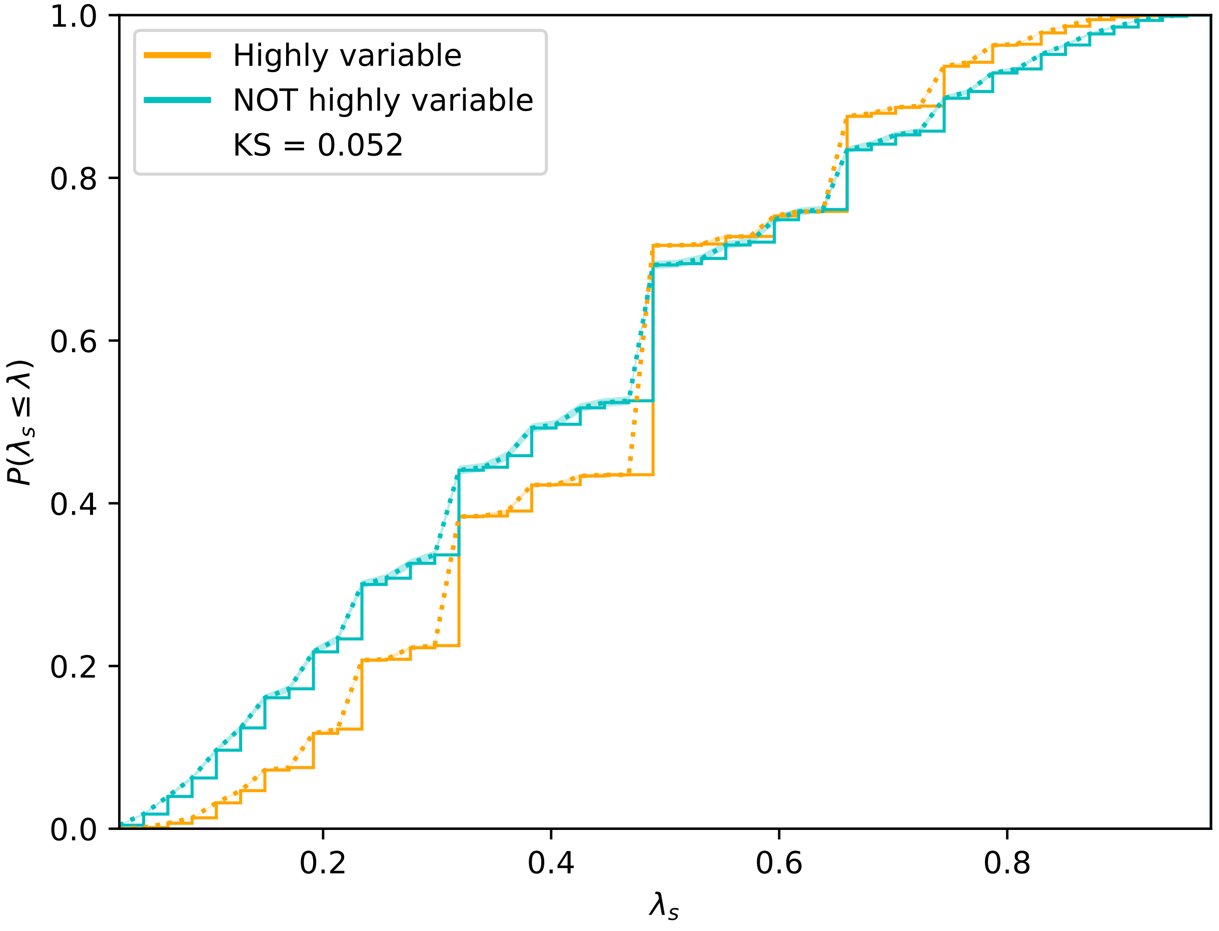

Supplementary Figure 72: High sex drive reported.

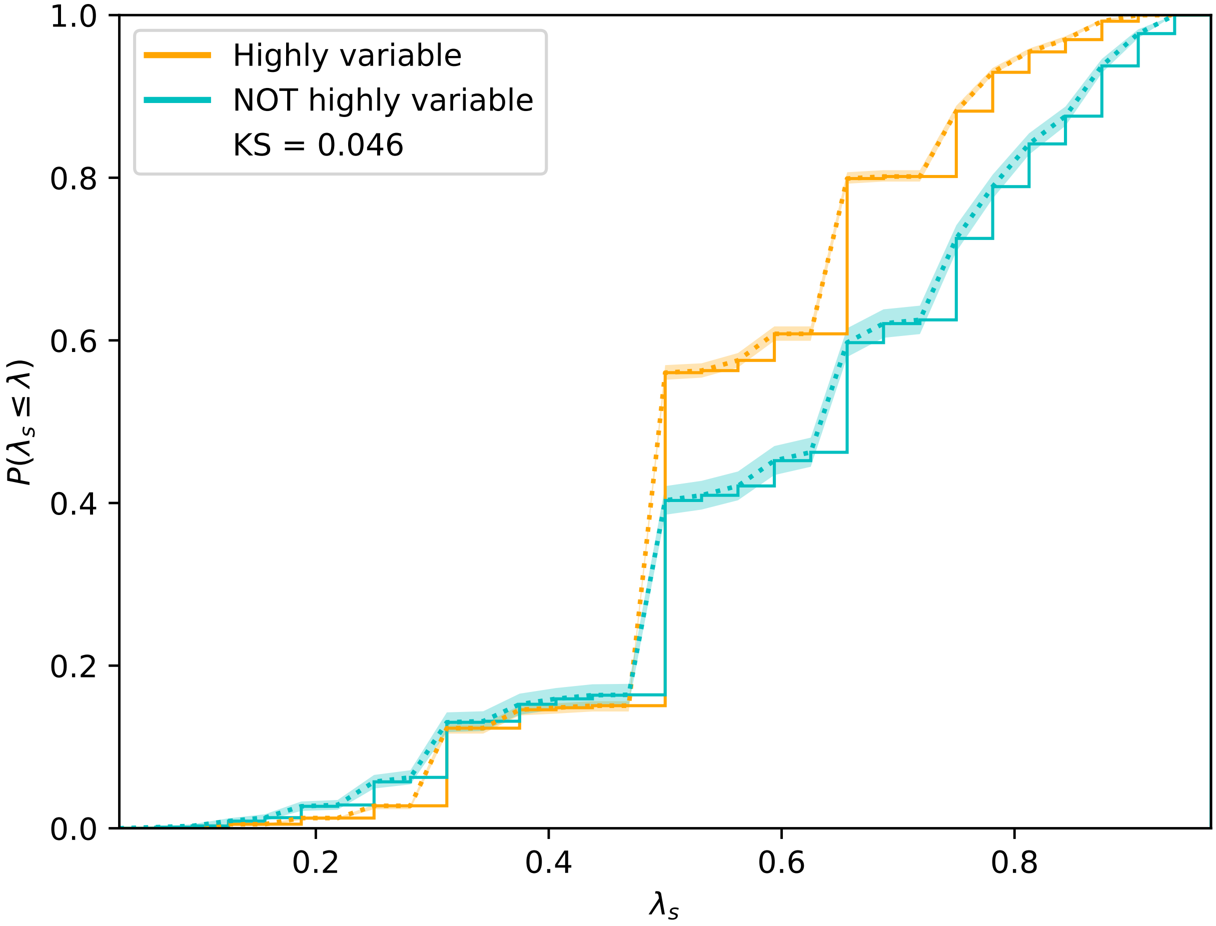

Supplementary Figure 73: Pain medication taken.

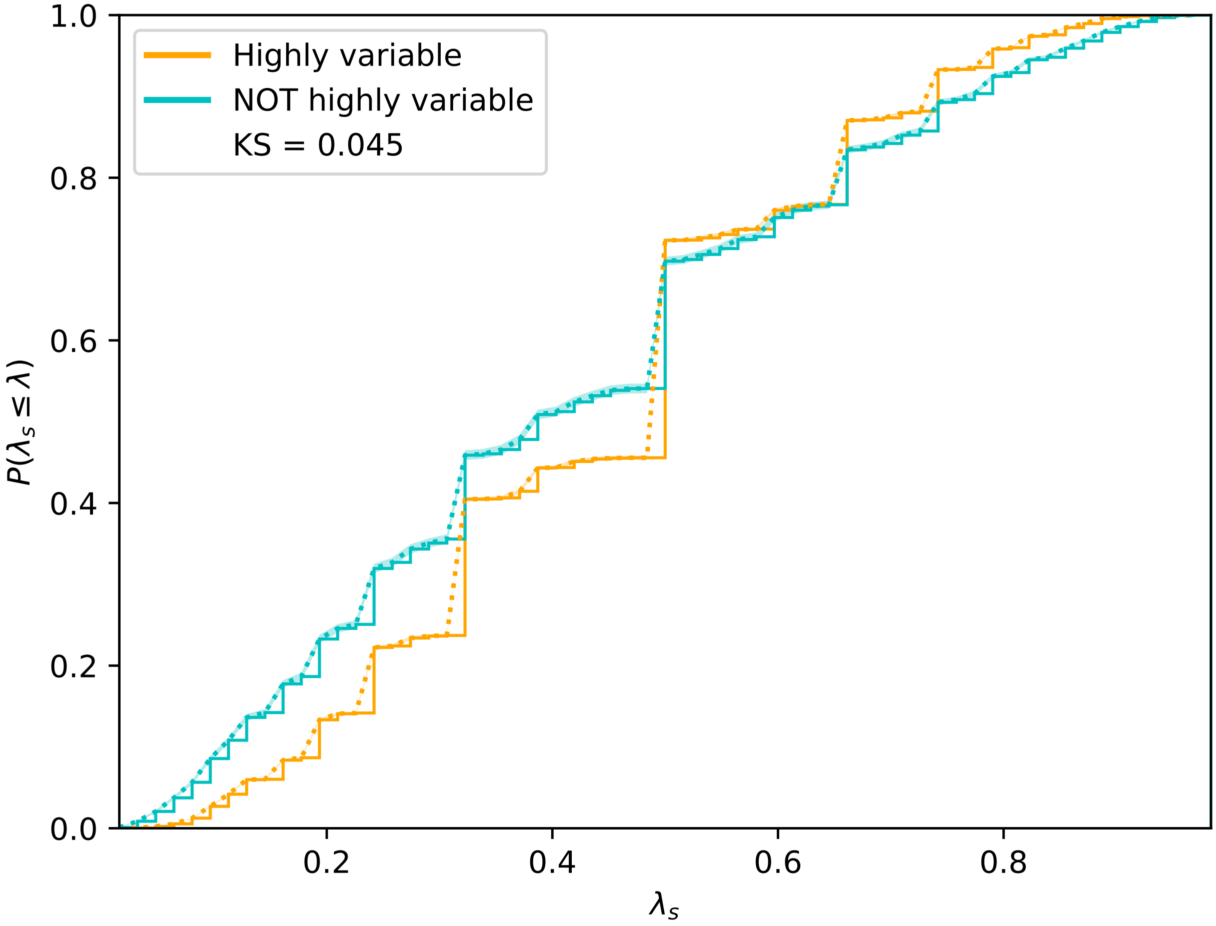

Supplementary Figure 74: Withdrawal sex reported.

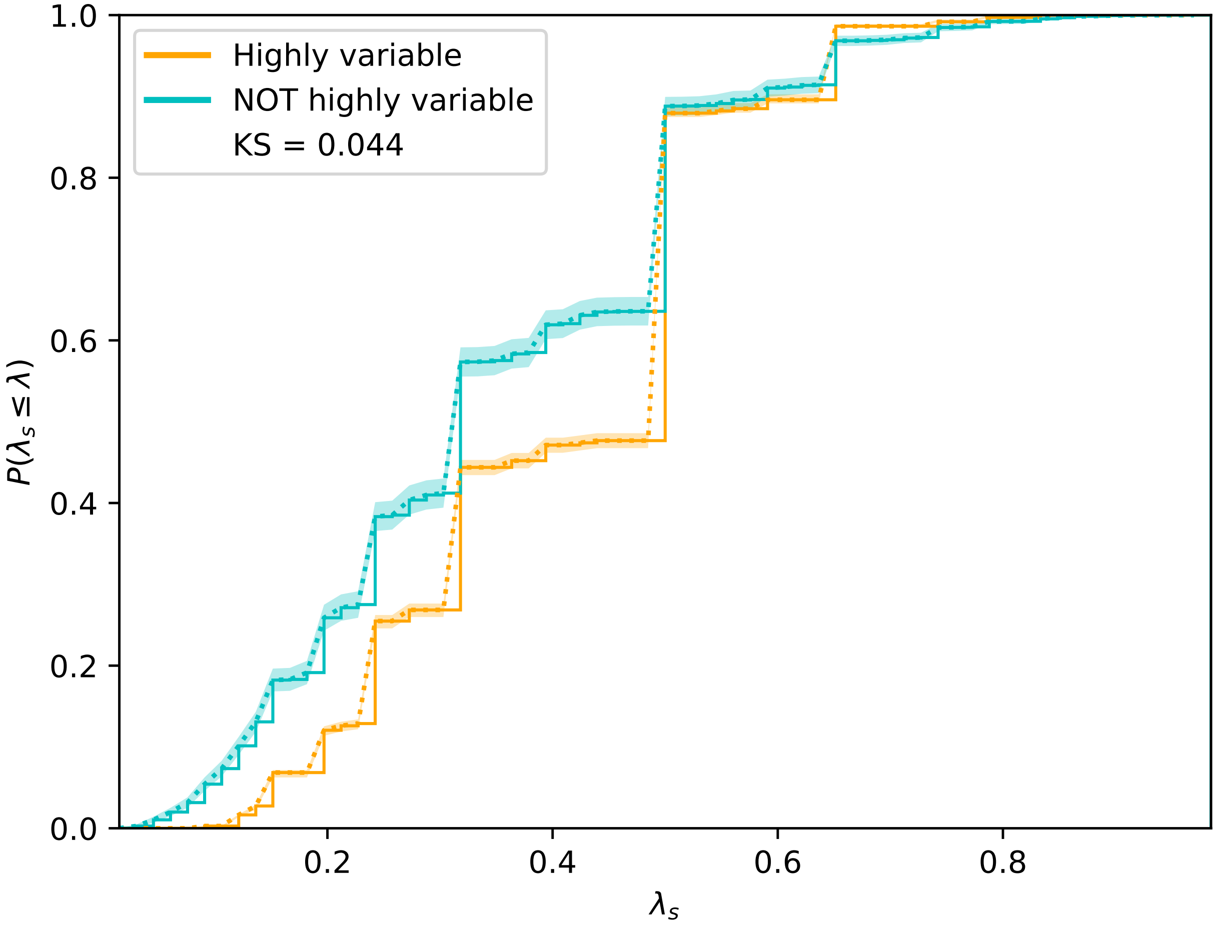

Supplementary Figure 75: Physical maladies: fever.

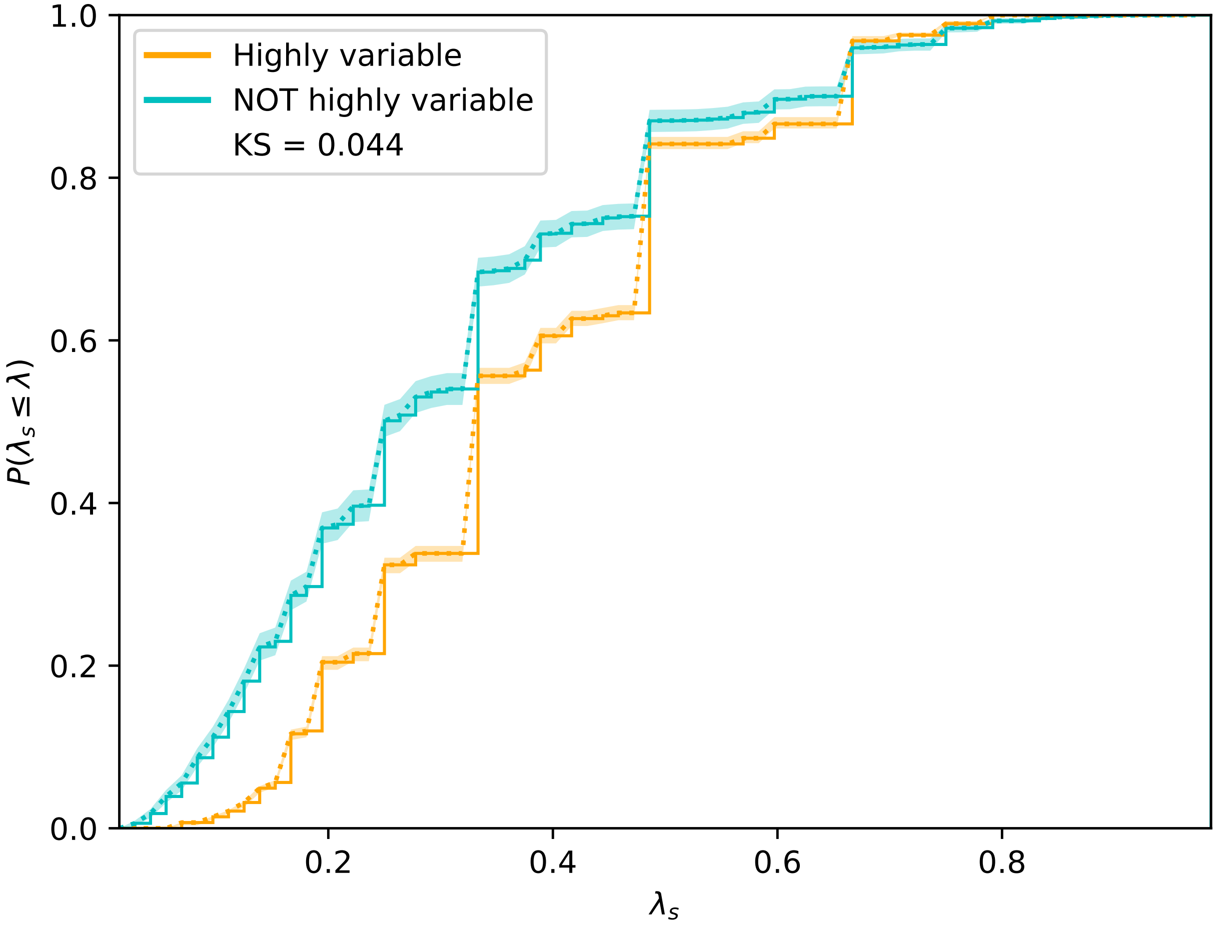

Supplementary Figure 76: Antibiotic medication taken.

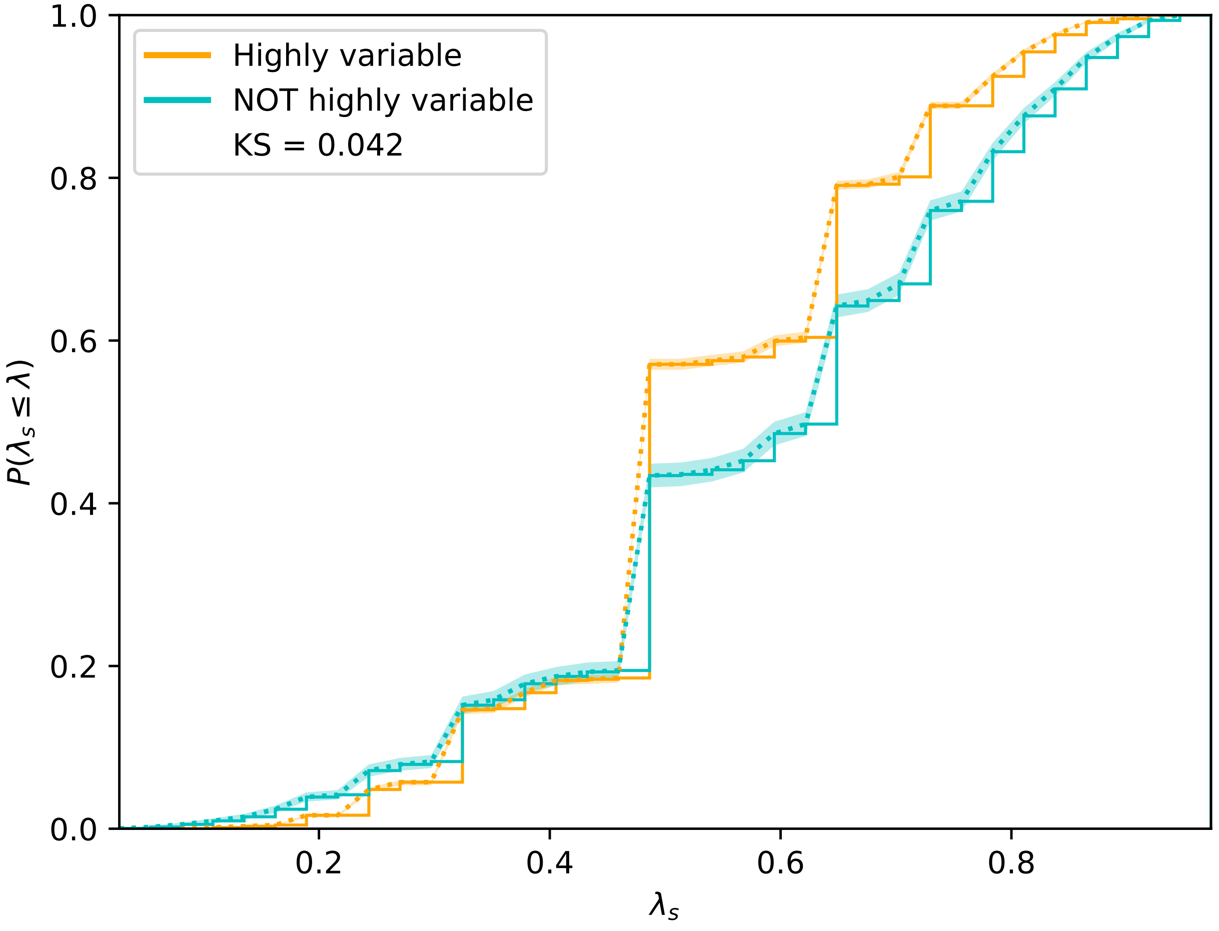

Supplementary Figure 77: Party-related experience: drinks party.

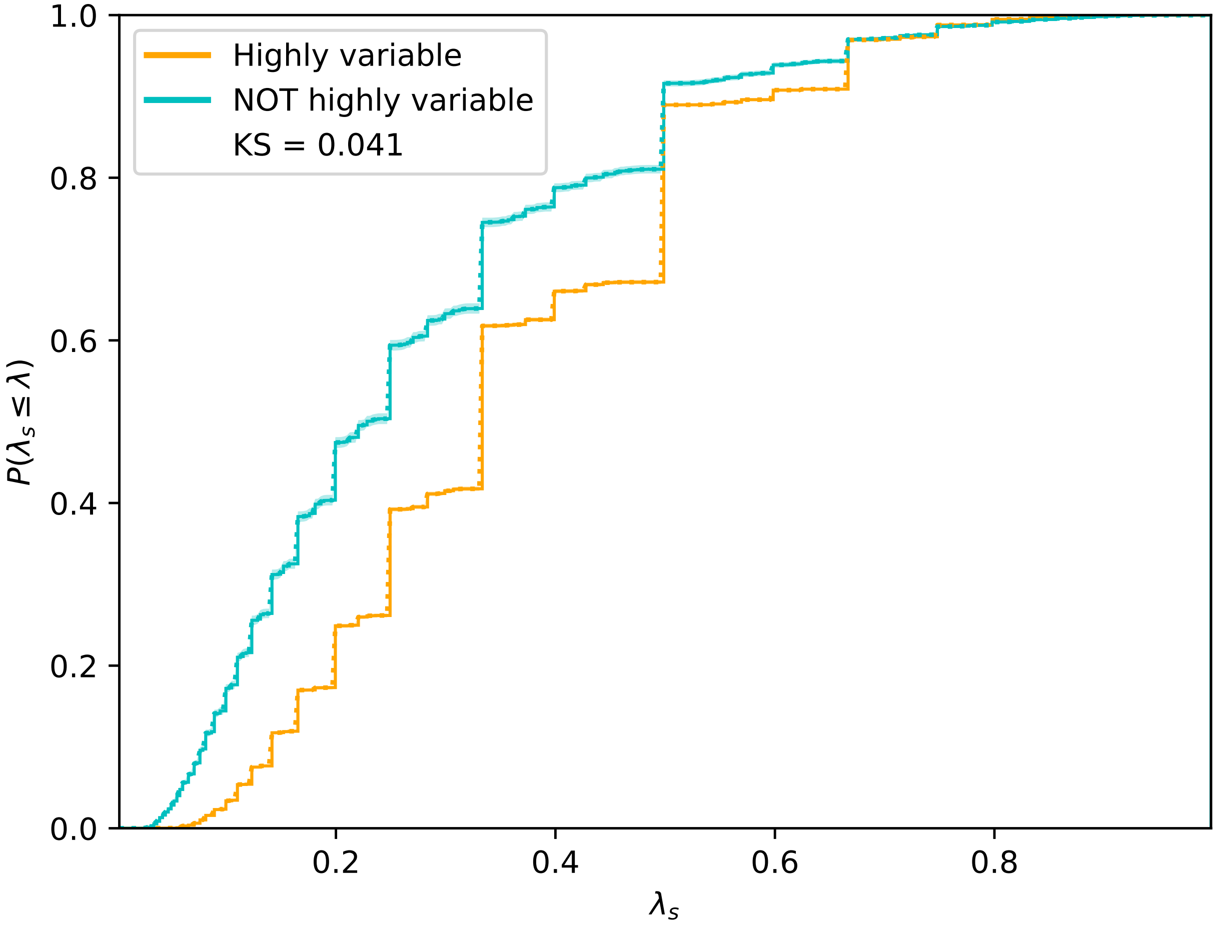

Supplementary Figure 78: 0-3 hours of sleep.

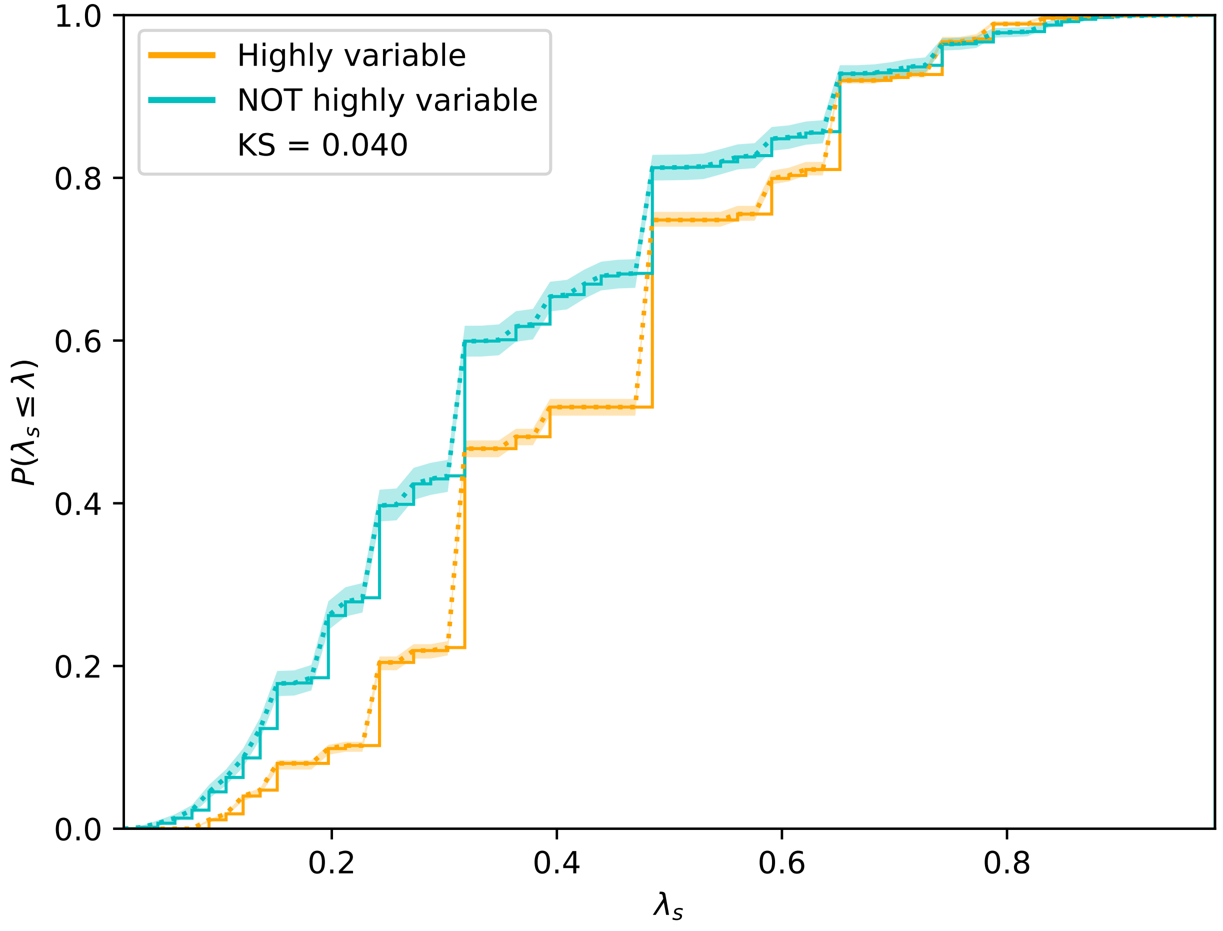

Supplementary Figure 79: Physical maladies: injury.

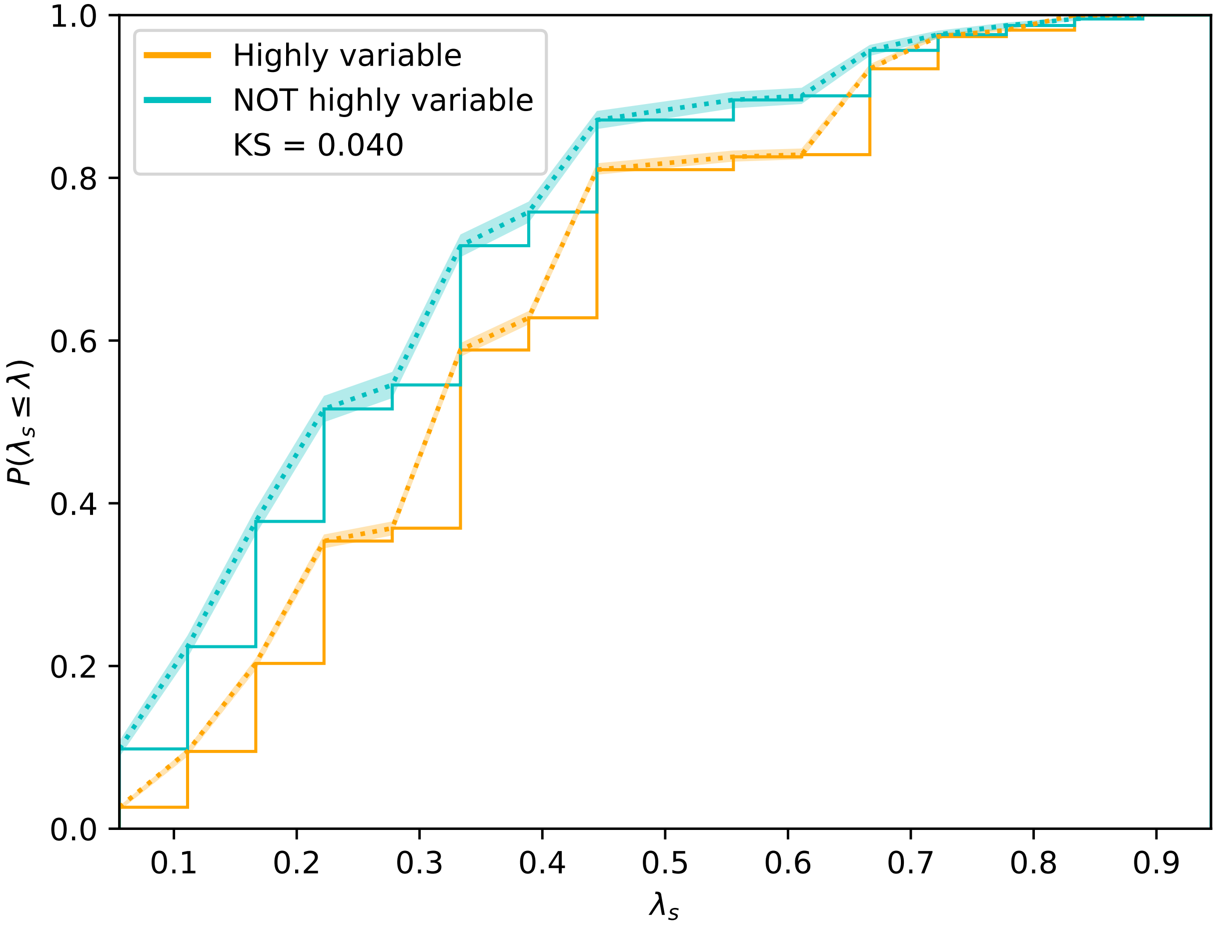

Supplementary Figure 80: Physical exercise: swimming.

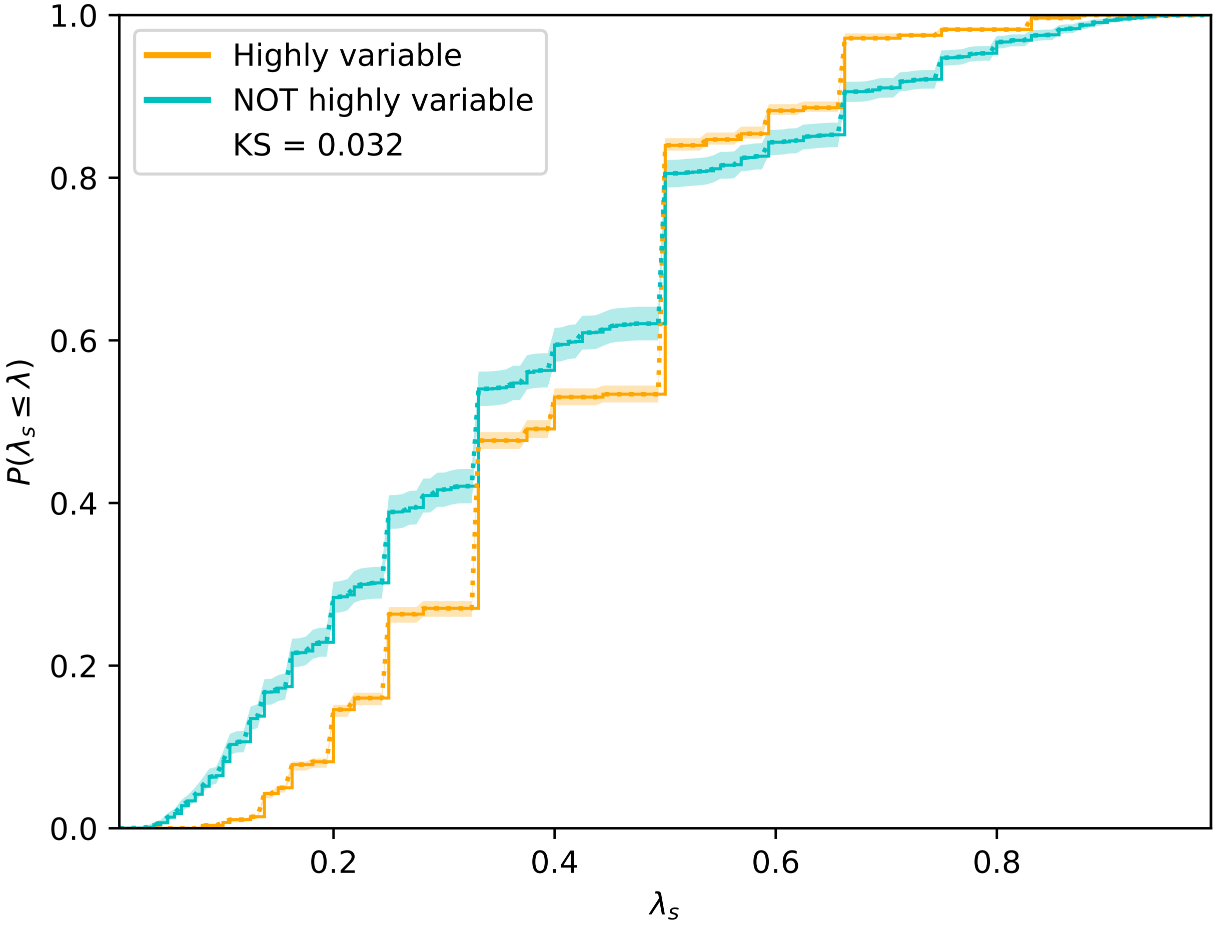

Supplementary Figure 81: Antihistamine medication taken.

STROBE checklist

Supplementary Table 13: STROBE Statement – Checklist of items that should be included in reports of cohort studies.

	Item No	Recommendation	Page No
**Title and abstract**			
	1	(a) Indicate the study's design with a commonly used term in the title or the abstract	2
	1	(b) Provide in the abstract an informative and balanced summary of what was done and what was found	2
**Introduction**			
Background/rationale	2	Explain the scientific background and rationale for the investigation being reported	2-3
Objectives	3	State specific objectives, including any prespecified hypotheses	3
**Methods**			
Study design	4	Present key elements of study design early in the paper	3-4
Setting	5	Describe the setting, locations, and relevant dates, including periods of recruitment, exposure, follow-up, and data collection	10-11
Participants	6	(a) Give the eligibility criteria, and the sources and methods of selection of participants. Describe methods of follow-up	10-11
		(b) For matched studies, give matching criteria and number of exposed and unexposed	10-11
Variables	7	Clearly define all outcomes, exposures, predictors, potential confounders, and effect modifiers. Give diagnostic criteria, if applicable	10-11
Data sources / measurement	8	For each variable of interest, give sources of data and details of methods of assessment (measurement). Describe comparability of assessment methods if there is more than one group	10-11
Bias	9	Describe any efforts to address potential sources of bias	12-13
Study size	10	Explain how the study size was arrived at	12
Quantitative variables	11	Explain how quantitative variables were handled in the analyses. If applicable, describe which groupings were chosen and why	11,14-16
Statistical methods	12	(a) Describe all statistical methods, including those used to control for confounding	12-13,16
		(b) Describe any methods used to examine subgroups and interactions	14-16
		(c) Explain how missing data were addressed	12-13
		(d) If applicable, explain how loss to follow-up was addressed	NA
		(e) Describe any sensitivity analyses	17
**Results**			
Participants	13	(a) Report numbers of individuals at each stage of study—eg numbers potentially eligible, examined for eligibility, confirmed eligible, included in the study, completing follow-up, and analysed	12
		(b) Give reasons for non-participation at each stage	11
		(c) Consider use of a flow diagram	12

	Item No	Recommendation	Page No
Descriptive data	14	(a) Give characteristics of study participants (eg demographic, clinical, social) and information on exposures and potential confounders	10-11
		(b) Indicate number of participants with missing data for each variable of interest	12
		(c) Summarise follow-up time (eg, average and total amount)	NA
Outcome data	15	Report numbers of outcome events or summary measures over time	25-26
Main results	16	(a) Give unadjusted estimates and, if applicable, confounder-adjusted estimates and their precision (eg, 95% confidence interval). Make clear which confounders were adjusted for and why they were included	4-8, 27
		(b) Report category boundaries when continuous variables were categorized	NA
		(c) If relevant, consider translating estimates of relative risk into absolute risk for a meaningful time period	NA
Other analyses	17	Report other analyses done—eg analyses of subgroups and interactions, and sensitivity analyses	4-8
**Discussion**			
Key results	18	Summarise key results with reference to study objectives	4-8
Limitations	19	Discuss limitations of the study, taking into account sources of potential bias or imprecision. Discuss both direction and magnitude of any potential bias	9-10
Interpretation	20	Give a cautious overall interpretation of results considering objectives, limitations, multiplicity of analyses, results from similar studies, and other relevant evidence	10
Generalisability	21	Discuss the generalisability (external validity) of the study results	9-10
**Other information**			
Funding	22	Give the source of funding and the role of the funders for the present study and, if applicable, for the original study on which the present article is based	17
